# Catching up? The educational mobility of migrants’ and natives’ children in Europe

**DOI:** 10.1080/00036846.2016.1267843

**Published:** 2016-12-30

**Authors:** Doris Oberdabernig, Alyssa Schneebaum

**Affiliations:** ^a^ World Trade Institute (WTI), University of Bern, Bern, Switzerland; ^b^ Department of Economics, Vienna University of Economics and Business, Vienna, Austria

**Keywords:** Intergenerational mobility, immigrants, educational attainment, Europe, J62, I21, J11

## Abstract

Migrants into European countries are often less educated than European natives. We analyse whether migrants’ children are more or less likely than natives’ children to achieve upward educational mobility across generations, and study differences in the factors, which contribute to differences in mobility for the two groups. We find that migrants’ descendants are more often upwardly mobile (and less often downwardly mobile) than their native peers in the majority of countries studied, and show that the main factor contributing to these patterns is the education level of parents. Although a lower parental education means that their children are less likely to access the same amount of human, social and financial capital as children of more highly educated parents, migrants’ descendants over the last two generations were able to make significant progress in reducing education gaps with natives.

## I. Introduction

The tremendous influx of immigrants into Europe over the past several decades, particularly of people from poorer and politically less stable countries, has been the source of large social and demographic shifts. A particular challenge of these shifts is that migrants into western European countries are typically less educated than people in the native population. If immigrants’ children are unable to surpass their parents’ education levels and get closer to reducing the gap between their own and natives’ education levels, migrant communities will remain at a disadvantage and more likely be a burden for their destination countries’ public welfare systems (see Barrett and McCarthy 2007; Blume and Verner 2007; Boeri 2010; Pellizzari 2013; Huber and Oberdabernig 2016). In this article, we examine the probability that descendants of native-born versus foreign-born parents reach higher education levels than their parents in 11 European countries. Further, we identify the drivers of the differences in intergenerational mobility for the two groups.

The literature on migrants’ (and their descendants’) educational attainment shows that migrant academic success is often significantly lower than that of native-born persons (as shown in Schütz, Ursprung, and Wößmann 2008; Schneeweis 2011; Dustmann, Frattini, and Lanzara 2012; Aydemir, Chen, and Corak 2013; Schneebaum, Rumplmaier, and Altzinger 2016; for example). In this article, we investigate whether this gap in achievement narrows across generations by studying differences in intergenerational mobility for the two groups. We are interested in knowing if second-generation migrants or natives in the last generation were more likely to reach higher education than their parents, and in particular, in explaining which factors drive these patterns. If the children of lower educated migrants were more likely to surpass their parents’ education level or make greater strides than their native peers, migrants’ children are less likely to remain disadvantaged across generations as they narrow the gap in educational attainment with natives.

A challenge for catching up in terms of education is that school performance is strongly related to one’s social and economic background.^1^ ^1^A large literature finds educational attainment to be strongly related to the socioeconomic background of an individual’s parents (see Becker and Tomes 1979, 1986; for theoretical foundations, Haveman and Wolfe 1995; for a review of the foundational empirical literature, and Dustmann, Rajah, and Soest 2003; Bauer and Riphahn 2007; Chevalier et al. 2013; for some recent studies on European countries). Migrants’ worse educational outcomes in many countries are due at least in part to the socioeconomic conditions related to their families having come from another country, in particular lower levels of financial, social and human capital (e.g. Riphahn 2003; Colding 2006; Schneeweis 2011; Lüdemann and Schwerdt 2013) and what Lüdemann and Schwerdt (2013) call ‘general inequalities’ which affect migrant students’ educational success. Educational upward mobility for migrants would thus come *in spite of* the circumstances around their migration background. Relatedly, though, much of the literature on the economics of immigration suggests that migrants might be more skilled than people who choose to stay in their country of birth, because more able and motivated people are the ones who self-select into migration (see Chiswick 1978, 1999; Borjas 1987, 1994).

The empirical literature on educational attainment shows that for the population as a whole, institutions such as preschool enrolment (the earlier, the better) (Schütz, Ursprung, and Wößmann 2008; Bauer and Riphahn 2009b), age of entry into school (again, the earlier the better) (Deming and Dynarski 2008; Bauer and Riphahn 2009a) and age of first tracking (later is better) (Dustmann 2004; Bauer and Riphahn 2006a; Hanushek and Wößmann 2006; Schütz, Ursprung, and Wößmann 2008; Wößmann 2009) all contribute to greater intergenerational mobility. ^2^ ^2^Also relevant in this literature are the findings from Fredriksson and Öckert (2005) and Puhani and Weber (2007), who show that school entry at an older age is correlated with greater academic achievement – although these findings may be related to the fact that older children are better performers because they are older and thus have had more time to accumulate skills. Elder and Lubotsky (2009) also measure student achievement and point to the importance of family background in academic success. Analyses which look instead more specifically at differences in educational mobility between immigrants and natives usually find that the educational outcome of migrants’ children is determined by their parents’ educational achievement to a lesser extent than it is for natives’ descendants, which is typically interpreted as greater educational mobility for migrants. Summary statistics in Gang and Zimmermann (2000), Dustmann (2008) and Yaman (2014) show that children of the on-average less educated migrants in Germany are more mobile than their native peers. Similarly, Bauer and Riphahn (2006b) find that in Switzerland, a greater percentage of children from low-educated migrants compared to low-educated natives are upwardly mobile, while Schneebaum, Rumplmaier, and Altzinger (2016) show that in Austria, these results are gender specific: second-generation sons are more likely than natives’ sons to be upwardly mobile, but that daughters of migrants are less likely than daughters of natives to be mobile. Finally, the results in Van Ours and Veenman (2003) suggest that controlling for the lower education of the immigrant parents renders the second-generation education gap statistically insignificant in the Netherlands. None of these papers go into a discussion of the drivers of the mobility differences, which is one of the contributions of this article.

Thus, while the existing body of literature has been very valuable for painting a picture of how intergenerational mobility differs by migration background and which institutions help mobility for the broader population, it has been quiet on the drivers of mobility differences between the migrant and native populations. An exception is a study by Bauer and Riphahn (2013), which looks at the institutional characteristics that differently affect the probability of mobility for migrants and natives in Switzerland. Bauer and Riphahn (2013) study the joint effects of institutional differences across Swiss cantons and find that it is early kindergarten entry which most positively affects the chances of intergenerational mobility for second-generation migrants, while later tracking and especially kindergarten attendance and early school entry positively affects the chances of upward mobility for natives. While the analysis in Bauer and Riphahn (2013) is at the level of *institutional* differences, it would also be useful to have an understanding of mobility drivers at the individual level, which we provide here.

The contribution of this article to the literature is thus threefold. First, in contrast to the majority of previous studies, we explicitly distinguish between educational upward and downward mobility, rather than interpreting the strength of intergenerational correlations or elasticities as the key indicator of mobility. Weak elasticities and correlations imply more intergenerational mobility, but cannot speak to the direction of the mobility. In order to be able to say whether intergenerational mobility for migrants is such that it is also closing the gap in educational attainment with natives, migrants’ mobility must be more likely to be *upward*. We thus analyse the differing probabilities of upward and downward mobility for the two populations, separately. In addition, we analyse the magnitude of upward mobility by comparing individuals whose educational attainment is two classes higher than those of their parents.

Second, we address the fact that in much of the empirical literature, the intergenerational correlations or elasticities of the migrant and native populations are compared without accounting for the fact that migrants are *a priori* more likely to be upwardly mobile, because their parents often have less education than natives’ parents. The lower educational attainment of the migrants’ parents gives second-generation migrants a lower threshold to pass in order to be upwardly mobile. Ignoring this fact could easily lead to a misinterpretation of the greater mobility observed for migrants in the literature. In our analysis, we account for the fact that migrant parents are often less educated than natives, and test for mobility differences that may exist once eliminating the difference in the thresholds to pass across populations. To do so, we compare the mobility patterns of children of natives and migrants with the same education level.

Finally, to our knowledge, we are the first to investigate which personal, parental and household-level characteristics are correlated with the mobility differences between natives’ and migrants’ children. In doing so, we provide some explanations of the mobility patterns that have been detected in many previous studies, but which have been left broadly unexplained. Knowing the characteristics which are related to the mobility differences between the two populations is important, because this information could be used to inform policy targeting educational advancement for one or the other groups specifically.

The results of our analysis confirm that in many European countries, migrants are more likely to be upwardly mobile (and less likely to be downwardly mobile) than natives.^3^ ^3^An exception are the Baltic countries, where migrant parents are more highly educated on average than native parents. We find that differences in the socioeconomic background between natives and immigrants explain a large part of the difference in mobility patterns. In particular, the weaker educational attainment of migrants’ parents – and the lower threshold for upward mobility it implies – is a central factor in why second-generation migrants appear to be more upwardly mobile in many countries. Once the differences in parental education are taken into account, second-generation migrants remain significantly more likely to be upwardly mobile only in Switzerland and in Luxembourg (while in the Czech Republic the opposite applies). In these three countries, the mobility gap is mainly driven by the size of the household when the respondents were 14 years old, as well as the age structure of second-generation migrants. Focusing on the extent of upward mobility, we find that second-generation migrants are more likely than natives’ children to surpass the education level of their parents by two education classes only in the United Kingdom, while in all other countries the differences are statistically insignificant.

This article is structured as follows. Section II summarizes the data sources and provides descriptive statistics on education levels of educational mobility. Section III introduces the methodologies used in this article and summarizes the results of the multivariate analyses. Section IV concludes the article.

## II. Data

We use data from the 2011 European Union Statistics on Income and Living Conditions (EU-SILC), which include information on each respondents’ parents, household circumstances when the respondents were 14, and standard demographic and socioeconomic information for respondents aged 25–59 in 2011.^4^ ^4^We base our analysis on EU-SILC data rather than on data from alternative sources (e.g. the European Social Survey, ESS; the Generations and Gender Survey, GGS; the Survey of Adult Skills, PIAAC; and the European Values Survey, EVS), because of the availability of more detailed information on educational attainment (in comparison to PIAAC), the availability of information on a person’s socioeconomic background (especially parental characteristics and information on household composition when the respondent was 14 years old), which is not available to the same extent in some other databases (in ESS, PIAAC), the larger coverage of European countries (in comparison to GGS), or the larger sample size (in comparison to EVS). Still, our sample size for second-generation migrations is quite small in some countries. Thus, we rely on Bayesian estimation methods throughout the article (see section ‘Logit estimations’). Upward (downward) mobility is a dummy variable equal to one if the respondent achieves a higher (lower) education level than their most highly educated parent, where education is measured in four categories (illiterate; ISCED 0–2; ISCED 3–4; ISCED 5–6).^5^ ^5^ISCED 0–2 corresponds to pre-primary through lower-secondary education, ISCED 3–4 to upper secondary through post-secondary non-tertiary education and ISCED 5–6 to tertiary education. We study the sub-population of respondents who have completed their education and who were born in the country of residence and form two groups: natives (those with at least one parent born in the same country as the respondent) and migrants’ descendants, or second-generation migrants (those whose parents were born in another country).^6^ ^6^We compare persons with at least one parent born in their country of residence to persons for whom this is not the case, assuming that the native born parent is able to help their child at school in a similar way as parents who are both native born. We exclude persons who did not yet finish their education and persons with missing information on their parents or missing values for any explanatory variable from the sample. For the analysis of upward mobility, we exclude respondents whose more highly educated parent has reached the highest education class because these persons can per definition not be upwardly mobile. Similarly, we exclude persons whose parents have the lowest education class in the analysis of downward mobility. Information on which share of observations is dropped in each step is available in Table A4 in Appendix A. The exclusion of observations due to missing information on parental characteristics or explanatory variables could lead to selection bias, which would arise if observations are non-randomly missing across the two groups. While we have no means to check the direction of potential bias for missing information on parental characteristics (such as education or country of birth), Table A5 in Appendix A provides an overview of the mobility differences between natives and migrants for observations that have been excluded because of missing information on explanatory variables. Table A5 (and Table E.2 in the online appendix) show the impact of different possibilities of observations being dropped due to missing observations.


A summary of the educational attainment of respondents and their parents is provided in Table 1.^7^ ^7^Descriptive statistics for the control variables that are used in the multivariate analysis for the full sample can be found in Table A1 in Appendix A. Table A3 provides an overview of the definitions of the variables. Native-born parents are more likely to have tertiary education than the migrants in all countries but Austria, Switzerland, Estonia and the United Kingdom, and are less likely to be illiterate in all countries but Latvia and Germany.^8^ ^8^Treating education levels as a cardinal scale, native parents are more educated than migrant parents on average in all countries but Estonia and Latvia, as indicated by the column labelled ‘average education’. The Baltic countries are likely to be different from the other countries included in our analysis because they regained independence from the Soviet Union only in the early 1990s. Thus, immigration into these countries was to a big part internal. Comparing the educational attainment of migrants and natives over generations, we can observe a decrease in the achievement gap between the two groups. In the parental generation, migrants were more likely than natives to be illiterate in all but two countries, but the children of migrants and natives are about equally (un)likely to be illiterate. Further, in Switzerland, Croatia and the United Kingdom, migrants’ children are even more likely to have a tertiary education than natives’ children, and in more than half of the countries (6 of 11) migrants’ children are more likely to have completed upper secondary education (ISCED 3–4). Thus, we begin to see evidence of greater educational mobility for descendants of migrants in many of the countries in our sample.

Taking a closer look at upward mobility, Table 2 shows the proportion of natives’ and migrants’ descendants, which is upwardly mobile for each country in our sample, given the highest education level of their parents. In all countries but Estonia and Latvia, a greater share of migrants’ children than natives’ children are in a higher education class than their parents, in total. Descendants of migrants with lower secondary education or less (ISCED 0–2) show higher mobility than native descendants with lower-secondary educated parents in all countries but Austria, the Czech Republic and Estonia. In most countries, children of low-educated migrants seem to be upwardly mobile more often than children of low-educated natives. In the former two countries where this is not the case (Austria and the Czech Republic), however, children of more highly educated parents with a migration background (with upper- or post-secondary education, ISCED 3–4) are more often upwardly mobile than those of natives. Indeed, children of migrants with upper- or post-secondary education are at least as mobile as descendants of similarly educated natives in half of the countries in our sample (Austria, Switzerland, the Czech Republic, France, Croatia and the United Kingdom).

**Table 1.** Education level by migration background.

	Parent					Descendant				
	Illiterate (%)	ISCED 0–2 (%)	ISCED 3–4 (%)	ISCED 5–6 (%)	Average education (%)	Illiterate (%)	ISCED 0–2 (%)	ISCED 3–4 (%)	ISCED 5–6 (%)	Average education
Natives and their descendants										
AT	0.0	33	52	16	1.83	0.0	11	66	24	2.13
BE	0.1	45	28	27	1.82	0.0	17	38	46	2.29
CH	0.1	15	69	15	2.00	0.0	5	65	30	2.25
CZ	0.0	56	33	11	1.54	0.0	6	76	17	2.11
DE	0.0	7	60	33	2.26	0.0	4	53	43	2.39
EE	0.0	27	49	24	1.98	0.0	11	56	33	2.22
FR	0.4	72	12	15	1.43	0.0	16	47	37	2.21
HR	0.1	53	39	8	1.55	0.0	20	64	16	1.97
LU	0.1	39	51	10	1.71	0.2	26	48	25	1.98
LV	0.2	35	46	19	1.83	0.3	13	55	31	2.17
UK	0.5	52	26	22	1.69	0.0	9	49	42	2.33
Migrants and their descendants										
AT	0.0	44	38	18	1.74	0.0	23	57	21	1.98
BE	9.5	69	17	5	1.17	0.0	22	50	27	2.05
CH	5.4	38	41	16	1.67	0.0	6	64	31	2.25
CZ	3.8	77	17	2	1.17	0.0	20	70	10	1.90
DE	0.0	27	50	23	1.96	0.0	3	56	41	2.39
EE	0.0	22	45	33	2.11	0.0	9	58	33	2.24
FR	9.3	76	8	7	1.12	0.0	17	50	33	2.16
HR	0.4	58	34	7	1.48	0.0	21	61	17	1.96
LU	0.6	67	25	8	1.40	0.3	23	55	22	1.98
LV	0.0	34	48	18	1.84	0.2	13	63	24	2.11
UK	2.7	58	18	22	1.58	0.0	3	41	56	2.53

Authors’ calculations are based on 2011 EU-SILC data. Parent and descendant stand for the education level of the highest educated parent or the respondent, respectively. In the empirical analysis on upward mobility, persons whose more highly educated parent reached the highest education level are dropped. In the analysis on downward mobility, persons whose parents are illiterate are dropped. Country-codes: AT = Austria, BE = Belgium, CH = Switzerland, CZ = Czech Republic, DE = Germany, EE = Estonia, FR = France, HR = Croatia, LU = Luxembourg, LV = Latvia, UK = United Kingdom.

**Table 2.** Upward mobility by parental education level and in total.

	Respondents’ upward mobility by parental education group							
	Native parents’ education				Migrant parent’s education			
	Illiterate (%)	ISCED 0–2 (%)	ISCED 3–4 (%)	Total (%)	Illiterate (%)	ISCED 0–2 (%)	ISCED 3–4 (%)	Total (%)
AT	–	79.8	22.1	44.4	–	63.8	27.5	47.1
BE	100	71.2	47.5	62.1	100	75.2	40.0	71.4
CH	100	83.8	27.1	37.5	100	92.9	27.2	61.6
CZ	100	90.0	23.9	65.8	100	76.5	27.8	68.9
DE	100	85.0	34.4	39.6	–	92.6	32.3	53.6
EE	–	83.6	29.4	48.5	–	82.3	26.3	44.8
FR	100	80.3	57.9	77.1	100	82.6	57.9	82.1
HR	100	68.3	21.2	48.3	100	70.1	28.4	54.8
LU	100	58.0	26.5	40.3	100	71.3	24.4	58.7
LV	75	79.8	31.6	52.4	–	83.5	20.5	46.7
UK	100	85.5	44.0	71.8	100	96.5	69.2	90.5

Authors’ calculations are based on 2011 EU-SILC data. The percentages indicate the proportion of upwardly mobile respondents given the (highest) education level of their parents. Respondents whose parents have reached the highest education level (ISCED 5–6) are excluded from the analysis. Country- codes: AT = Austria, BE = Belgium, CH = Switzerland, CZ = Czech Republic, DE = Germany, EE = Estonia, FR = France, HR = Croatia, LU = Luxembourg, LV = Latvia, UK = United Kingdom.

In what follows we examine how robust these differences are, once we compare descendants of migrants and natives from otherwise similar households, and study which characteristics drive the differences in mobility across migration background. To explore these issues, we turn to a multivariate analysis predicting mobility in section ‘Logit estimation’, and then decompose the detected mobility differences in section ‘Oaxaca–Blinder decomposition’.

## III. Empirical analysis

### Logit estimations

Taking the insights gained in the descriptive exploration of the data above, namely that children of the often lower educated group of migrants appear to be more mobile than their native counterparts in many countries, we move to a more structural analysis of these patterns and their causes. In a first step, using logit models, we investigate whether second-generation migrants are significantly more often mobile than natives once accounting for respondents’ socioeconomic backgrounds.

We use Bayesian estimation techniques throughout the article.^9^ ^9^We prefer Bayesian techniques to frequentist analyses because inference based on the latter is often complicated in small samples. For more information on Bayesian techniques see Koop (2003). Bayesian methods rely on Bayes’ rule, which is applied to learn about the parameters, 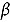, given the data, 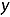

(1)
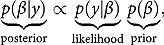

where the posterior density is proportional to the likelihood function times the prior density.^10^ ^10^For prior information we use three distinct specifications. One is an uninformative prior with mean and precision equal to 0. The other two prior specifications use information obtained from frequentist logit regressions. More specifically, we estimate logit regressions on the pooled samples of natives and second-generation migrants for each country, using the resulting parameter estimates as prior means and the inverse of the standard errors obtained from the frequentist estimations divided by 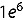 and 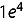, respectively, as prior precision. We rely on Gibbs sampling and run three Markov chains, one for each definition of the prior density, for which convergence is evaluated using Gelman and Rubin’s (1992) convergence diagnostic (the diagnostics can be found in the supplementary material (online appendix). Inference is based on a combination of the three chains, leading to a random sample from the posterior of 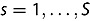 draws. Our estimations are conducted in R using the package ‘BayesLogit’ (see Polson, Scott, and Windle 2013; Polson and Scott 2011). For each model we run three Markov chains with 20,000 iterations each, after a burn-in of 5000 and a thinning of 100, resulting in 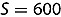.


The posterior mean for variable 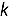, is calculated as

(2)
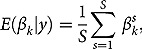

where *S* is the number of draws from the posterior. *p*-Values for each parameter, 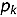, can be derived either as

(3)
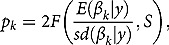

where 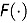 is the distribution function of the Student’s *t*-distribution with 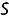 degrees of freedom^11^ ^11^We adjust 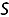 for potential autocorrelation in the Markov chain. and

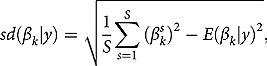

or alternatively, we can obtain the *p*-values numerically as

(4)
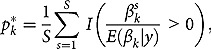

where 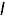 is an indicator function.^12^ ^12^That is, counting the share of draws for which 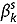 is equally signed as its posterior mean 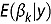.


#### Logit results

The results of the (Bayesian) logit estimations that explain educational upward mobility are reported in Table 3. In a first, naïve approach, we account for a respondent’s migration background by including a dummy variable that is equal to one if the respondent’s parents are born in another country as the single regressor. The results are reported in the upper panel of the table. The results suggest that migrants’ descendants have a significantly higher probability of being upwardly mobile than their native counterparts in seven countries (Belgium, Switzerland, Germany, France, Croatia, Luxembourg and the United Kingdom). Only in Latvia are migrants’ children less likely to be upwardly mobile than natives’ children. For Austria, the Czech Republic and Estonia, we do not detect a statistically significant difference between the mobility rates of the migrant and native samples.

**Table 3.** Logit results for pooled sample.

	Austria	Belgium	Switzerland	Czech Republic	Germany	Estonia	France	Croatia	Luxembourg	Latvia	UK
Naive model with migration background dummy only											
	0.029	0.092 **	0.238 ***	0.031	0.143 ***	−0.041	0.049 **	0.067 *	0.183 ***	−0.056 **	0.187 ***
	(0.581)	(0.024)	(0.000)	(0.494)	(0.000)	(0.207)	(0.014)	(0.050)	(0.000)	(0.041)	(0.000)
Model with additional control variables											
Migrant background (d)	−0.047	0.065	0.042	−0.050	0.008	−0.037	0.030	0.047	0.047	−0.043 **	0.177 ***
	(0.278)	(0.119)	(0.124)	(0.141)	(0.848)	(0.170)	(0.146)	(0.105)	(0.102)	(0.050)	(0.000)
Birth year	−0.003	0.000	−0.003	0.004 ***	−0.004 **	−0.002	0.005 ***	0.000	0.003	0.002	0.003 *
	(0.147)	(0.994)	(0.211)	(0.005)	(0.045)	(0.367)	(0.001)	(0.880)	(0.196)	(0.243)	(0.080)
Cohort 50s (d)	−0.086 **	−0.120 **	−0.085 **	0.017	−0.070 *	0.078	−0.046	−0.005	−0.077	0.186 ***	−0.035
	(0.031)	(0.038)	(0.040)	(0.600)	(0.066)	(0.146)	(0.181)	(0.918)	(0.147)	(0.000)	(0.447)
Cohort 60s (d)	−0.021	−0.009	−0.048 *	0.037 *	−0.041	0.080 **	−0.001	0.045	−0.059	0.132 ***	−0.004
	(0.450)	(0.813)	(0.087)	(0.068)	(0.102)	(0.013)	(0.959)	(0.116)	(0.102)	(0.000)	(0.881)
Male (d)	0.085 ***	−0.050 ***	0.141 ***	0.032 ***	0.092 ***	−0.166 ***	0.022 **	0.060 ***	0.076 ***	−0.175 ***	−0.003
	(0.000)	(0.004)	(0.000)	(0.001)	(0.000)	(0.000)	(0.025)	(0.000)	(0.000)	(0.000)	(0.823)
Mother’s age at birth	0.018 *	0.024 *	0.025 **	0.004	0.024 **	0.006	0.018 **	−0.001	0.046 ***	0.014	0.027 ***
	(0.054)	(0.053)	(0.035)	(0.623)	(0.030)	(0.604)	(0.022)	(0.899)	(0.002)	(0.177)	(0.008)
Mother’s age at b., sq (/100)	−0.034 **	−0.043 **	−0.036 *	−0.010	−0.045 **	−0.010	−0.035 ***	−0.005	−0.080 ***	−0.020	−0.042 **
	(0.030)	(0.027)	(0.065)	(0.534)	(0.013)	(0.596)	(0.009)	(0.799)	(0.001)	(0.220)	(0.013)
Father’s age at birth	0.010	0.020 *	0.021 **	0.003	0.013	0.005	0.004	0.024 ***	0.012	0.002	−0.007
	(0.146)	(0.067)	(0.031)	(0.707)	(0.131)	(0.539)	(0.574)	(0.006)	(0.303)	(0.845)	(0.412)
Father’s age at b., sq (/100)	−0.007	−0.020	−0.029 **	0.001	−0.005	−0.006	0.001	−0.024 *	−0.005	0.002	0.014
	(0.475)	(0.196)	(0.039)	(0.918)	(0.725)	(0.640)	(0.930)	(0.063)	(0.775)	(0.858)	(0.252)
Age difference parents	−0.004	−0.010 **	−0.000	−0.001	−0.009 ***	−0.004	−0.004 *	−0.006 *	−0.005	−0.005 **	−0.004
	(0.168)	(0.021)	(0.894)	(0.788)	(0.005)	(0.189)	(0.081)	(0.066)	(0.207)	(0.036)	(0.191)
Mother out of labour f. (d)	0.003	−0.040 **	0.012	−0.064 ***	−0.006	0.005	−0.012	0.002	0.024	0.026	−0.012
	(0.810)	(0.027)	(0.423)	(0.000)	(0.654)	(0.864)	(0.234)	(0.883)	(0.170)	(0.348)	(0.405)
Mother’s education	0.090 ***	0.077 ***	0.069 ***	0.075 ***	0.065 ***	0.143 ***	0.063 ***	0.163 ***	0.122 ***	0.174 ***	0.016
	(0.000)	(0.005)	(0.000)	(0.000)	(0.000)	(0.000)	(0.004)	(0.000)	(0.000)	(0.000)	(0.479)
Father’s education	0.049 *	0.071 **	0.029	0.074 ***	0.008	0.069 ***	0.041 *	0.156 ***	0.119 ***	0.104 ***	0.002
	(0.090)	(0.026)	(0.253)	(0.000)	(0.778)	(0.003)	(0.071)	(0.001)	(0.007)	(0.000)	(0.958)
Highest parental educ.	−0.541 ***	−0.376 ***	−0.612 ***	−0.525 ***	−0.595 ***	−0.569 ***	−0.255 ***	−0.493 ***	−0.672 ***	−0.496 ***	−0.254 ***
	(0.000)	(0.000)	(0.000)	(0.000)	(0.000)	(0.000)	(0.000)	(0.000)	(0.000)	(0.000)	(0.000)
# of children in hh	−0.008 *	−0.019 ***	−0.011 **	−0.030 ***	−0.011 *	−0.019 ***	−0.017 ***	−0.019 ***	−0.009	−0.023 ***	−0.019 ***
	(0.082)	(0.000)	(0.045)	(0.000)	(0.063)	(0.003)	(0.000)	(0.001)	(0.121)	(0.000)	(0.000)
# of adults in hh	−0.012 **	−0.018 **	−0.031 ***	−0.023 ***	−0.008	−0.024 **	−0.023 ***	0.000	−0.017 **	−0.023 *	−0.043 ***
	(0.027)	(0.043)	(0.000)	(0.003)	(0.188)	(0.032)	(0.000)	(0.993)	(0.047)	(0.060)	(0.000)
Financial situation	0.030 *	0.038 *	−0.002	0.017	−0.008	0.045	0.036 **	0.082 ***	−0.007	0.058 **	0.047 **
	(0.099)	(0.086)	(0.957)	(0.274)	(0.845)	(0.225)	(0.016)	(0.000)	(0.794)	(0.019)	(0.018)
Finance * highest educ.	−0.004	0.001	0.004	−0.006	0.006	−0.008	−0.015	−0.043 ***	0.034 *	−0.030 **	−0.025 **
	(0.711)	(0.945)	(0.812)	(0.572)	(0.770)	(0.711)	(0.215)	(0.000)	(0.068)	(0.046)	(0.049)
*N*	3990	3051	3736	5518	5514	2617	6886	4243	2835	3468	3582

This table reports average marginal effects. The constant term is not shown. *p*-Value based on Equation (3) is in parentheses. ***, **, * signify significance of the effects at the 1%, 5%, 10% level, respectively, based on Equation (4). (d) indicates that the variable is a dummy variable. For dummy variables, the reported effect refers to a discrete change of the variable from 0 to 1.

Next, in order to investigate whether the detected difference in upward mobility between the two groups is accounted for by a person’s socioeconomic background, we control for individual, parental and household characteristics, as well as potential cohort effects. In particular, we consider the respondents’ birth year and gender; their parents’ characteristics (parents’ age at childbirth and its square, parental age difference and an indicator of whether the mother was out of labour force when the respondent was 14); parental education (education levels of both parents and of the more highly educated parent)^13^ ^13^With including the education level of the more highly educated parent additionally, we account for the fact that if the parents already have a high education level it is harder for a person to surpass this level than if the parents have a very low education level. We expect the coefficient of this education threshold effect to be negative.; household characteristics at age 14 (number of adults and children in the household); and the household financial situation at age 14 (financial situation of the household and its interaction with the highest parental education level). We add two dummy variables equal to one if a respondent was born in the 50s or 60s, respectively, with the 70s and 80s being the base category.^14^ ^14^The respondents in our sample are all born between 1951 and 1986 (around 31% in the 50s, 34% in the 60s and 36% in the 70s or 80s). The results are reported in the lower panel of Table 3.

Once we control for a person’s socioeconomic background, the mobility gap between natives’ and migrants’ descendants turns insignificant everywhere but in Latvia and the United Kingdom. Thus, we find that in most countries, individual, parental and household characteristics are important drivers of the observed mobility patterns. While the sign of the effects of birth year, cohort and gender on the probability of being upwardly mobile is country-specific, an older age of the mother at childbirth has a significantly positive effect on the probability of her children reaching a higher education than their parents, however with decreasing rate (as indicated by the negative effect of the squared term). A similar pattern is visible for the father’s age at childbirth, however in a smaller number of countries and to a quantitatively lesser extent. The effect of a larger age difference between the respondent’s parents is consistently negative, whenever it is statistically significant. The mother’s labour market status when the respondent was 14 years old plays a significant role in Belgium and the Czech Republic – being out of the labour force contributes to a lower probability of upward mobility in these countries. Very striking is the positive and statistically significant effect of a higher education level of both the mother and the father, which is in line with the findings of the large literature that finds that a higher parental education has a positive impact on the education level of children. The education level of the more highly educated parent, which picks up the education threshold effect, has a negative coefficient, as expected. Furthermore, a bigger household size when the respondent was 14 years old is significantly negatively related to educational upward mobility in most countries, as evidenced by the negative parameters for the number of children and the number of adults in the household. Finally, a better financial situation of the household when the respondent was young is connected to a higher educational upward mobility. This relationship becomes smaller, though, as the education level of the more highly educated parent increases.^15^ ^15^This finding suggests that offspring of financially well-endowed parents with lower education levels are especially eager to obtain a higher education level, while this might not be true to the same extent for persons with relatively highly educated parents. Luxembourg is an exception, as especially children of highly educated parents with a good financial situation are more often upwardly mobile.


In order to obtain a more conclusive picture of why the observed pattern in Table 3 emerges and the difference in upward mobility between migrants’ and natives’ children turns insignificant once controlling for their socioeconomic background, we go one step further and try to shed light on which factors are responsible for explaining the higher upward mobility of migrants’ descendants in most countries (and their lower mobility in Latvia, respectively).

### Oaxaca–Blinder decomposition

A prominent means for finding out which characteristics explain the observed differences between two groups, which received great attention especially in the labour literature, is the use of Oaxaca–Blinder decompositions (Blinder 1973; Oaxaca 1973). The decomposition allows researchers to attribute that part of the mobility gap which can be explained by observable characteristics to individual variables (or variable groups) and provide a clearer picture of the mechanisms at work. It also allows for the identification of that part of the gap which stems from unobserved factors or differences in returns to characteristics between the two groups.

For performing the Oaxaca–Blinder decompositions of educational mobility, we first estimate country-specific logit models for migrants’ and natives’ descendants separately. Introducing notation, the logit model can be written as

(5)
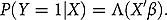

Equation (5) links the probability of educational upward mobility with respect to the most highly educated parent (i.e. 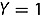) to the covariates 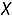, where 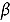 is the vector of posterior means (from Equation (2)), and 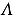 is the distribution function of the logistic distribution.

Standard references for the Oaxaca–Blinder decomposition for nonlinear models are Yun (2004) and Fairlie (2005), who suggest decomposing the mobility gap between migrants, 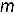, and natives, 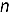, (the base group) into

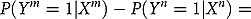

(6)
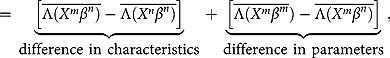

where 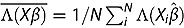 and 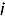 is an individual. To find the contribution of covariate 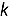 to the difference in characteristics, 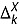 (i.e. differences in upward mobility that can be explained by differences in observable characteristics between natives’ and migrants’ descendants), we follow Kaiser (2015), who shows that the detailed decomposition can be derived as^16^ ^16^Yun (2004) proposes an alternative methodology to isolate the contribution of each covariate to the aggregate decomposition, which is based on the evaluation of covariates at their means. We follow the methodology in Kaiser (2015) because it allows us to take into account the nonlinear nature of logit models by accounting for differences also in higher order moments of X.

(7)
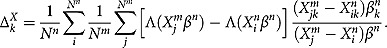

To overcome the identification problem present in evaluating the effect of dummy variables in the detailed decomposition (see Yun 2005), we include dummy variables only in variable groups (e.g. we report the effect of all cohort dummies together rather than reporting the effect of each cohort separately).

### Decomposition results

#### Upward mobility

Table 4 shows the results of this decomposition analysis for the probability of respondents of obtaining a higher education level than their more highly educated parent. The upper panel of the table reports the upward mobility of second-generation *migrants* and *natives* and the difference in mobility between the two groups (*mobility gap*). It also shows how much of this gap can be explained by differences in observable characteristics between the groups (*explained* part) and which part remains *unexplained*. The lower panel of the table shows the contribution of individual variable groups to the part of the mobility gap that stems from observable characteristics.^17^ ^17^To keep the interpretation simple, we form groups of individual variables and report the contribution of each group to the part of the mobility gap that is based on the difference in observable characteristics. The results of the detailed decomposition, which summarizes the contribution of each individual variable, are reported in Table B3 in Appendix B. Specifically, the variable groups comprise *birth cohort* (including birth year and cohort dummies); *gender; parents*’ characteristics (parents’ age at birth, its square, parental age difference and the mother’s labour market status); *education* (education level of both parents and of the more highly educated parent); *household* characteristics (number of adults and children in the household when the respondent was 14); and *finance* (financial situation of the household when the respondent was 14 and its interaction with the highest parental education level). Apart from reporting the contribution of each variable group to the mobility gap and the associated level of statistical significance, the upper panel of the table also summarizes the share of second-generation migrants and natives whose more highly educated parent did not yet reach the highest education level (see the percentages in square brackets).

**Table 4.** Decomposition results for upward mobility.

	Austria	Belgium	Switzerland	Czech Republic	Germany	Estonia	France	Croatia	Luxembourg	Latvia	UK
Probability of upward mobility											
Migrants	47.126 ***	71.429 ***	61.566 ***	68.932 ***	53.595 ***	44.770 ***	82.143 ***	54.795 ***	58.735 ***	46.699 ***	90.517 ***
	(0.000)	(0.000)	(0.000)	(0.000)	(0.000)	(0.000)	(0.000)	(0.000)	(0.000)	(0.000)	(0.000)
	82%	95%	84%	98%	77%	67%	93%	93%	92%	82%	78%
Natives	44.427 ***	62.075 ***	37.540 ***	65.762 ***	39.619 ***	48.486 ***	77.127 ***	48.335 ***	40.272 ***	52.435 ***	71.754 ***
	(0.000)	(0.000)	(0.000)	(0.000)	(0.000)	(0.000)	(0.000)	(0.000)	(0.000)	(0.000)	(0.000)
	84%	73%	85%	89%	67%	76%	85%	92%	90%	81%	78%
Mobility gap	2.699	9.354 **	24.026 ***	3.170	13.975 ***	−3.716	5.016 **	6.460 *	18.463 ***	−5.736 **	18.763 ***
	(0.616)	(0.026)	(0.000)	(0.502)	(0.001)	(0.273)	(0.017)	(0.063)	(0.000)	(0.029)	(0.000)
Explained	7.684 ***	3.030 **	20.393 ***	8.968 ***	13.147 ***	0.065	1.827 ***	1.888 ***	14.102 ***	−1.201 ***	3.050 ***
	(0.000)	(0.011)	(0.000)	(0.000)	(0.000)	(0.873)	(0.001)	(0.000)	(0.000)	(0.000)	(0.000)
Unexplained	−4.985	6.323 *	3.633 *	−5.798	0.828	−3.782	3.190 *	4.571	4.361 *	−4.535 **	15.713 ***
	(0.168)	(0.053)	(0.081)	(0.108)	(0.776)	(0.109)	(0.063)	(0.130)	(0.087)	(0.025)	(0.000)
Contribution of covariates to differences in characteristics (explained part)											
Birth cohort	−0.027	1.266 ***	0.617	−1.848 ***	−0.002	0.216 ***	2.276 ***	0.311	4.691 ***	0.835 ***	1.084 ***
	(0.958)	(0.008)	(0.104)	(0.000)	(0.944)	(0.000)	(0.000)	(0.194)	(0.000)	(0.000)	(0.000)
Gender	−0.446 ***	−0.065 **	−0.180 ***	−0.052 ***	−0.142 ***	−0.560 ***	0.086 **	−0.109 ***	0.277 ***	−1.543 ***	−0.008
	(0.000)	(0.011)	(0.000)	(0.009)	(0.000)	(0.000)	(0.024)	(0.000)	(0.000)	**(0.000)**	(0.912)
Parents	−0.122	−1.827 ***	−0.415	−0.745 **	1.205 ***	0.330	0.112	0.700 ***	−1.466 ***	0.516 **	0.254
	(0.555)	(0.003)	(0.114)	(0.026)	(0.000)	(0.313)	(0.733)	(0.009)	(0.000)	(0.017)	(0.343)
Education	7.240 ***	6.050 ***	20.083 ***	15.028 ***	12.551 ***	−0.715 **	1.260 *	1.105 ***	12.772 ***	−0.842 ***	3.767 ***
	**(0.000)**	**(0.009)**	**(0.000)**	**(0.000)**	**(0.000)**	(0.011)	(0.083)	**(0.001)**	**(0.000)**	(0.000)	**(0.000)**
Household	0.313 **	−1.110 ***	1.094 ***	−3.990 ***	−0.059	0.772 **	−2.406 ***	−0.399 *	0.287	0.213 ***	−3.573 ***
	(0.015)	(0.002)	(0.002)	(0.000)	(0.326)	**(0.011)**	**(0.000)**	(0.096)	(0.151)	(0.001)	(0.000)
Finance	0.727	−1.284	−0.807	0.575	−0.403	0.046	0.493	0.281	−2.466	−0.403	1.526 *
	(0.384)	(0.497)	(0.793)	(0.686)	(0.858)	(0.680)	(0.389)	(0.174)	(0.358)	(0.101)	(0.065)
*N*	3990	3051	3736	5518	5514	2617	6886	4243	2835	3468	3582
*N* natives	3903	2911	3455	5415	5361	2378	6466	4024	2503	3059	3466
*N* migrants	87	140	281	103	153	239	420	219	332	409	116

This table shows the percentage of upwardly mobile migrants’ and natives’ descendants and the contribution of characteristics to the difference between the groups. Migrants (natives) refers to the upward mobility of migrants’ (natives’) descendants. Explained (unexplained) refers to the part of the mobility gap arising from differences in characteristics (parameters). The *p*-value based on Equation (3) is shown in parentheses. ***, **, * signify significance of the effects at the 1%, 5%, 10% level, respectively, based on Equation (4). The percentages in the square brackets indicate which share of the total sample of natives’ and migrants’ descendants is included in the analysis. The remaining share is excluded because the highest educated parent of these respondents already reached the highest education level. The variable group with the largest contribution to the explained part of mobility gap in each country is highlighted in bold.

**Table 5.** Decomposition results for downward mobility.

	Austria	Switzerland	Germany	Estonia	France	Croatia	Luxembourg	Latvia
Probability of downward mobility								
Migrants	15.094 ***	6.309 ***	7.035 ***	20.728 ***	2.696 ***	6.383 ***	6.704 ***	14.800 ***
	(0.000)	(0.000)	(0.000)	(0.000)	(0.001)	(0.000)	(0.000)	(0.000)
	100%	94.6%	100%	100%	90.7%	99.6%	99.4%	100%
Natives	11.425 ***	8.288 ***	14.519 ***	16.169 ***	4.589 ***	6.470 ***	13.147 ***	13.410 ***
	(0.000)	(0.000)	(0.000)	(0.000)	(0.000)	(0.000)	(0.000)	(0.000)
	100%	99.9%	100%	100%	99.6%	99.9%	99.9%	99.8%
Mobility gap	3.669	−1.979	−7.484 ***	4.559 **	−1.893 *	−0.087	−6.443 ***	1.390
	(0.242)	(0.215)	(0.003)	(0.028)	(0.072)	(0.958)	(0.000)	(0.394)
Explained	0.280	−0.740 ***	−3.505 ***	0.611 *	−2.235 ***	−0.731 **	−5.458 ***	−0.322
	(0.370)	(0.001)	(0.000)	(0.081)	(0.000)	(0.011)	(0.000)	(0.155)
Unexplained	3.389	−1.239	−3.979 ***	3.948 **	0.342	0.644	−0.984	1.712
	(0.151)	(0.275)	(0.001)	(0.025)	(0.486)	(0.754)	(0.380)	(0.205)
Contribution of covariates to differences in characteristics (explained part)								
Birth cohort	0.176	−0.470 ***	−0.175 **	−0.759 ***	−0.048 **	−0.090	−1.710 ***	−0.511 ***
	(0.113)	**(0.000)**	(0.013)	(0.000)	(0.049)	(0.241)	(0.000)	**(0.000)**
Gender	0.122 **	−0.087 ***	−0.040 ***	−0.198 ***	−0.038 ***	−0.012	−0.128 ***	0.499 ***
	(0.010)	(0.000)	(0.000)	(0.000)	(0.004)	(0.469)	(0.001)	(0.000)
Parents	−0.084	0.155 *	−0.260 ***	−0.138	−0.083	−0.188	0.360 *	−0.036
	(0.647)	(0.067)	(0.002)	(0.360)	(0.232)	(0.230)	(0.092)	(0.743)
Education	0.339 ***	−0.138	−2.054 ***	1.909 ***	−2.421 ***	−1.576 ***	−8.498 ***	−0.005
	**(0.001)**	(0.129)	**(0.000)**	**(0.007)**	**(0.000)**	**(0.000)**	**(0.000)**	(0.970)
Household	−0.065	−0.262 **	0.006	−0.197	0.021	0.844 ***	−0.040	−0.218 ***
	(0.332)	(0.045)	(0.895)	(0.351)	(0.808)	(0.000)	(0.676)	(0.000)
Finance	−0.207 *	0.062	−0.982 **	−0.014	0.335	0.291 *	4.558 ***	−0.044
	(0.056)	(0.401)	(0.020)	(0.982)	(0.368)	(0.099)	(0.000)	(0.322)
*N*	4745	4395	8154	3505	8013	4609	3142	4251
*N* natives	4639	4078	7955	3148	7605	4374	2784	3751
*N* migrants	106	317	199	357	408	235	358	500

This table shows the percentage of downwardly mobile migrants’ and natives’ descendants and the contribution of characteristics to the difference between the groups. Migrants (natives) refers to the downward mobility of migrants’ (natives’) descendants. Explained (unexplained) refers to the part of the mobility gap arising from differences in characteristics (parameters). The *p*-value based on Equation (3) is shown in parentheses. ***, **, * signify significance of the effects at the 1%, 5%, 10% level, respectively, based on Equation (4). The percentages in the square brackets indicate which share of the total sample of natives’ and migrants’ descendants is included in the analysis. The remaining share is excluded because the parents of these respondents are in the lowest education category. The variable group with the largest contribution to the explained part of mobility gap in each country is highlighted in bold.

**Table 6.** Decomposition results for upward mobility conditioned on parental education.

	Belgium	Switzerland	Czech Republic	Estonia	France	Croatia	Luxembourg	Latvia
Probability of upward mobility conditioned on parental education								
Migrants	75.248 ***	92.913 ***	76.543 ***	82.278 ***	82.647 ***	70.073 ***	71.250 ***	83.529 ***
	(0.000)	(0.000)	(0.000)	(0.000)	(0.000)	(0.000)	(0.000)	(0.000)
	69%	38%	77%	22%	76%	58%	67%	34%
Natives	71.196 ***	83.810 ***	89.965 ***	83.612 ***	80.302 ***	68.255 ***	57.996 ***	79.787 ***
	(0.000)	(0.000)	(0.000)	(0.000)	(0.000)	(0.000)	(0.000)	(0.000)
	45%	15%	56%	27%	72%	53%	39%	35%
Mobility gap	4.052	9.104 ***	−13.422 ***	−1.334	2.345	1.818	13.254 ***	3.742
	(0.381)	(0.008)	(0.000)	(0.760)	(0.290)	(0.657)	(0.000)	(0.249)
Explained	−0.662	4.919 ***	−6.129 ***	0.379	1.144 **	1.072	7.038 ***	1.650 ***
	(0.532)	(0.004)	(0.000)	(0.548)	(0.012)	(0.119)	(0.000)	(0.004)
Unexplained	4.714	4.185	−7.293 *	−1.713	1.200	0.746	6.216 *	2.093
	(0.200)	(0.121)	(0.057)	(0.580)	(0.551)	(0.833)	(0.073)	(0.499)
Contribution of covariates to differences in characteristics (explained part)								
Birth cohort	3.256 ***	2.265	−1.524 ***	−0.047	2.951 ***	1.328 ***	6.288 ***	2.159 ***
	**(0.000)**	(0.187)	(0.000)	(0.860)	**(0.000)**	**(0.005)**	**(0.000)**	**(0.000)**
Gender	−0.024	0.434 ***	−0.349 ***	0.119 **	0.229 ***	−0.126 ***	0.885 ***	−0.568 ***
	(0.605)	(0.000)	(0.000)	(0.034)	(0.000)	(0.000)	(0.000)	(0.000)
Parents	−1.344 *	−0.082	−0.661 **	−0.401	−0.047	1.114 ***	−1.137 **	0.455
	(0.056)	(0.900)	(0.041)	(0.384)	(0.876)	(0.001)	(0.040)	(0.251)
Household	−0.653 **	2.337 ***	−3.489 ***	0.250	−1.805 ***	−0.379	0.232	−0.081
	(0.041)	**(0.010)**	**(0.000)**	(0.515)	(0.000)	(0.258)	(0.525)	(0.579)
Finance	−1.897 ***	−0.035	−0.106	0.459 *	−0.188 ***	−0.864 ***	0.766 ***	−0.325 *
	(0.000)	(0.918)	(0.215)	**(0.063)**	(0.000)	(0.000)	(0.002)	(0.086)
*N*	1882	757	3509	915	5833	2446	1328	1486
*N* natives	1781	630	3428	836	5493	2309	1088	1316
*N* migrants	101	127	81	79	340	137	240	170

This table shows the percentage of upwardly mobile migrants’ and natives’ descendants and the contribution of characteristics to the difference between the groups. The sample is restricted to individuals whose more highly educated parent achieved an education level corresponding to ISCED 0–2. Migrants (natives) refers to the upward mobility of migrants’ (natives’) descendants. Explained (unexplained) refers to the part of the mobility gap arising from differences in characteristics (parameters). The *p*-value based on Equation (3) is shown in parentheses. ***, **, * signify significance of the effects at the 1%, 5%, 10% level, respectively, based on Equation (4). The percentages in the square brackets indicate which share of the total sample of natives’ and migrants’ descendants is included in the analysis. The remaining share is excluded because the highest educated parent of these respondents is not in the ISCED 0–2 category. The variable group with the largest contribution to the explained part of mobility gap in each country is highlighted in bold.

**Table 7.** Decomposition results for upward mobility of two education levels conditioned on parental education.

	Belgium	Switzerland	Germany	Estonia	France	Luxembourg	Latvia	UK
Probability of upward mobility								
Migrants	22.772 ***	17.323 ***	31.481 ***	20.253 ***	28.824 ***	15.417 ***	14.118 ***	46.512 ***
	(0.000)	(0.000)	(0.000)	(0.000)	(0.000)	(0.000)	(0.000)	(0.000)
	69%	38%	77%	22%	76%	58%	67%	34%
Natives	25.660 ***	13.333 ***	26.972 ***	19.856 ***	25.196 ***	11.673 ***	15.805 ***	29.926 ***
	(0.000)	(0.000)	(0.000)	(0.000)	(0.000)	(0.000)	(0.000)	(0.000)
	45%	15%	56%	27%	72%	53%	39%	35%
Mobility gap	−2.887	3.990	4.509	0.397	3.628	3.744	−1.688	16.586 ***
	(0.517)	(0.238)	(0.479)	(0.933)	(0.136)	(0.111)	(0.569)	(0.001)
Explained	−2.379 **	4.528 **	4.860 **	1.227 **	−1.225 ***	5.870 ***	−0.441	0.028
	(0.020)	(0.042)	(0.013)	(0.012)	(0.006)	(0.000)	(0.223)	(0.971)
Unexplained	−0.508	−0.539	−0.351	−0.830	4.853 **	−2.126	−1.246	16.557 ***
	(0.889)	(0.894)	(0.950)	(0.729)	(0.039)	(0.401)	(0.627)	(0.001)
Contribution of covariates to differences in characteristics (explained part)								
Birth cohort	0.013	0.473	1.488	0.179	1.835 ***	5.629 ***	0.050	0.712 ***
	(0.985)	(0.791)	(0.365)	(0.244)	(0.000)	**(0.000)**	(0.768)	(0.007)
Gender	−0.085	0.332 ***	0.622 ***	−0.017	−0.153 **	0.241	−0.429 ***	−0.112
	(0.106)	(0.004)	(0.000)	(0.682)	(0.023)	(0.105)	**(0.000)**	(0.124)
Parents	−0.936	0.609	2.920 ***	0.283	−0.137	−0.836 *	0.251	1.087 **
	(0.170)	(0.476)	**(0.008)**	(0.428)	(0.651)	(0.077)	(0.446)	(0.012)
Household	−1.016 ***	2.444 **	0.412	0.586 ***	−2.613 ***	0.587 **	−0.114 *	−1.681 ***
	**(0.004)**	**(0.012)**	(0.233)	**(0.007)**	**(0.000)**	(0.017)	(0.081)	**(0.002)**
Finance	−0.354	0.671	−0.582	0.197	−0.158 ***	0.240	−0.202 *	0.023 *
	(0.424)	(0.102)	(0.110)	(0.237)	(0.000)	(0.170)	(0.082)	(0.084)
*N*	1882	757	599	915	5833	1328	1486	2375
*N* natives	1781	630	545	836	5493	1088	1316	2289
*N* migrants	101	127	54	79	340	240	170	86

This table shows the percentage of migrants’ and natives’ descendants who surpass their more highly educated parent by two education classes and the contribution of characteristics to the difference between the groups. The sample is restricted to individuals whose more highly educated parent achieved an education level corresponding to ISCED 0–2. Migrants (natives) refers to the upward mobility of migrants’ (natives’) descendants. Explained (unexplained) refers to the part of the mobility gap arising from differences in characteristics (parameters). The *p*-value based on Equation (3) is shown in parentheses. ***, **, * signify significance of the effects at the 1%, 5%, 10% level, respectively, based on Equation (4). The percentages in the square brackets indicate which share of the total sample of natives’ and migrants’ descendants is included in the analysis. The remaining share is excluded because the highest educated parent of these respondents is not in the ISCED 0–2 category. The variable group with the largest contribution to the explained part of mobility gap in each country is highlighted in bold.

The results of the decomposition indicate that the greater probability of migrants’ children of reaching a higher education class than their parents is statistically significant in seven of 11 countries (Belgium, Switzerland, Germany, France, Croatia, Luxembourg and the United Kingdom). Only in Latvia, migrants’ descendants are significantly less likely to be upwardly mobile than their native counterparts. The contribution of the difference in observable characteristics between immigrants’ and natives’ children (the explained part) is highly statistically significant in all of these countries, while the contribution of differences in returns to characteristics (the unexplained part) exhibits much lower significance levels or is even statistically insignificant (with the exception of Latvia and the United Kingdom, where significance levels are higher than 5%).^18^ ^18^The results reported in the table should be interpreted as in the following examples. In Belgium, migrants’ children are 9.4 percentage points more likely than natives’ children to surpass their parents’ education level. Differences in characteristics between natives and second-generation migrants explain 3 percentage points (about 40%) of this mobility gap. Due to unobserved factors, which explain the remaining part of the gap, migrants’ children have a 6.3 percentage points higher probability of being upwardly mobile. In Latvia, by contrast, migrants’ children are 5.7 percentage points less likely than their native peers to surpass their parents in terms of education. While differences in characteristics between migrants’ and natives’ children account for a 1.2 percentage point lower probability of upward mobility, it is unobserved factors that drive the bigger part of the lower mobility of second-generation migrants (4.5 percentage points or about 80% of the mobility gap). The same interpretation applies to the results for individual variables or variable groups.


In the majority of countries, the difference in mobility between immigrants’ and natives’ children can in large part be explained by the difference in parental education between the groups. Whenever migrants are more often (less often) upwardly mobile, their parents’ education significantly contributes to this higher (lower) mobility. To analyse these patterns in more detail, we can compare them with the results of the logit regressions for natives (which form the base group for the decomposition), reported in Table B1 in Appendix B.^19^ ^19^The results for second-generation migrants are reported in Table B2 in Appendix B. Greater education for the mother and the father is related to higher upward mobility of their offspring (see the positive effects of the mother’s and the father’s education). A higher threshold set by the education level of the most highly educated parent, however, decreases the probability of a person being upwardly mobile (see the negative effects of the highest parental education). Quantitatively the negative effect of the education threshold is larger than the positive effect of parental education and, thus, it outweighs the latter. Because in most countries in our sample (except for the Baltic states) migrant parents are on average less highly educated than their native counterparts, the lower threshold that this implies for upward mobility is an important contributor to the higher mobility of migrants’ children as compared to those of natives in these countries, which is in line with other literature (Van Ours and Veenman 2003). For the Baltic states, in which native parents have on average lower education levels than migrants, the opposite applies (see descriptive statistics in Table A2 in Appendix A). In most countries in our sample, parental education is also quantitatively the largest contributor to the mobility gap between natives’ and migrants’ children.

Household characteristics make the biggest difference in the probability of upward mobility for migrants’ and natives’ descendants in Estonia and France. Their effect is statistically significant also for most other countries in our sample. In particular, living in relatively large households at age 14 (especially with many other children) shrinks the higher probability of a person being upwardly mobile. The larger household sizes of migrant households as compared to natives in Belgium, the Czech Republic, France, Croatia and the United Kingdom make it more difficult for migrants’ descendants to be upwardly mobile (as in, for example, Lindahl 2008). The opposite is true in Austria, Switzerland and the Baltic countries, where in our sample household sizes of migrants are on average smaller than those of natives, which is related to greater mobility for the migrants.

In Latvia, it is differences in the gender composition between the groups that plays the biggest role in explaining the mobility gap observed in the data. In this country (like in Estonia and Belgium), women are more likely than men to have reached a higher education level than their parents. The lower proportion of women among second-generation migrants in comparison to the sample of native respondents contributes to the overall lower mobility of second-generation migrants in those countries. In all other countries in our sample, male respondents are more likely to be upwardly mobile. Thus, the higher proportion of men (women) among natives’ descendants in Austria, Switzerland, Czech Republic, Germany and Croatia (France and Luxembourg) explains the observed mobility patterns.

The birth cohort of the respondents and their age contributes to a higher upward mobility of migrants’ children in Belgium, France, Luxembourg, the United Kingdom and the Baltic countries, and to lower mobility in the Czech Republic. In the Czech Republic, a higher probability of being upwardly mobile of younger persons, together with the on average older age of second-generation migrants makes it more difficult for them to be upwardly mobile. By contrast, in Belgium, France, Luxembourg and the United Kingdom, migrants’ children are on average younger than their native peers (they are born in later birth-cohorts). The higher mobility of younger individuals in those countries adds to the higher probability of being upwardly mobile for migrants’ descendants.^20^ ^20^More specifically, it is the younger age of second-generation migrants in France and the United Kingdom that explains their higher mobility, while in Belgium cohort effects have a larger impact. In Luxembourg, it is a combination of both effects. In the Baltic countries, individuals who are born in the 50s and 60s are more likely to be upwardly mobile than persons born in later cohorts. Thus, the on average older age of migrants’ descendants in those countries explains the positive contribution to them being upwardly mobile.

Parental characteristics contribute to the higher mobility of migrants’ descendants positively in Germany, Croatia and Latvia, and negatively in Belgium, the Czech Republic and Luxembourg. Which characteristics drive this pattern in each specific case can be analysed in more detail by consulting the results of the detailed decomposition reported in Table B3 in Appendix B. Often the mother’s age, her labour market status and the parental age difference are important drivers of the negative effects, while for the positive effects a large part stems from the parental age difference and the father’s age. Finally, the financial situation of the household usually remains an insignificant contributor to the mobility gap (with the exception of the United Kingdom, where it contributes to a higher upward mobility of second-generation migrants).

Turning to the unexplained part of the mobility differences, we see that this part is statistically significant (although only at the 10% level) and positively relates to higher mobility for migrants in five countries in our sample (Belgium, Switzerland, France, Luxembourg and the United Kingdom). Chiswick (1978) and Borjas (1994) raise the idea that persons who are more motivated to succeed might select themselves into migration. Such selection, and the higher motivation of persons with a migration background that it implies, would be reflected in a positive unexplained component of the mobility gap. Only in Latvia is the unexplained part statistically significant and negative, which could be driven by return migration of more able second-generation migrants.^21^ ^21^Hazans, Trapeznikova, and Rastrigina (2008) point out that between 1991 and 2002, 17% of the minority population in Latvia left the country. If ability selection lead to the emigration of more able persons, as suggested by Chiswick (1978) and Borjas (1994), this might explain the negative unexplained part for Latvia. An alternative explanation might be the transition from central planning to a market economy after the dissolution of the Soviet Union and its resulting effects on the education system. During the transition, the Latvian education system stopped offering courses in Russian for the large proportion of Russian-speaking minorities (42% in 2002) to replacing them with courses in the national language. At the same time, returns to education increased and access constraints to higher education were removed, resulting in higher demand for tertiary education among the native population. The two effects together have potentially contributed to a falling ratio of tertiary graduation rates between the minority and native population (see Hazans, Trapeznikova, and Rastrigina 2008, for details). We leave an analysis of which of the two potential explanations account for the observed pattern in Latvia to future research.


All in all, parental education is the most influential factor in explaining mobility differences between natives’ and migrants’ children in most countries. Whenever migrants’ children are upwardly mobile more often than natives’, it is in large part because their parents started out with relatively low education levels. The literature shows that children’s success in school depends largely on their parents’ education levels, which are in turn related to the levels of human, social and financial capitals from which the children can benefit (Haveman and Wolfe 1995). Thus, although empirical studies often find that migrants’ academic success is lower than natives’ (Riphahn 2003; Colding 2006; Schneeweis 2011), we show that the children of migrants in the last generation, who grew up in homes with fewer resources than their native peers in many countries, were still able to narrow the education gap with natives’ children.

#### Downward mobility

In order to draw a more comprehensive picture of whether the patterns found so far imply that the education gap between natives and second-generation migrants is narrowing, we repeat our analysis from above focusing on the probability of migrants’ and natives’ descendants reaching a lower education level than their more highly educated parent. If migrants’ children are more likely to be downwardly mobile, the implication of a narrowing gap based on the fact that they are more likely to be upwardly mobile at the same time (as shown in section ‘Upward mobility’) would be nullified. Table 5 presents the results of the Oaxaca–Blinder decomposition focusing on educational downward mobility.^22^ ^22^Because of the small number of downwardly mobile second-generation migrants in Belgium, the Czech Republic and the United Kingdom (3, 1 and 9, respectively) we had to exclude these countries from this analysis. Descriptive statistics for this subsample (and also for the following analyses) and a summary of the results from the logit regressions can be found in the online appendix.


In Germany, France and Luxembourg, migrants’ descendants are significantly less likely to be downwardly mobile than their native peers, while in Estonia the opposite applies. Differences in observable characteristics between the children of natives and migrants are significant contributors to the mobility gap in all of these countries. Additionally to this explained part of the mobility gap, in Germany and Estonia also the unexplained part of the decomposition is statistically significant.

Concerning the contribution of different variable groups to the mobility gap explained by observable characteristics, we find that, as in the analysis of upward mobility, parental education is the largest contributor to this difference in most countries. In all countries in which the explained difference is negative (positive), the gap is driven in large part by differences in parental education between natives’ and migrants’ children. The more detailed decomposition results in Table B4 in Appendix B show that this is again driven by the threshold effect of education, which suggests that the on average lower education level of migrant parents in most countries makes it less likely for their children to reach even lower education levels. For Switzerland and Latvia, the birth cohort of the respondent turns out to have the quantitatively largest impact on the explained part of the mobility difference. However, in these countries the overall difference in downward mobility between migrants’ children and their native peers is statistically insignificant.

#### Upward mobility conditioned on parental education

Our results thus far suggest that in most countries in which we detect a statistically significant difference in mobility, migrants’ children have an advantage: they are more often upwardly mobile and less often downwardly mobile than their native peers. The only exceptions are the Baltic countries where migrants are on average more highly educated than natives. We also uncovered that an important factor for explaining these mobility differences is the threshold implied by the education level of the more highly educated parent. This raises the question: are second-generation migrants upwardly mobile more often only because they have a lower threshold to surpass, or would they be more mobile also if their parents were equally educated as natives?

In order to perform this analysis, we restrict our sample to respondents whose more highly educated parents have the same highest education level (ISCED 0–2) and repeat the analysis from before on the restricted sample. In this way we compare mobility patterns of natives’ and migrants’ children that face the same education threshold to surpass.^23^ ^23^We had to exclude Austria, Germany and the United Kingdom from the analysis because of the small sample size for migrants (45 in Austria) or small number of migrants’ children who are not upwardly mobile (four and three cases in Germany and the United Kingdom, respectively). We exclude all parental education variables and its interaction with the financial situation from the regressions because of the multicollinearity induced by conditioning on the same parental education level. The results, which are reported in Table 6, suggest that once we restrict our sample to observations with the same parental education, migrants’ children are significantly less often upwardly mobile in the Czech Republic, and significantly more often upwardly mobile in Switzerland and Luxembourg. In all three countries, differences in observable characteristics between the groups are statistically significant contributors to the overall mobility gap. In the Czech Republic and Luxembourg also unexplained factors are marginally statistically significant.

Turning to the contribution of the different variable groups to the mobility gap explained by differences in observable characteristics, birth cohorts account for the largest part in the majority of countries. In Switzerland and the Czech Republic, it is household size (especially the number of children in a household, see detailed results in Table B5 in Appendix B) that contribute the largest part to the mobility gaps, and in Estonia it is differences in the financial situation of households when the respondent was 14 years old.

#### Two classes upward mobility conditioned on parental education

Finally, we shortly compare the probability of migrants’ and natives’ descendants of surpassing their more highly educated parent by two education classes. The idea behind this analysis is that if migrants are also more likely to jump two classes, they are even more likely to narrow the education gap with natives.^24^ ^24^Here too we restrict our sample to persons whose more highly educated parent reached an education level corresponding to ISCED 0–2. Austria, the Czech Republic and Croatia had to be excluded from the analysis because of the small number of migrants’ descendants who surpass their more highly educated parent by two education levels (1, 7 and 8, respectively).
Table 7 shows that in the United Kingdom, migrants’ children are significantly more likely than natives’ to surpass their more highly educated parent by two education classes.^25^ ^25^The results of the detailed decomposition are reported in Table B6 in Appendix B. This mobility difference cannot be explained by observable characteristics but is driven by unobservable factors. In all other countries in our sample, the mobility gap between natives’ and migrants’ descendants is statistically insignificant. However, there is some indication that in Belgium and France observable characteristics (mainly larger household sizes) would explain a lower (two-class) upward mobility of second-generation migrants, while in Switzerland, Germany, Estonia and Luxembourg the opposite is true (mainly explained by household size in Switzerland and Estonia, parental characteristics in Germany and birth cohorts in Luxembourg).

All in all, our results suggest that the higher educational upward mobility of second-generation migrants that has been found in many European countries can be, to a large extent, explained by the lower threshold implied by the on average lower education level of their parents (as in Belgium, Switzerland, Germany, France, Croatia, Luxembourg and the United Kingdom). Parental education also explains the lower downward mobility of migrants’ children that is detected in Germany, France and Luxembourg. Once we restrict our sample to persons that face a similar education threshold, the significant difference in mobility patterns disappears in many countries. Second-generation migrants are then more often upwardly mobile only in Switzerland and Luxembourg, and surpass their parents by two education levels more often than natives only in the United Kingdom. On the other hand, we detect a higher upward mobility of natives in Latvia and a lower downward mobility of natives in Estonia, countries in which migrant parents are on average more highly educated than native parents. Thus, a similar catch-up process of natives seems to take place in those countries. Once we restrict our sample to individuals with similar parental education, a higher upward mobility of natives is only detected in the Czech Republic.

Although data limitations impede the analysis of the patterns found here in further detail, this would be a promising direction for future research, which could focus on one specific country and dig deeper into what drives differences in educational mobility after accounting for parental education. In any case, the results here challenge the idea of more highly motivated migrants. Instead, the research shows that the often lower threshold that migrants’ children need to surpass in order to be upwardly mobile is the main driver of mobility differences to natives in most countries. Beyond the lower education of the parents, we find that some personal and household-level characteristics, in particular household size at age 14, also matters (though less so). Regardless of the reason, though, the education gap appears to have been closing in most countries across the last two generations.

## IV. Conclusion and discussion

The analysis in this article provides clear evidence of a narrowing gap in educational attainment levels between natives and immigrants across the two most recent generations. Migrant parents are less educated than native parents in many European countries, but their children are often able to surpass their parents’ education levels and get closer to the level of education achieved by their native peers. In most countries, the socioeconomic background of native and migrant parents is an important determinant of the mobility differences between their children. Apart from parental education, which is the quantitatively most important contributor to the mobility difference in most countries, the size of the household when the respondent was 14 turns out to be an important driver of differences in upward mobility between the two groups in most of the countries in our sample, while the financial situation of the household at that time is more important for explaining patterns of downward mobility. In many countries, the age structure and the gender composition of the two groups also contributes to the observed mobility differences, while parental characteristics are statistically insignificant drivers of mobility gaps in about half of the countries analysed.

In order to see the extent to which second-generation migrants’ greater educational mobility is driven by the lower threshold that they have to pass (because of the on average lower educational attainment levels of their parents in many countries), we provide additional information on the mobility difference between the children of similarly educated natives and migrants. Evidence points towards a higher educational upward mobility of migrants’ children in Switzerland and Luxembourg, whereas their mobility is significantly lower than that of their native peers in the Czech Republic.

The analysis presented in this article suggests that in most countries in our sample, the difference in educational attainment between natives and second-generation migrants has been narrowing across the two most recent generations. If this process persists over future generations, people with a migration background might soon have comparable education levels to the native population.

The findings in this article have important implications for the design of immigration policy, which often focuses on the on-average lower education level of immigrants into western European countries. Empirical studies often find a higher use of social welfare assistance of lower educated individuals (see Barrett and McCarthy 2007; Blume and Verner 2007; Boeri 2010; Pellizzari 2013; Huber and Oberdabernig 2016), which spurs arguments against the inflow of unskilled immigrants. Although the effects of education on welfare receipt are usually small and many studies find an only small fiscal impact of immigration as a share of gross domestic product in most countries, unskilled immigration remains a concern (see OECD 2013, and studies cited therein). Reinforcing the argument that descendants of immigrants are likely to provide a strong positive fiscal contribution to the state budget (see Lee and Miller 1997; cited by OECD 2013), our study shows that if the patterns detected for the last two generations continue, the potentially negative effect of migrants’ lower education on their net fiscal position eventually dies out in the long run, which would lead to similar conclusions.

Further, to the extent to which policy makers want to speed up the convergence of migrants’ to natives’ education levels over generations, they could consider policies which have been shown to reduce intergenerational persistence in educational outcomes, such as later tracking, early school entry and greater access to pre-school and kindergarten programmes (Bauer and Riphahn 2006a; Schütz, Ursprung, and Wößmann 2008; Bauer and Riphahn 2009a). These policies could be particularly helpful to migrants’ descendants, given that their parents are often less educated than native-born parents.

## Supplementary Material

intergenerational_persistence_migrants_appendix_tables.pdf

## Appendices

### Appendix A. Data appendix

#### Descriptive statistics

**Table A1.** Descriptive statistics of all variables for the full sample.

	AT	BE	CH	CZ	DE	EE	FR	HR	LU	LV	UK
	Natives’ children										
Birth year	1966	1967	1966	1967	1965	1968	1967	1966	1966	1968	1966
Cohort 50s	0.26	0.27	0.30	0.31	0.30	0.25	0.28	0.34	0.33	0.25	0.28
Cohort 60s	0.39	0.31	0.37	0.27	0.38	0.33	0.31	0.31	0.32	0.31	0.34
Male (d)	0.48	0.49	0.46	0.43	0.47	0.49	0.47	0.50	0.49	0.45	0.45
Father’s age at birth	30.32	30.08	31.58	28.19	29.63	30.12	29.60	29.53	31.11	29.68	30.10
Mother’s age at birth	26.93	27.60	28.39	25.00	26.85	27.40	26.96	26.01	27.78	27.36	27.32
Age diff. of parents	4.22	3.20	4.05	3.62	3.56	4.09	3.50	4.22	4.12	3.82	3.56
Mother out of labour f. (d)	0.44	0.50	0.47	0.08	0.44	0.07	0.42	0.53	0.63	0.08	0.26
Mothers’ education	1.46	1.56	1.59	1.34	1.82	1.82	1.27	1.29	1.38	1.71	1.36
Fathers’ education	1.77	1.67	1.93	1.42	2.20	1.73	1.31	1.51	1.64	1.61	1.56
Highest parental educ.	1.83	1.82	2.00	1.54	2.26	1.98	1.43	1.55	1.71	1.83	1.69
# of adults in hh	2.71	2.34	2.50	2.16	2.26	2.27	2.40	2.60	2.58	2.16	2.28
# of children in hh	2.51	2.52	2.51	2.21	2.19	2.28	1.58	2.19	2.34	2.31	2.28
Financial situation	3.72	4.39	4.36	4.04	4.13	4.01	3.96	3.67	4.24	4.11	4.03
Birth year	1970	1971	1969	1962	1964	1967	1970	1968	1973	1966	1969
Cohort 50s	0.23	0.19	0.12	0.53	0.34	0.26	0.17	0.24	0.08	0.31	0.17
Cohort 60s	0.18	0.22	0.39	0.30	0.38	0.35	0.32	0.32	0.27	0.31	0.37
Male (d)	0.43	0.50	0.47	0.39	0.47	0.49	0.50	0.49	0.53	0.52	0.40
Father’s age at birth	31.75	31.82	31.19	28.76	31.28	29.08	32.42	30.50	30.94	29.88	31.95
Mother’s age at birth	28.10	27.42	27.75	26.29	28.07	27.68	27.62	27.26	27.41	27.95	28.65
Age diff. of parents	4.48	5.13	4.28	3.66	3.96	3.18	5.26	4.05	4.42	3.28	4.45
Mother out of labour f. (d)	0.34	0.68	0.33	0.14	0.46	0.05	0.57	0.62	0.51	0.05	0.28
Mothers’ education	1.32	0.98	1.27	1.10	1.52	1.93	0.93	1.20	1.23	1.65	1.36
Fathers’ education	1.63	1.08	1.60	1.10	1.91	1.90	1.04	1.45	1.32	1.69	1.41
Highest parental educ.	1.74	1.17	1.67	1.17	1.96	2.11	1.12	1.48	1.40	1.84	1.58
# of adults in hh	2.64	2.52	2.34	2.31	2.36	2.25	2.90	2.83	2.42	2.13	2.71
# of children in hh	2.28	3.02	1.98	3.04	2.11	1.89	2.24	2.45	2.47	2.22	2.85
Financial situation	3.91	3.85	4.28	3.80	3.95	4.04	3.75	3.54	4.14	4.12	4.04

Age difference refers to the absolute age difference between the parents. Mother out of labour force is an indicator for whether the respondents’ mother was in or out of the labour force when the respondent was 14. # of persons in the household refers to the number of adults and children, respectively, in the household in which the respondents lived when they were 14 years old. Financial situation refers to the financial situation of the household in which the respondents lived when they were 14 years old. A detailed definition of variables can be found in the variable Table A3. Country-codes: AT = Austria, BE = Belgium, CH = Switzerland, CZ = Czech Republic, DE = Germany, EE = Estonia, FR = France, HR = Croatia, LU = Luxembourg, LV = Latvia, UK = United Kingdom.

**Table A2.** Descriptive statistics of all variables for the sample used for upward mobility.

	AT	BE	CH	CZ	DE	EE	FR	HR	LU	LV	UK
Natives’ children											
Birth year	1966	1966	1965	1966	1965	1966	1966	1965	1965	1967	1966
Cohort 50s (d)	0.27	0.31	0.32	0.32	0.33	0.29	0.31	0.35	0.35	0.28	0.30
Cohort 60s (d)	0.39	0.32	0.38	0.27	0.38	0.35	0.33	0.32	0.32	0.32	0.35
Male (d)	0.48	0.49	0.46	0.43	0.47	0.49	0.46	0.51	0.49	0.45	0.45
Fathers’ age at birth	30.36	29.98	31.56	28.14	29.51	30.59	29.62	29.47	31.04	29.76	29.98
Mothers’ age at birth	26.96	27.49	28.31	24.94	26.71	27.65	26.87	26.00	27.67	27.37	27.14
Age diff. of parents	4.26	3.23	4.11	3.63	3.58	4.34	3.59	4.19	4.17	3.91	3.62
Mother out of labour f. (d)	0.44	0.54	0.46	0.08	0.48	0.08	0.44	0.56	0.64	0.08	0.26
Mothers’ education	1.36	1.24	1.50	1.24	1.65	1.52	1.07	1.19	1.29	1.48	1.09
Fathers’ education	1.56	1.28	1.75	1.25	1.86	1.48	1.08	1.40	1.51	1.41	1.28
Highest parental educ.	1.61	1.38	1.82	1.37	1.90	1.65	1.14	1.42	1.56	1.56	1.33
# of adults in hh	2.76	2.38	2.54	2.17	2.30	2.29	2.44	2.63	2.61	2.17	2.30
# of children in hh	2.54	2.53	2.53	2.24	2.22	2.35	1.62	2.22	2.35	2.37	2.29
Financial situation	3.63	4.25	4.25	3.99	3.95	3.93	3.86	3.59	4.16	4.04	3.93
*Migrants’ children*											
Birth year	1971	1971	1969	1961	1965	1966	1970	1968	1972	1966	1968
Cohort 50s (d)	0.22	0.20	0.12	0.54	0.33	0.29	0.17	0.23	0.08	0.33	0.18
Cohort 60s (d)	0.16	0.22	0.38	0.30	0.40	0.36	0.32	0.34	0.28	0.32	0.41
Male (d)	0.44	0.51	0.46	0.39	0.46	0.52	0.51	0.49	0.52	0.52	0.40
Fathers’ age at birth	30.87	31.94	31.13	28.71	31.35	29.05	32.48	30.55	30.72	29.81	31.77
Mothers’ age at birth	27.74	27.51	27.63	26.26	28.03	27.59	27.54	27.40	27.36	28.00	28.43
Age diff of parents	4.15	5.21	4.37	3.65	4.10	3.32	5.37	4.00	4.32	3.22	4.53
Mother out of labour f. (d)	0.34	0.69	0.31	0.15	0.50	0.06	0.58	0.66	0.50	0.05	0.33
Mothers’ education	1.16	0.91	1.09	1.07	1.39	1.56	0.84	1.12	1.12	1.46	0.99
Fathers’ education	1.34	1.01	1.35	1.07	1.61	1.53	0.92	1.34	1.20	1.46	1.16
Highest parental educ.	1.46	1.08	1.42	1.14	1.65	1.67	0.99	1.37	1.27	1.58	1.19
# of adults in hh	2.66	2.51	2.40	2.32	2.38	2.28	2.95	2.89	2.42	2.15	2.72
# of children in hh	2.31	3.06	1.95	3.06	2.20	1.95	2.27	2.43	2.51	2.32	2.89
Financial situation	3.72	3.82	4.21	3.78	3.90	3.95	3.71	3.49	4.07	4.06	3.88

This table reports descriptive statistics for the sample used for investigating differences in upward mobility, that is, after excluding respondents whose more highly educated parent had already reached the highest education level. Age difference refers to the absolute age difference between the parents. Mother out of labour force is an indicator for whether the respondents’ mother was in or out of the labour force when the respondent was 14. # of persons in the household refers to the number of adults and children, respectively, in the household in which the respondents lived when they were 14 years old. Financial situation refers to the financial situation of the household in which the respondents lived when they were 14 years old. A detailed definition of variables can be found in the variable Table A3. Country-codes: AT = Austria, BE = Belgium, CH = Switzerland, CZ = Czech Republic, DE = Germany, EE = Estonia, FR = France, HR = Croatia, LU = Luxembourg, LV = Latvia, UK = United Kingdom.

#### Variable definitions

**Table A3.** Variable definitions.

Variable	Variable definition
Upward mobility	Dummy variable equal to one if the respondent reaches a higher education class than the highest educated parent.
Downward mobility	Dummy variable equal to one if the respondent reaches a lower education class than the highest educated parent.
Birth year	Birth year of the respondent.
Cohort 50s (d)	Dummy variable equal to one if the respondent is born between 1950 and 1959.
Cohort 60s (d)	Dummy variable equal to one if the respondent is born between 1960 and 1969.
Male (d)	Dummy variable equal to one for male respondents.
Father’ age at birth	Age of the father when the respondent was born.
Mothers’ age at birth	Age of the mother when the respondent was born.
Age diff of parents	Absolute value of the age difference between the parents.
Mother out of labour f. (d)	Dummy variable equal to one if the respondents’ mother was out of the labour force when the respondent was 14.
Mother’s education	Highest education level reached by the mother (0 ‘illiterate’, 1 ‘ISCED 0–2’, 2 ‘ISCED 3–4’; highest level 3 ‘ISCED 5–6’ excluded).
Fathers’ education	Highest education level reached by the father (0 ‘illiterate’, 1 ‘ISCED 0–2’, 2 ‘ISCED 3–4’; highest level 3 ‘ISCED 5–6’ excluded).
Highest parental educ.	Highest education level reached by the highest educated parent (0 ‘illiterate’, 1 ‘ISCED 0–2’, 2 ‘ISCED 3–4’; highest level 3 ‘ISCED 5–6’ excluded).
# of adults in hh	Number of adults living in the same household as the respondent when she/he was 14 years old.
# of children in hh	Number of children living in the same household as the respondent when she/he was 14 years old.
Financial situation	Financial situation of the household in which the respondent lived when she/he was 14 years old (ranging from 1 ‘very bad’ to 6 ‘very good’).

#### Dropped observations

**Table A4.** Sample sizes.

		Full sample in EU-SILC	Born in country of residence	Finished education	Parental information available	Resulting sample	Upward mobility	Upward mobility	Without ISCED 5–6 parental educ.	Downward mobility	Without illiterate parents	Conditional upward mobility	Max. ISCED 0–2 parental educ.
		*N*	Percentage dropped (%)	*N*	(%)	*N*	(%)	(%)	*N*	(%)	*N*	(%)	*N*
AT	Total	13,933	−29	−10	−44	5019	−5	−16	3990	0	4745		
	Nat					4905	−5	−16	3903	0	4639		
	Mig					114	−7	−18	87	0	106		
BE	Total	14,300	−33	−12	−47	4458	−8	−26	3051			−54	1882
	Nat					4300	−8	−27	2911			−55	1781
	Mig					158	−7	−5	140			−31	101
CH	Total	17,602	−39	−12	−47	4982	−11	−15	3736	0	4395	−83	757
	Nat					4604	−11	−15	3455	0	4078	−85	630
	Mig					378	−11	−16	281	−5	317	−62	127
CZ	Total	20,629	−18	−10	−59	6247	−1	−11	5518			−43	3509
	Nat					6142	−1	−11	5415			−44	3428
	Mig					105	0	−2	103			−23	81
DE	Total	28,644	−23	−9	−47	10,568	−23	−32	5514	0	8154		
	Nat					10,304	−23	−33	5361	0	7955		
	Mig					264	−25	−23	153	0	199		
EE	Total	13,426	−28	−16	−50	4071	−14	−25	2617	0	3505	−74	915
	Nat					3653	−14	−24	2378	0	3148	−73	836
	Mig					418	−15	−33	239	0	357	−78	79
FR	Total	27,071	−28	−9	−46	9520	−15	−15	6886	−1	8013	−28	5833
	Nat					8980	−15	−15	6466	0	7605	−28	5493
	Mig					540	−17	−7	420	−9	408	−24	340
HR	Total	16,948	−24	−9	−53	5507	−16	−8	4243	0	4609	−47	2446
	Nat					5225	−16	−8	4024	0	4374	−47	2309
	Mig					282	−16	−7	219	0	235	−42	137
LU	Total	14,891	−57	−14	−40	3317	−5	−10	2835	0	3142	−58	1328
	Nat					2938	−5	−10	2503	0	2784	−61	1088
	Mig					379	−5	−8	332	−1	358	−33	240
LV	Total	15,891	−28	−11	−55	4618	−8	−19	3468	0	4251	−65	1486
	Nat					4079	−8	−19	3059	0	3751	−65	1316
	Mig					539	−7	−18	409	0	500	−66	170
UK	Total	18,670	−29	−9	−56	5315	−14	−22	3582				
	Nat					5145	−14	−22	3466				
	Mig					170	−13	−22	116				

This table shows the share of observations dropped to obtain the final samples. We restrict the sample to persons born in their country of residence, with finished education. We have to drop observations with missing information on the respondents’ parents and household characteristics at the age of 14 (i.e. if information on parental country of birth or education is missing, if parents are coded as dead or unknown in EU-SILC or the respondent was living in a collective household or institution). From the resulting sample, we have to drop all individuals with missing information on the dependent or independent variables used in the empirical analysis. For the analysis of upward mobility, we exclude respondents whose more highly educated parent already reached the highest education level (ISCED 5–6). For the analysis of downward mobility, we exclude respondents whose parents are illiterate. For the analysis of upward mobility conditional on parental education we focus on respondents whose more highly educated parent has an education level corresponding to ISCED 0–2. Country-codes: AT = Austria, BE = Belgium, CH = Switzerland, CZ = Czech Republic, DE = Germany, EE = Estonia, FR = France, HR = Croatia, LU = Luxembourg, LV = Latvia, UK = United Kingdom.

**Table A5.** Mobility of dropped individuals due to missing values for explanatory variables.

	Migrants (%)	Natives (%)	Bias in mobility gap (%)	Migrants (%)	Natives (%)	Bias in mobility gap (%)	Migrants (%)	Natives (%)	Bias in mobility gap (%)	Migrants (%)	Natives (%)	Bias in mobility gap (%)
	Upward			Downward			Upward cond			Upward 2 classes		
AT	88	39	−4	0	12	1						
BE	78	49	−1				83	55	−2	0	14	0
CH	54	39	1	17	9	−1	75	83	2	10	15	1
CZ		78	0					93	0			
DE	39	36	3	20	13	−3				20	20	0
EE	50	43	−2	20	15	0	78	70	−2	4	17	3
FR	77	74	0	4	4	0	72	76	1	14	17	1
HR	50	52	1	5	5	0	56	65	2			
LU	56	32	0	6	19	0	83	48	−1	8	4	0
LV	48	49	0	10	13	0	83	73	−1	0	5	0
UK	68	70	3							31	18	0

This table shows the mobility of individuals that are dropped from the sample because of missing observation on any of the explanatory variables that are included in the analysis, grouped by migration background. Individuals that can per definition not be mobile in terms of education because of the parental education level are coded as missing and thus do not influence the numbers shown in the table. Natives refers to natives’ descendants, migrants refers to migrants’ descendants. The bias in mobility gap indicates how much larger or smaller the mobility gap detected in the data would be if those excluded observations were included in the multivariate analysis. Negative values for the bias in mobility gap indicate a downward bias, that is, the identified mobility gaps would be larger (or less negative) than the ones reported in the multivariate analysis (migrants’ children would be relatively more mobile than reported); positive values indicate an upward bias, that is, mobility gaps would be smaller (or more negative) than reported (i.e. migrants’ children would be relatively less mobile than reported). More detailed statistics are reported in the online appendix.

## Appendix B. Estimation results

**Table B1.** Logit result for upward mobility of natives’ children.

	Austria	Belgium	Switzerland	Czech Republic	Germany	Estonia	France	Croatia	Luxembourg	Latvia	UK
Birth year	−0.002	−0.001	−0.002	0.004 ***	−0.004 **	−0.004	0.005 ***	0.001	0.003	0.003	0.003 *
	(0.243)	(0.782)	(0.252)	(0.006)	(0.039)	(0.120)	(0.000)	(0.762)	(0.326)	(0.135)	(0.069)
Cohort 50s (d)	−0.080 **	−0.137 **	−0.086 *	0.021	−0.076 *	0.053	−0.041	−0.004	−0.082	0.211 ***	−0.037
	(0.037)	(0.015)	(0.055)	(0.541)	(0.052)	(0.353)	(0.238)	(0.931)	(0.172)	(0.000)	(0.412)
Cohort 60s (d)	−0.020	−0.015	−0.048	0.036 *	−0.040	0.063 *	0.001	0.039	−0.071 *	0.140 ***	−0.003
	(0.445)	(0.691)	(0.102)	(0.078)	(0.113)	(0.073)	(0.949)	(0.201)	(0.067)	(0.000)	(0.926)
Male (d)	0.083 ***	−0.045 ***	0.141 ***	0.033 ***	0.093 ***	−0.159 ***	0.021 **	0.061 ***	0.074 ***	−0.182 ***	0.001
	(0.000)	(0.007)	(0.000)	(0.000)	(0.000)	(0.000)	(0.023)	(0.000)	(0.000)	(0.000)	(0.915)
Mother’s age at birth	0.020 **	0.022	0.029 **	0.005	0.022 **	0.004	0.017 *	−0.001	0.038 **	0.016	0.027 **
	(0.041)	(0.106)	(0.022)	(0.566)	(0.049)	(0.723)	(0.053)	(0.920)	(0.020)	(0.147)	(0.011)
Mother’s age at b., sq (/100)	−0.038 **	−0.041 *	−0.041 **	−0.013	−0.043 **	−0.005	−0.033 **	−0.006	−0.060 **	−0.025	−0.040 **
	(0.023)	(0.061)	(0.040)	(0.432)	(0.018)	(0.775)	(0.027)	(0.764)	(0.029)	(0.158)	(0.018)
Father’s age at birth	0.007	0.021 *	0.020 **	0.001	0.014	0.004	0.004	0.022 ***	0.017	0.004	−0.007
	(0.352)	(0.054)	(0.049)	(0.876)	(0.101)	(0.612)	(0.590)	(0.010)	(0.176)	(0.660)	(0.399)
Father’s age at b., sq (/100)	−0.003	−0.022	−0.028 *	0.005	−0.005	−0.005	−0.000	−0.022 *	−0.017	−0.000	0.014
	(0.775)	(0.172)	(0.051)	(0.665)	(0.712)	(0.684)	(0.991)	(0.086)	(0.362)	(0.978)	(0.234)
Age difference parents	−0.003	−0.010 **	0.000	−0.002	−0.009 ***	−0.004	−0.004	−0.006 *	−0.003	−0.006 **	−0.005
	(0.228)	(0.016)	(0.889)	(0.573)	(0.007)	(0.235)	(0.103)	(0.053)	(0.462)	(0.023)	(0.148)
Mother out of labour f. (d)	0.002	−0.040 **	0.015	−0.064 ***	−0.004	0.016	−0.008	0.006	0.019	0.028	−0.012
	(0.865)	(0.033)	(0.320)	(0.000)	(0.755)	(0.653)	(0.456)	(0.690)	(0.325)	(0.331)	(0.425)
Mother’s education	0.089 ***	0.079 ***	0.066 ***	0.076 ***	0.066 ***	0.164 ***	0.067 ***	0.162 ***	0.123 ***	0.158 ***	0.017
	(0.000)	(0.010)	(0.000)	(0.000)	(0.000)	(0.000)	(0.004)	(0.000)	(0.000)	(0.000)	(0.438)
Father’s education	0.054 *	0.076 **	0.037	0.074 ***	0.010	0.064 ***	0.046 *	0.148 ***	0.100 **	0.106 ***	−0.006
	(0.078)	(0.020)	(0.176)	(0.000)	(0.735)	(0.009)	(0.050)	(0.002)	(0.023)	(0.000)	(0.847)
Highest parental educ.	−0.541 ***	−0.349 ***	−0.617 ***	−0.523 ***	−0.579 ***	−0.609 ***	−0.255 ***	−0.497 ***	−0.564 ***	−0.482 ***	−0.254 ***
	(0.000)	(0.000)	(0.000)	(0.000)	(0.000)	(0.000)	(0.000)	(0.000)	(0.000)	(0.000)	(0.000)
# of children in hh	−0.007	−0.016 ***	−0.012 **	−0.032 ***	−0.011 *	−0.016 **	−0.019 ***	−0.020 ***	−0.007	−0.026 ***	−0.021 ***
	(0.111)	(0.005)	(0.042)	(0.000)	(0.053)	(0.012)	(0.000)	(0.001)	(0.324)	(0.000)	(0.000)
# of adults in hh	−0.011 *	−0.017 **	−0.029 ***	−0.020 ***	−0.009	−0.023 *	−0.025 ***	0.003	−0.019 **	−0.026 **	−0.049 ***
	(0.053)	(0.049)	(0.000)	(0.007)	(0.171)	(0.058)	(0.000)	(0.575)	(0.023)	(0.047)	(0.000)
Financial situation	0.034 *	0.047 **	−0.004	0.019	−0.002	0.028	0.038 ***	0.080 ***	0.022	0.059 **	0.047 **
	(0.063)	(0.050)	(0.915)	(0.219)	(0.963)	(0.469)	(0.008)	(0.000)	(0.423)	(0.028)	(0.019)
Finance * highest educ.	−0.006	−0.005	0.004	−0.006	0.004	0.001	−0.017	−0.041 ***	0.014	−0.028 *	−0.025 *

***, ** and * signify significance of the effects at the 1%, 5% and 10% levels, respectively.

**Table B2.** Logit results for upward mobility of migrants’ children.

	Austria	Belgium	Switzerland	Czech Republic	Germany	Estonia	France	Croatia	Luxembourg	Latvia	UK
Birth year	−0.021 **	0.029 ***	−0.003	−0.016	0.006	0.017 **	−0.002	−0.007	0.002	−0.004	0.004
	(0.039)	(0.001)	(0.564)	(0.181)	(0.523)	(0.018)	(0.672)	(0.381)	(0.678)	(0.476)	(0.501)
Cohort 50s (d)	−0.275 *	0.300 ***	−0.039	−0.232	0.103	0.348 ***	−0.051	−0.136	−0.132	0.009	−0.013
	(0.089)	(0.000)	(0.739)	(0.220)	(0.600)	(0.006)	(0.684)	(0.449)	(0.369)	(0.941)	(0.924)
Cohort 60s (d)	0.015	0.214 **	−0.029	0.019	−0.123	0.269 ***	−0.027	0.055	0.007	0.032	−0.047
	(0.926)	(0.012)	(0.672)	(0.901)	(0.356)	(0.005)	(0.715)	(0.655)	(0.934)	(0.716)	(0.590)
Male (d)	0.130	−0.179 ***	0.124 ***	−0.033	0.011	−0.233 ***	0.040	0.031	0.092 **	−0.130 ***	−0.105 **
	(0.120)	(0.003)	(0.002)	(0.661)	(0.855)	(0.000)	(0.262)	(0.580)	(0.030)	(0.000)	(0.026)
Mother’s age at birth	−0.067	0.066	−0.037	−0.018	0.105 *	0.040	0.007	−0.024	0.095 ***	0.009	0.076 **
	(0.307)	(0.111)	(0.337)	(0.815)	(0.057)	(0.401)	(0.809)	(0.683)	(0.008)	(0.745)	(0.027)
Mother’s age at b., sq (/100)	0.060	−0.087	0.068	0.056	−0.135	−0.090	−0.027	0.035	−0.201 ***	−0.008	−0.118 **
	(0.562)	(0.178)	(0.300)	(0.688)	(0.101)	(0.269)	(0.542)	(0.729)	(0.001)	(0.845)	(0.017)
Father’s age at birth	0.170 ***	−0.015	0.030	0.049	0.012	0.057	0.028	0.085	−0.035	−0.034	−0.034
	(0.003)	(0.636)	(0.338)	(0.237)	(0.810)	(0.112)	(0.243)	(0.111)	(0.223)	(0.193)	(0.335)
Father’s age at b., sq (/100)	−0.171 **	0.001	−0.037	−0.078	−0.054	−0.082	−0.016	−0.102	0.096 **	0.054	0.035
	(0.038)	(0.980)	(0.442)	(0.197)	(0.432)	(0.154)	(0.611)	(0.184)	(0.037)	(0.155)	(0.406)
Age difference parents	−0.055 **	0.005	−0.013	0.009	0.001	−0.003	−0.012	−0.009	−0.010	−0.005	0.023 *
	(0.035)	(0.743)	(0.143)	(0.588)	(0.935)	(0.739)	(0.259)	(0.565)	(0.335)	(0.519)	(0.074)
Mother out of labour f. (d)	−0.070	−0.037	−0.028	−0.043	−0.079	−0.017	−0.096 ***	−0.045	0.025	0.035	−0.078
	(0.464)	(0.616)	(0.499)	(0.690)	(0.229)	(0.876)	(0.010)	(0.540)	(0.590)	(0.674)	(0.146)
Mother’s education	−0.022	0.124	0.079 **	−0.205	0.000	−0.074	0.089 *	0.176 **	0.106	0.221 ***	−0.001
	(0.851)	(0.146)	(0.031)	(0.359)	(0.997)	(0.323)	(0.057)	(0.046)	(0.250)	(0.005)	(0.983)
Father’s education	−0.150	0.236 **	−0.019	0.015	0.185	0.047	0.049	0.472 *	0.252 ***	0.066	0.100
	(0.319)	(0.042)	(0.773)	(0.955)	(0.226)	(0.563)	(0.472)	(0.082)	(0.010)	(0.280)	(0.403)
Highest parental educ.	−0.266	−1.436 ***	−0.624 ***	−0.120	−1.121 ***	0.118	−0.314 *	−0.713 **	−1.824 ***	−0.365 *	−0.331
	(0.406)	(0.001)	(0.001)	(0.802)	(0.007)	(0.700)	(0.053)	(0.023)	(0.000)	(0.080)	(0.103)
# of children in hh	0.031	−0.062 ***	−0.001	−0.022	−0.014	−0.081 ***	0.011	−0.002	−0.037 **	−0.006	0.019
	(0.434)	(0.000)	(0.975)	(0.262)	(0.637)	(0.004)	(0.244)	(0.928)	(0.026)	(0.681)	(0.489)
# of adults in hh	−0.060 *	−0.055 **	−0.087 ***	−0.082 **	−0.003	−0.035	−0.016	−0.043 **	0.001	−0.007	0.007
	(0.100)	(0.041)	(0.002)	(0.050)	(0.936)	(0.397)	(0.122)	(0.049)	(0.983)	(0.827)	(0.723)
Financial situation	0.027	−0.153	−0.033	0.020	−0.251	0.340 **	0.010	0.082	−0.302 ***	0.108	0.011
	(0.782)	(0.106)	(0.647)	(0.854)	(0.136)	(0.015)	(0.805)	(0.118)	(0.000)	(0.129)	(0.860)
Finance * highest educ.	−0.004	0.154 *	0.029	−0.046	0.092	−0.162 **	−0.001	−0.057	0.272 ***	−0.072	0.003

***, ** and * signify significance of the effects at the 1%, 5% and 10% levels, respectively.

**Table B3.** Detailed decomposition results for upward mobility.

	Austria	Belgium	Switzerland	Czech Republic	Germany	Estonia	France	Croatia	Luxembourg	Latvia	UK
Probability of upward mobility											
Migrants	47.126 ***	71.429 ***	61.566 ***	68.932 ***	53.595 ***	44.770 ***	82.143 ***	54.795 ***	58.735 ***	46.699 ***	90.517 ***
	(0.000)	(0.000)	(0.000)	(0.000)	(0.000)	(0.000)	(0.000)	(0.000)	(0.000)	(0.000)	(0.000)
Natives	44.427 ***	62.075 ***	37.540 ***	65.762 ***	39.619 ***	48.486 ***	77.127 ***	48.335 ***	40.272 ***	52.435 ***	71.754 ***
	(0.000)	(0.000)	(0.000)	(0.000)	(0.000)	(0.000)	(0.000)	(0.000)	(0.000)	(0.000)	(0.000)
Mobility gap	2.699	9.354 **	24.026 ***	3.170	13.975 ***	−3.716	5.016 **	6.460 *	18.463 ***	−5.736 **	18.763 ***
	(0.616)	(0.026)	(0.000)	(0.502)	(0.001)	(0.273)	(0.017)	(0.063)	(0.000)	(0.029)	(0.000)
Explained	7.684 ***	3.030 **	20.393 ***	8.968 ***	13.147 ***	0.065	1.827 ***	1.888 ***	14.102 ***	−1.201 ***	3.050 ***
	(0.000)	(0.011)	(0.000)	(0.000)	(0.000)	(0.873)	(0.001)	(0.000)	(0.000)	(0.000)	(0.000)
Unexplained	−4.985	6.323 *	3.633 *	−5.798	0.828	−3.782	3.190 *	4.571	4.361 *	−4.535 **	15.713 ***
	(0.168)	(0.053)	(0.081)	(0.108)	(0.776)	(0.109)	(0.063)	(0.130)	(0.087)	(0.025)	(0.000)
Contribution of covariates to differences in characteristics (explained part)											
Birth year	−1.150	−0.319	−1.089	−2.579 ***	0.048 *	0.144	1.752 ***	0.174	1.984	−0.472	0.691 *
	(0.245)	(0.778)	(0.252)	(0.006)	(0.051)	(0.124)	(0.000)	(0.763)	(0.326)	(0.136)	(0.069)
Cohort effects	1.122	1.585 *	1.707 *	0.731	−0.050	0.073	0.524	0.136	2.708	1.307 ***	0.393
	(0.244)	(0.093)	(0.062)	(0.484)	(0.215)	(0.126)	(0.217)	(0.813)	(0.159)	(0.000)	(0.321)
Gender	−0.446 ***	−0.065 **	−0.180 ***	−0.052 ***	−0.142 ***	−0.560 ***	0.086 **	−0.109 ***	0.277 ***	−1.543 ***	−0.008
	(0.000)	(0.011)	(0.000)	(0.009)	(0.000)	(0.000)	(0.024)	(0.000)	(0.000)	(0.000)	(0.912)
Mother’s age at birth	1.793 **	−0.049	−2.223 **	0.910	2.904 **	0.002	1.322 *	−0.163	−1.579 **	1.189	3.567 **
	(0.038)	(0.491)	(0.023)	(0.563)	(0.045)	(0.913)	(0.051)	(0.922)	(0.027)	(0.148)	(0.012)
Mother’s age at b., sq	−2.207 **	−0.478 *	1.923 **	−1.279	−3.269 **	0.046	−1.499 **	−0.472	0.795 **	−1.056	−3.193 **
	(0.020)	(0.066)	(0.040)	(0.429)	(0.015)	(0.800)	(0.026)	(0.761)	(0.048)	(0.159)	(0.019)
Father’s age at birth	0.411	4.166 *	−0.945 *	0.084	2.594	−0.752	1.053	2.617 ***	−0.734	0.054	−1.260
	(0.353)	(0.059)	(0.057)	(0.841)	(0.101)	(0.610)	(0.584)	(0.010)	(0.179)	(0.668)	(0.397)
Father’s age at b., sq	−0.140	−2.975	1.044 *	0.162	−0.525	0.644	−0.045	−1.477 *	0.376	−0.001	1.623
	(0.778)	(0.177)	(0.057)	(0.702)	(0.711)	(0.679)	(0.981)	(0.086)	(0.362)	(0.941)	(0.233)
Age difference parents	0.045	−1.913 **	0.014	0.017	−0.489 ***	0.421	−0.610	0.127 **	−0.057	0.423 **	−0.397
	(0.245)	(0.017)	(0.895)	(0.624)	(0.008)	(0.235)	(0.108)	(0.050)	(0.454)	(0.023)	(0.150)
Mother’s labour m. stat.	−0.023	−0.578 **	−0.228	−0.638 ***	−0.010	−0.032	−0.109	0.068	−0.267	−0.092	−0.085
	(0.860)	(0.033)	(0.320)	(0.000)	(0.738)	(0.682)	(0.457)	(0.692)	(0.325)	(0.331)	(0.426)
Mother’s education	−2.243 ***	−2.615 **	−2.688 ***	−1.969 ***	−1.765 ***	0.730 ***	−1.467 ***	−1.252 ***	−2.268 ***	−0.403 ***	−0.166
	(0.000)	(0.011)	(0.000)	(0.000)	(0.000)	(0.000)	(0.006)	(0.000)	(0.000)	(0.000)	(0.433)
Father’s education	−1.438 *	−1.989 **	−1.508	−2.046 ***	−0.253	0.373 ***	−0.621 *	−0.930 ***	−3.389 **	0.627 ***	0.086
	(0.076)	(0.023)	(0.178)	(0.000)	(0.735)	(0.008)	(0.057)	(0.003)	(0.023)	(0.000)	(0.849)
Highest parental educ.	10.922 ***	10.654 ***	24.278 ***	19.044 ***	14.569 ***	−1.818 ***	3.349 ***	3.287 ***	18.429 ***	−1.066 ***	3.848 ***
	(0.000)	(0.000)	(0.000)	(0.000)	(0.000)	(0.000)	(0.000)	(0.000)	(0.000)	(0.000)	(0.000)
# of children in hh	0.181	−0.896 ***	0.701 **	−3.641 ***	0.018 **	0.766 **	−1.116 ***	−0.500 ***	−0.112	0.129 ***	−1.340 ***
	(0.111)	(0.007)	(0.039)	(0.000)	(0.026)	(0.011)	(0.000)	(0.001)	(0.325)	(0.000)	(0.000)
# of adults in hh	0.132 *	−0.215 *	0.393 ***	−0.349 **	−0.076	0.006	−1.290 ***	0.101	0.399 **	0.084 *	−2.233 ***
	(0.056)	(0.057)	(0.000)	(0.014)	(0.189)	(0.261)	(0.000)	(0.581)	(0.022)	(0.080)	(0.000)
Financial situation	0.350 *	−2.117 *	0.001	−0.591	−0.009	0.041	−0.576 **	−1.172 ***	−0.200	0.138 **	−0.255 **
	(0.057)	(0.053)	(0.995)	(0.225)	(0.969)	(0.445)	(0.014)	(0.000)	(0.424)	(0.037)	(0.026)
Finance * highest educ.	0.377	0.833	−0.808	1.166	−0.394	0.005	1.069	1.453 ***	−2.266	−0.542 *	1.781 *
	(0.567)	(0.772)	(0.805)	(0.533)	(0.874)	(0.977)	(0.175)	(0.001)	(0.437)	(0.078)	(0.056)
*N*	3990	3051	3736	5518	5514	2617	6886	4243	2835	3468	3582

***, ** and * signify significance of the effects at the 1%, 5% and 10% levels, respectively.

**Table B4.** Detailed decomposition results for downward mobility.

	Austria	Switzerland	Germany	Estonia	France	Croatia	Luxembourg	Latvia
Probability of downward mobility								
Migrants	15.094 ***	6.309 ***	7.035 ***	20.728 ***	2.696 ***	6.383 ***	6.704 ***	14.800 ***
	(0.000)	(0.000)	(0.000)	(0.000)	(0.001)	(0.000)	(0.000)	(0.000)
Natives	11.425 ***	8.288 ***	14.519 ***	16.169 ***	4.589 ***	6.470 ***	13.147 ***	13.410 ***
	(0.000)	(0.000)	(0.000)	(0.000)	(0.000)	(0.000)	(0.000)	(0.000)
Mobility gap	3.669	−1.979	−7.484 ***	4.559 **	−1.893 *	−0.087	−6.443 ***	1.390
	(0.242)	(0.215)	(0.003)	(0.028)	(0.072)	(0.958)	(0.000)	(0.394)
Explained	0.280	−0.740 ***	−3.505 ***	0.611 *	−2.235 ***	−0.731 **	−5.458 ***	−0.322
	(0.370)	(0.001)	(0.000)	(0.081)	(0.000)	(0.011)	(0.000)	(0.155)
Unexplained	3.389	−1.239	−3.979 ***	3.948 **	0.342	0.644	−0.984	1.712
	(0.151)	(0.275)	(0.001)	(0.025)	(0.486)	(0.754)	(0.380)	(0.205)
Contribution of covariates to differences in characteristics (explained part)								
Birth year	0.165	−0.052	−0.497 ***	−0.814 ***	0.019	−0.211	−0.515	−0.356 **
	(0.297)	(0.811)	(0.003)	(0.000)	(0.309)	(0.190)	(0.550)	(0.042)
Cohort effects	0.011	−0.418	0.322 **	0.055	−0.066 **	0.121	−1.195	−0.155
	(0.948)	(0.101)	(0.026)	(0.719)	(0.043)	(0.283)	(0.155)	(0.393)
Gender	0.122 **	−0.087 ***	−0.040 ***	−0.198 ***	−0.038 ***	−0.012	−0.128 ***	0.499 ***
	(0.010)	(0.000)	(0.000)	(0.000)	(0.004)	(0.469)	(0.001)	(0.000)
Mother’s age at birth	0.331	0.264	−0.816	−0.253	−0.202	−1.461 **	0.154	−0.261
	(0.704)	(0.470)	(0.199)	(0.533)	(0.268)	(0.019)	(0.180)	(0.520)
Mother’s age at b., sq	−0.157	−0.291	1.091 *	0.277	0.173	1.299 **	0.102	0.249
	(0.852)	(0.404)	(0.089)	(0.432)	(0.362)	(0.014)	(0.526)	(0.515)
Father’s age at birth	−2.324 *	−0.101	−0.786	1.093 ***	−0.165	−0.106	−0.453 *	0.182
	(0.054)	(0.598)	(0.179)	(0.004)	(0.713)	(0.770)	(0.086)	(0.535)
Father’s age at b., sq	1.801	0.157	0.204	−1.249 ***	0.180	−0.012	0.402	−0.190
	(0.125)	(0.430)	(0.705)	(0.009)	(0.676)	(0.962)	(0.242)	(0.393)
Age difference parents	0.244 *	0.051 *	0.050 *	0.002	−0.042	0.042	0.166	−0.046
	(0.097)	(0.090)	(0.060)	(0.989)	(0.549)	(0.318)	(0.178)	(0.307)
Mother’s labour m. stat.	0.021	0.076	−0.003	−0.008	−0.027	0.049	−0.011	0.029
	(0.774)	(0.275)	(0.801)	(0.422)	(0.414)	(0.535)	(0.922)	(0.571)
Mother’s education	0.184 ***	0.116 *	1.120 ***	−0.996 ***	0.480 ***	0.587 ***	0.404 ***	0.783 ***
	(0.000)	(0.062)	(0.000)	(0.000)	(0.000)	(0.000)	(0.000)	(0.000)
Father’s education	0.136 *	0.024	0.978 ***	−1.707 ***	0.456 ***	0.342 ***	2.054 ***	−0.542 ***
	(0.080)	(0.303)	(0.000)	(0.000)	(0.000)	(0.000)	(0.000)	(0.000)
Highest parental educ.	0.019	−0.278 **	−4.151 ***	4.612 ***	−3.357 ***	−2.506 ***	−10.956 ***	−0.246 ***
	(0.917)	(0.047)	(0.000)	(0.000)	(0.000)	(0.000)	(0.000)	(0.000)
# of children in hh	−0.086	−0.202 *	−0.006	−0.242	−0.003	0.791 ***	0.063	−0.218 ***
	(0.157)	(0.078)	(0.886)	(0.241)	(0.955)	(0.000)	(0.314)	(0.000)
# of adults in hh	0.021	−0.059	0.012	0.046	0.025	0.053	−0.103	0.000
	(0.187)	(0.403)	(0.638)	(0.160)	(0.692)	(0.304)	(0.162)	(0.999)
Financial situation	−1.018 ***	0.140	1.262 ***	−0.014	−0.119	0.056	−0.672 ***	−0.075
	(0.007)	(0.394)	(0.001)	(0.549)	(0.488)	(0.817)	(0.000)	(0.231)
Finance * highest educ.	0.810 *	−0.078	−2.245 ***	0.000	0.454	0.234	5.230 ***	0.031
	(0.057)	(0.720)	(0.005)	(1.000)	(0.398)	(0.553)	(0.000)	(0.290)
*N*	4745	4395	8154	3505	8013	4609	3142	4251

***, ** and * signify significance of the effects at the 1%, 5% and 10% levels, respectively.

**Table B5.** Detailed decomposition results for upward mobility conditioned on parental education.

	Belgium	Switzerland	Czech Republic	Estonia	France	Croatia	Luxembourg	Latvia
Probability of upward mobility conditioned on parental education								
Migrants	75.248 ***	92.913 ***	76.543 ***	82.278 ***	82.647 ***	70.073 ***	71.250 ***	83.529 ***
	(0.000)	(0.000)	(0.000)	(0.000)	(0.000)	(0.000)	(0.000)	(0.000)
Natives	71.196 ***	83.810 ***	89.965 ***	83.612 ***	80.302 ***	68.255 ***	57.996 ***	79.787 ***
	(0.000)	(0.000)	(0.000)	(0.000)	(0.000)	(0.000)	(0.000)	(0.000)
Mobility gap	4.052	9.104 ***	−13.422 ***	−1.334	2.345	1.818	13.254 ***	3.742
	(0.381)	(0.008)	(0.000)	(0.760)	(0.290)	(0.657)	(0.000)	(0.249)
Explained	−0.662	4.919 ***	−6.129 ***	0.379	1.144 **	1.072	7.038 ***	1.650 ***
	(0.532)	(0.004)	(0.000)	(0.548)	(0.012)	(0.119)	(0.000)	(0.004)
Unexplained	4.714	4.185	−7.293 *	−1.713	1.200	0.746	6.216 *	2.093
	(0.200)	(0.121)	(0.057)	(0.580)	(0.551)	(0.833)	(0.073)	(0.499)
Contribution of covariates to differences in characteristics (explained part)								
Birth year	1.162	−0.620	−1.123	−0.016	3.306 ***	−0.167	7.367 *	0.983 **
	(0.474)	(0.873)	(0.123)	(0.873)	(0.000)	(0.896)	(0.096)	(0.011)
Cohort effects	2.094 *	2.885	−0.401	−0.031	−0.354	1.495	−1.079	1.176 **
	(0.099)	(0.445)	(0.650)	(0.927)	(0.533)	(0.216)	(0.790)	(0.014)
Gender	−0.024	0.434 ***	−0.349 ***	0.119 **	0.229 ***	−0.126 ***	0.885 ***	−0.568 ***
	(0.605)	(0.000)	(0.000)	(0.034)	(0.000)	(0.000)	(0.000)	(0.000)
Mother’s age at birth	−0.152	5.154	−0.340	0.009	1.134	−0.180	−1.916	2.441
	(0.816)	(0.215)	(0.823)	(0.966)	(0.106)	(0.914)	(0.294)	(0.137)
Mother’s age at b., sq	0.106	−5.284	−0.231	0.093	−1.393 **	−0.512	0.949	−1.737
	(0.786)	(0.188)	(0.882)	(0.844)	(0.047)	(0.750)	(0.520)	(0.258)
Father’s age at birth	4.791 *	−6.355	−0.160	0.319	0.577	2.271 **	−1.349	−0.049
	(0.059)	(0.152)	(0.712)	(0.871)	(0.753)	(0.011)	(0.496)	(0.730)
Father’s age at b., sq	−4.285 *	6.155	0.386	−1.153	0.399	−1.314 *	1.306	0.183
	(0.085)	(0.162)	(0.337)	(0.583)	(0.824)	(0.074)	(0.476)	(0.434)
Age difference parents	−1.178	0.061	0.004	0.665	−0.643 *	0.286 **	0.035	−0.290
	(0.168)	(0.856)	(0.876)	(0.105)	(0.057)	(0.019)	(0.807)	(0.478)
Mother’s labour m. stat.	−0.626 ***	0.187	−0.320 ***	−0.333	−0.121	0.563 **	−0.161	−0.093
	(0.004)	(0.638)	(0.002)	(0.134)	(0.291)	(0.025)	(0.659)	(0.614)
# of children in hh	−0.585 *	1.667 **	−3.356 ***	0.362	−0.793 ***	−0.501 **	0.010	−0.239 **
	(0.054)	(0.039)	(0.000)	(0.332)	(0.000)	(0.020)	(0.760)	(0.012)
# of adults in hh	−0.067	0.669 *	−0.133	−0.112 **	−1.011 ***	0.122	0.222	0.157
	(0.363)	(0.089)	(0.108)	(0.028)	(0.000)	(0.625)	(0.545)	(0.137)
Financial situation	−1.897 ***	−0.035	−0.106	0.459 *	−0.188 ***	−0.864 ***	0.766 ***	−0.325 *
	(0.000)	(0.918)	(0.215)	(0.063)	(0.000)	(0.000)	(0.002)	(0.086)

***, ** and * signify significance of the effects at the 1%, 5% and 10% levels, respectively.

**Table B6.** Detailed decomposition results for upward mobility of two education levels conditioned on parental education.

	Belgium	Switzerland	Germany	Estonia	France	Luxembourg	Latvia	UK
Probability of upward mobility								
Migrants	22.772 ***	17.323 ***	31.481 ***	20.253 ***	28.824 ***	15.417 ***	14.118 ***	46.512 ***
	(0.000)	(0.000)	(0.000)	(0.000)	(0.000)	(0.000)	(0.000)	(0.000)
Natives	25.660 ***	13.333 ***	26.972 ***	19.856 ***	25.196 ***	11.673 ***	15.805 ***	29.926 ***
	(0.000)	(0.000)	(0.000)	(0.000)	(0.000)	(0.000)	(0.000)	(0.000)
Mobility gap	−2.887	3.990	4.509	0.397	3.628	3.744	−1.688	16.586 ***
	(0.517)	(0.238)	(0.479)	(0.933)	(0.136)	(0.111)	(0.569)	(0.001)
Explained	−2.379 **	4.528 **	4.860 **	1.227 **	−1.225 ***	5.870 ***	−0.441	0.028
	(0.020)	(0.042)	(0.013)	(0.012)	(0.006)	(0.000)	(0.223)	(0.971)
Unexplained	−0.508	−0.539	−0.351	−0.830	4.853 **	−2.126	−1.246	16.557 ***
	(0.889)	(0.894)	(0.950)	(0.729)	(0.039)	(0.401)	(0.627)	(0.001)
Contribution of covariates to differences in characteristics (explained part)								
Birth year	−1.920	3.488	−2.306	−0.035	0.754	2.850	−0.028	0.593
	(0.154)	(0.431)	(0.552)	(0.612)	(0.170)	(0.337)	(0.867)	(0.375)
Cohort effects	1.933 *	−3.015	3.794	0.214	1.080 **	2.779	0.078	0.119
	(0.055)	(0.468)	(0.195)	(0.270)	(0.022)	(0.316)	(0.702)	(0.863)
Gender	−0.085	0.332 ***	0.622 ***	−0.017	−0.153 **	0.241	−0.429 ***	−0.112
	(0.106)	(0.004)	(0.000)	(0.682)	(0.023)	(0.105)	(0.000)	(0.124)
Mother’s age at birth	−0.358	8.673	3.880	0.028	1.683 **	−3.641 **	0.868	3.065 *
	(0.629)	(0.109)	(0.286)	(0.800)	(0.015)	(0.049)	(0.295)	(0.097)
Mother’s age at b., sq	0.120	−7.904	−3.778	−0.140	−1.458 **	3.067 *	−0.582	−2.820 *
	(0.793)	(0.105)	(0.320)	(0.598)	(0.035)	(0.057)	(0.389)	(0.096)
Father’s age at birth	2.536	−13.826 ***	3.313 **	−1.768	−1.208	−1.474	−1.473 **	1.522
	(0.291)	(0.002)	(0.018)	(0.304)	(0.523)	(0.434)	(0.040)	(0.449)
Father’s age at b., sq	−2.559	14.009 ***	−1.885 **	2.046	1.212	0.935	1.684 **	−0.354
	(0.276)	(0.001)	(0.048)	(0.257)	(0.510)	(0.601)	(0.047)	(0.835)
Age difference parents	−0.512	−0.276	1.175	0.057	−0.388	0.193	−0.178	−0.406
	(0.559)	(0.275)	(0.307)	(0.882)	(0.312)	(0.321)	(0.613)	(0.288)
Mother’s labour m. stat.	−0.163	−0.066	0.216	0.059	0.021	0.085	−0.068	0.080
	(0.513)	(0.913)	(0.637)	(0.734)	(0.838)	(0.810)	(0.522)	(0.711)
# of children in hh	−0.743 **	2.405 ***	0.158	0.792 ***	−1.334 ***	0.044	−0.172 ***	−0.672
	(0.029)	(0.007)	(0.352)	(0.000)	(0.000)	(0.184)	(0.003)	(0.134)
# of adults in hh	−0.273 ***	0.038	0.254	−0.206 **	−1.280 ***	0.543 **	0.058 **	−1.009 ***
	(0.006)	(0.916)	(0.390)	(0.036)	(0.000)	(0.026)	(0.017)	(0.000)
Financial situation	−0.354	0.671	−0.582	0.197	−0.158 ***	0.240	−0.202 *	0.023 *
	(0.424)	(0.102)	(0.110)	(0.237)	(0.000)	(0.170)	(0.082)	(0.084)

***, ** and * signify significance of the effects at the 1%, 5% and 10% levels, respectively.
