# Supplementary material for: Catching up? The educational mobility of migrants’ and natives’ children in Europe
Source: Appl Econ. 2016 Dec 30;49(37):3701–28. doi: 10.1080/00036846.2016.1267843 (PMC5448400; doi:10.1080/00036846.2016.1267843)
Supplement: intergenerational_persistence_migrants_appendix_tables.pdf [file raec_a_1267843_sm1840.pdf]

Online Appendix
Catching-up: The educational mobility of migrants' and natives' children in Europe*

Doris Oberdabernig†1 and Alyssa Schneebaum‡2

[1]World Trade Institute (WTI), University of Bern
[2]Department of Economics, Vienna University of Economics and Business

October 2016

Abstract

Migrants into European countries are often less educated than European natives. We analyze whether migrants' children are more or less likely than natives' children to achieve upward educational mobility across generations, and study differences in the factors which contribute to differences in mobility for the two groups. We find that migrants' descendants are more often upwardly mobile (and less often downwardly mobile) than their native peers in the majority of countries studied, and show that the main factor contributing to these patterns is the education level of parents. Although a lower parental education means that their children are less likely to access the same amount of human, social, and financial capital as children of more highly educated parents, migrants' descendants over the last two generations were able to make significant progress in reducing education gaps with natives.

JEL Classifications: J62, I21, J11

Key Words: Intergenerational Mobility; Immigrants; Educational Attainment; Europe

---

*The authors are grateful for helpful comments from Octavio Fernández-Amador and some people at some comferences. Sophie Augustin provided excellent research assistance.

†`doris.oberdabernig@wti.org`. Hallerstrasse 6, 3012 Bern, Switzerland. Tel.: +41 (0)31 631 3270

‡Corresponding Author. `alyssa.schneebaum@wu.ac.at`. Welthandelsplatz 1, 1020 Vienna, Austria. Tel.: +43 (0)1 31336 5936

Contents

A Upward mobility **3**
A.1 Descriptive statistics . . . 3
A.2 Logit coefficients . . . 3
A.3 Logit results - marginal effects . . . 6
A.4 Gelman statistics . . . 9

B Downward mobility **11**
B.1 Descriptive statistics . . . 11
B.2 Logit coefficients . . . 11
B.3 Logit results - marginal effects . . . 14
B.4 Gelman statistics . . . 17

C Upward mobility conditional on parental education **19**
C.1 Descriptive statistics . . . 19
C.2 Logit coefficients . . . 19
C.3 Logit results - marginal effects . . . 22
C.4 Gelman statistics . . . 25

D Two classes upward mobility **27**
D.1 Descriptive statistics . . . 27
D.2 Logit coefficients . . . 27
D.3 Logit results - marginal effects . . . 30
D.4 Gelman statistics . . . 33

E Selection **35**
E.1 Dropped observations . . . 35
E.2 Mobility patterns of dropped observations . . . 36

A Upward mobility

A.1 Descriptive statistics

Table A.1: Descriptive statistics of all variables for the sample used for upward mobility

	AT	BE	CH	CZ	DE	EE	FR	HR	LU	LV	UK
					Natives' children						
Birth year	1966	1966	1965	1966	1965	1966	1966	1965	1965	1967	1966
Cohort 50s (d)	0.27	0.31	0.32	0.32	0.33	0.29	0.31	0.35	0.35	0.28	0.30
Cohort 60s (d)	0.39	0.32	0.38	0.27	0.38	0.35	0.33	0.32	0.32	0.32	0.35
Male (d)	0.48	0.49	0.46	0.43	0.47	0.49	0.46	0.51	0.49	0.45	0.45
Fathers' age at birth	30.36	29.98	31.56	28.14	29.51	30.59	29.62	29.47	31.04	29.76	29.98
Mothers' age at birth	26.96	27.49	28.31	24.94	26.71	27.65	26.87	26.00	27.67	27.37	27.14
Age diff. of parents	4.26	3.23	4.11	3.63	3.58	4.34	3.59	4.19	4.17	3.91	3.62
Mother out of labor f. (d)	0.44	0.54	0.46	0.08	0.48	0.08	0.44	0.56	0.64	0.08	0.26
Mothers' education	1.36	1.24	1.50	1.24	1.65	1.52	1.07	1.19	1.29	1.48	1.09
Fathers' education	1.56	1.28	1.75	1.25	1.86	1.48	1.08	1.40	1.51	1.41	1.28
Highest parental educ.	1.61	1.38	1.82	1.37	1.90	1.65	1.14	1.42	1.56	1.56	1.33
# of adults in hh	2.76	2.38	2.54	2.17	2.30	2.29	2.44	2.63	2.61	2.17	2.30
# of children in hh	2.54	2.53	2.53	2.24	2.22	2.35	1.62	2.22	2.35	2.37	2.29
Financial situation	3.63	4.25	4.25	3.99	3.95	3.93	3.86	3.59	4.16	4.04	3.93
					Migrants' children						
Birth year	1971	1971	1969	1961	1965	1966	1970	1968	1972	1966	1968
Cohort 50s (d)	0.22	0.20	0.12	0.54	0.33	0.29	0.17	0.23	0.08	0.33	0.18
Cohort 60s (d)	0.16	0.22	0.38	0.30	0.40	0.36	0.32	0.34	0.28	0.32	0.41
Male (d)	0.44	0.51	0.46	0.39	0.46	0.52	0.51	0.49	0.52	0.52	0.40
Fathers' age at birth	30.87	31.94	31.13	28.71	31.35	29.05	32.48	30.55	30.72	29.81	31.77
Mothers' age at birth	27.74	27.51	27.63	26.26	28.03	27.59	27.54	27.40	27.36	28.00	28.43
Age diff of parents	4.15	5.21	4.37	3.65	4.10	3.32	5.37	4.00	4.32	3.22	4.53
Mother out of labor f. (d)	0.34	0.69	0.31	0.15	0.50	0.06	0.58	0.66	0.50	0.05	0.33
Mothers' education	1.16	0.91	1.09	1.07	1.39	1.56	0.84	1.12	1.12	1.46	0.99
Fathers' education	1.34	1.01	1.35	1.07	1.61	1.53	0.92	1.34	1.20	1.46	1.16
Highest parental educ.	1.46	1.08	1.42	1.14	1.65	1.67	0.99	1.37	1.27	1.58	1.19
# of adults in hh	2.66	2.51	2.40	2.32	2.38	2.28	2.95	2.89	2.42	2.15	2.72
# of children in hh	2.31	3.06	1.95	3.06	2.20	1.95	2.27	2.43	2.51	2.32	2.89
Financial situation	3.72	3.82	4.21	3.78	3.90	3.95	3.71	3.49	4.07	4.06	3.88

Age difference refers to the absolute age difference between the parents. Mother out of labor force is an indicator whether the respondents' mother was in or out of the labor force when the respondent was 14. # of persons in the household refers to the number of adults and children, respectively, in the household in which the respondents lived when they were 14 years old. Financial situation refers to the financial situation of the household in which the respondents lived when they were 14 years old. A detailed definition of variables can be found in the variable table A.3.

A.2 Logit coefficients

Table A.2: Logit result for natives' children

	Austria	Belgium	Switzerland	Czech Rep.	Germany	Estonia	France	Croatia	Luxemb.	Latvia	UK
Intercept	26.592	4.947	28.598	-59.731 **	37.069 **	46.024 *	-65.142 ***	-7.068	-28.190	-33.953	-41.350 *
	(0.229)	(0.832)	(0.245)	(0.012)	(0.033)	(0.097)	(0.000)	(0.732)	(0.279)	(0.146)	(0.084)
Birth year	-0.013	-0.003	-0.014	0.033 ***	-0.018 **	-0.022	0.033 ***	0.003	0.013	0.018	0.022 *
	(0.244)	(0.783)	(0.252)	(0.006)	(0.039)	(0.120)	(0.000)	(0.762)	(0.326)	(0.135)	(0.070)
Cohort 50s (d)	-0.523 **	-0.645 **	-0.516 *	0.171	-0.377 *	0.300	-0.246	-0.024	-0.421	1.168 ***	-0.229
	(0.043)	(0.017)	(0.063)	(0.545)	(0.059)	(0.356)	(0.235)	(0.925)	(0.179)	(0.000)	(0.417)
Cohort 60s (d)	-0.128	-0.070	-0.280	0.311 *	-0.196	0.377 *	0.010	0.211	-0.361 *	0.804 ***	-0.016
	(0.444)	(0.694)	(0.105)	(0.080)	(0.116)	(0.073)	(0.942)	(0.201)	(0.071)	(0.000)	(0.934)
Male (d)	0.516 ***	-0.215 ***	0.794 ***	0.284 ***	0.444 ***	-0.942 ***	0.133 **	0.326 ***	0.367 ***	-1.037 ***	0.010
	(0.000)	(0.007)	(0.000)	(0.000)	(0.000)	(0.000)	(0.024)	(0.000)	(0.000)	(0.000)	(0.913)
Mother's age at birth	0.126 **	0.107	0.163 **	0.044	0.104 **	0.026	0.108 *	-0.006	0.187 **	0.092	0.172 **
	(0.041)	(0.106)	(0.022)	(0.566)	(0.049)	(0.723)	(0.053)	(0.920)	(0.021)	(0.147)	(0.011)
Mother's age at b., sq (/100)	-0.236 **	-0.195 *	-0.235 **	-0.107	-0.205 **	-0.033	-0.208 **	-0.030	-0.297 **	-0.146	-0.258 **
	(0.023)	(0.062)	(0.040)	(0.432)	(0.019)	(0.774)	(0.026)	(0.764)	(0.029)	(0.158)	(0.018)
Father's age at birth	0.043	0.102 *	0.114 **	0.009	0.067	0.026	0.023	0.120 ***	0.085	0.021	-0.045
	(0.352)	(0.054)	(0.049)	(0.876)	(0.101)	(0.613)	(0.590)	(0.010)	(0.176)	(0.660)	(0.399)
Father's age at b., sq (/100)	-0.019	-0.103	-0.159 *	0.039	-0.022	-0.030	-0.001	-0.117 *	-0.085	-0.002	0.091
	(0.775)	(0.173)	(0.051)	(0.664)	(0.713)	(0.683)	(0.991)	(0.086)	(0.362)	(0.977)	(0.234)
Age difference parents	-0.020	-0.046 **	0.003	-0.013	-0.045 ***	-0.022	-0.023	-0.033 *	-0.017	-0.033 **	-0.029
	(0.228)	(0.016)	(0.888)	(0.574)	(0.007)	(0.235)	(0.104)	(0.053)	(0.462)	(0.023)	(0.149)
Mother out of labor f. (d)	0.014	-0.190 **	0.085	-0.529 ***	-0.021	0.093	-0.051	0.033	0.094	0.162	-0.076
	(0.866)	(0.033)	(0.320)	(0.000)	(0.754)	(0.658)	(0.457)	(0.693)	(0.325)	(0.333)	(0.427)
Mother's education	0.554 ***	0.376 **	0.377 ***	0.644 ***	0.317 ***	0.982 ***	0.420 ***	0.868 ***	0.611 ***	0.907 ***	0.111
	(0.000)	(0.010)	(0.000)	(0.000)	(0.000)	(0.000)	(0.004)	(0.000)	(0.000)	(0.000)	(0.436)
Father's education	0.335 *	0.362 **	0.210	0.624 ***	0.048	0.383 ***	0.290 *	0.795 ***	0.500 **	0.613 ***	-0.040
	(0.078)	(0.021)	(0.176)	(0.000)	(0.735)	(0.009)	(0.051)	(0.002)	(0.023)	(0.000)	(0.848)
Highest parental educ.	-3.369 ***	-1.669 ***	-3.516 ***	-4.425 ***	-2.776 ***	-3.650 ***	-1.596 ***	-2.668 ***	-2.810 ***	-2.777 ***	-1.636 ***
	(0.000)	(0.000)	(0.000)	(0.000)	(0.000)	(0.000)	(0.000)	(0.000)	(0.000)	(0.000)	(0.000)
# of children in hh	-0.046	-0.077 ***	-0.069 **	-0.267 ***	-0.051 *	-0.098 **	-0.120 ***	-0.108 ***	-0.035	-0.152 ***	-0.137 ***
	(0.111)	(0.006)	(0.041)	(0.000)	(0.053)	(0.012)	(0.000)	(0.001)	(0.324)	(0.000)	(0.000)
# of adults in hh	-0.067 *	-0.083 *	-0.166 ***	-0.169 ***	-0.043	-0.136 *	-0.155 ***	0.019	-0.096 **	-0.149 **	-0.313 ***
	(0.053)	(0.050)	(0.000)	(0.007)	(0.171)	(0.057)	(0.000)	(0.574)	(0.023)	(0.048)	(0.000)
Financial situation	0.209 *	0.226 **	-0.020	0.160	-0.010	0.168	0.238 ***	0.429 ***	0.109	0.339 **	0.301 **
	(0.063)	(0.050)	(0.915)	(0.219)	(0.963)	(0.470)	(0.008)	(0.000)	(0.423)	(0.028)	(0.019)
Finance * highest educ.	-0.039	-0.022	0.025	-0.054	0.018	0.009	-0.103	-0.222 ***	0.071	-0.161 *	-0.163 *
	(0.567)	(0.776)	(0.807)	(0.534)	(0.875)	(0.946)	(0.170)	(0.001)	(0.437)	(0.077)	(0.055)
N	3903	2911	3455	5415	5361	2378	6466	4024	2503	3059	3466

The table reports logit coefficients. p-value based on equation (3) in parenthesis. ***, (**), [*] signify significance of the effects at the 1%, (5%), [10%] level, respectively, based on equation (4). (d) indicates that the variable is a dummy variable. For dummy variables the reported effect refers to a discrete change of the variable from 0 to 1.

Table A.3: Logit result for migrants' children

	Austria	Belgium	Switzerland	Czech Rep.	Germany	Estonia	France	Croatia	Luxemb.	Latvia	UK
Intercept	360.785 *	-501.110 ***	70.506	256.015	-96.544	-279.350 **	32.063	77.771	-24.031	62.755	-245.567
	(0.058)	(0.003)	(0.503)	(0.200)	(0.542)	(0.018)	(0.685)	(0.413)	(0.744)	(0.446)	(0.492)
Birth year	-0.189 *	0.259 ***	-0.030	-0.129	0.051	0.136 **	-0.017	-0.042	0.015	-0.030	0.124
	(0.051)	(0.003)	(0.569)	(0.197)	(0.521)	(0.022)	(0.669)	(0.381)	(0.676)	(0.475)	(0.492)
Cohort 50s (d)	-3.417	4.378 **	-0.412	-2.480	0.878	2.945 **	-0.290	-0.874	-0.795	0.023	0.299
	(0.158)	(0.021)	(0.741)	(0.261)	(0.636)	(0.026)	(0.760)	(0.466)	(0.397)	(0.980)	(0.939)
Cohort 60s (d)	0.100	2.442 **	-0.280	0.242	-0.989	2.165 **	-0.193	0.327	0.059	0.210	-1.264
	(0.950)	(0.041)	(0.692)	(0.860)	(0.375)	(0.010)	(0.745)	(0.667)	(0.914)	(0.742)	(0.613)
Male (d)	1.103	-1.676 ***	1.267 ***	-0.257	0.091	-1.774 ***	0.330	0.184	0.593 **	-0.963 ***	-3.090 **
	(0.123)	(0.006)	(0.003)	(0.671)	(0.856)	(0.000)	(0.259)	(0.579)	(0.030)	(0.000)	(0.047)
Mother's age at birth	-0.589	0.588	-0.381	-0.143	0.882 *	0.318	0.059	-0.144	0.626 **	0.064	2.175 **
	(0.306)	(0.115)	(0.339)	(0.820)	(0.061)	(0.396)	(0.805)	(0.685)	(0.011)	(0.745)	(0.033)
Mother's age at b., sq (/100)	0.517	-0.777	0.703	0.445	-1.127	-0.716	-0.230	0.205	-1.327 ***	-0.059	-3.375 **
	(0.566)	(0.181)	(0.304)	(0.691)	(0.105)	(0.267)	(0.539)	(0.731)	(0.002)	(0.847)	(0.024)
Father's age at birth	1.504 ***	-0.139	0.313	0.405	0.100	0.446	0.236	0.509	-0.234	-0.250	-0.960
	(0.006)	(0.635)	(0.338)	(0.239)	(0.803)	(0.116)	(0.243)	(0.116)	(0.225)	(0.190)	(0.330)
Father's age at b., sq (/100)	-1.516 **	0.010	-0.386	-0.642	-0.453	-0.650	-0.133	-0.615	0.634 **	0.399	1.012
	(0.045)	(0.979)	(0.441)	(0.200)	(0.428)	(0.158)	(0.611)	(0.187)	(0.040)	(0.152)	(0.401)
Age difference parents	-0.489 **	0.048	-0.130	0.074	0.011	-0.024	-0.101	-0.055	-0.067	-0.034	0.640 *
	(0.042)	(0.744)	(0.144)	(0.592)	(0.936)	(0.740)	(0.262)	(0.565)	(0.337)	(0.522)	(0.077)
Mother out of labor f. (d)	-0.673	-0.366	-0.289	-0.284	-0.658	-0.175	-0.831 **	-0.290	0.170	0.250	-2.195
	(0.454)	(0.600)	(0.498)	(0.738)	(0.228)	(0.845)	(0.014)	(0.530)	(0.585)	(0.685)	(0.160)
Mother's education	-0.191	1.110	0.813 **	-1.669	0.004	-0.579	0.735 *	1.059 *	0.696	1.654 ***	-0.029
	(0.853)	(0.154)	(0.038)	(0.357)	(0.994)	(0.324)	(0.056)	(0.051)	(0.252)	(0.005)	(0.987)
Father's education	-1.326	2.123 **	-0.198	0.117	1.545	0.373	0.407	2.829 *	1.657 **	0.494	2.810
	(0.324)	(0.049)	(0.775)	(0.957)	(0.230)	(0.559)	(0.472)	(0.085)	(0.011)	(0.280)	(0.401)
Highest parental educ.	-2.374	-12.902 ***	-6.431 ***	-0.992	-9.404 ***	0.931	-2.598 *	-4.280 **	-12.017 ***	-2.729 *	-9.448
	(0.401)	(0.002)	(0.002)	(0.802)	(0.010)	(0.699)	(0.054)	(0.027)	(0.000)	(0.081)	(0.112)
# of children in hh	0.276	-0.562 ***	-0.008	-0.179	-0.121	-0.644 ***	0.094	-0.012	-0.246 **	-0.047	0.538
	(0.431)	(0.002)	(0.974)	(0.270)	(0.634)	(0.005)	(0.243)	(0.927)	(0.028)	(0.680)	(0.472)
# of adults in hh	-0.535	-0.496 **	-0.898 ***	-0.671 *	-0.029	-0.283	-0.129	-0.256 *	0.004	-0.052	0.192
	(0.109)	(0.049)	(0.003)	(0.059)	(0.935)	(0.395)	(0.121)	(0.056)	(0.980)	(0.827)	(0.718)
Financial situation	0.247	-1.375	-0.335	0.160	-2.107	2.691 **	0.086	0.495	-1.987 ***	0.808	0.305
	(0.771)	(0.115)	(0.647)	(0.854)	(0.138)	(0.019)	(0.802)	(0.121)	(0.000)	(0.128)	(0.863)
Finance * highest educ.	-0.042	1.386 *	0.296	-0.379	0.770	-1.280 **	-0.011	-0.346	1.791 ***	-0.536	0.092
	(0.944)	(0.072)	(0.482)	(0.629)	(0.295)	(0.037)	(0.970)	(0.148)	(0.000)	(0.115)	(0.933)
N	87	140	281	103	153	239	420	219	332	409	116

The table reports logit coefficients. p-value based on equation (3) in parenthesis. ***, (**), [*] signify significance of the effects at the 1%, (5%), [10%] level, respectively, based on equation (4). (d) indicates that the variable is a dummy variable. For dummy variables the reported effect refers to a discrete change of the variable from 0 to 1.

A.3 Logit results - marginal effects

Table A.4: Logit result for natives' children (marginal effects)

	Austria	Belgium	Switzerland	Czech Rep.	Germany	Estonia	France	Croatia	Luxemb.	Latvia	UK
Birth year	-0.002	-0.001	-0.002	0.004 ***	-0.004 **	-0.004	0.005 ***	0.001	0.003	0.003	0.003 *
	(0.243)	(0.782)	(0.252)	(0.006)	(0.039)	(0.120)	(0.000)	(0.762)	(0.326)	(0.135)	(0.069)
Cohort 50s (d)	-0.080 **	-0.137 **	-0.086 *	0.021	-0.076 *	0.053	-0.041	-0.004	-0.082	0.211 ***	-0.037
	(0.037)	(0.015)	(0.055)	(0.541)	(0.052)	(0.353)	(0.238)	(0.931)	(0.172)	(0.000)	(0.412)
Cohort 60s (d)	-0.020	-0.015	-0.048	0.036 *	-0.040	0.063 *	0.001	0.039	-0.071 *	0.140 ***	-0.003
	(0.445)	(0.691)	(0.102)	(0.078)	(0.113)	(0.073)	(0.949)	(0.201)	(0.067)	(0.000)	(0.926)
Male (d)	0.083 ***	-0.045 ***	0.141 ***	0.033 ***	0.093 ***	-0.159 ***	0.021 **	0.061 ***	0.074 ***	-0.182 ***	0.001
	(0.000)	(0.007)	(0.000)	(0.000)	(0.000)	(0.000)	(0.023)	(0.000)	(0.000)	(0.000)	(0.915)
Mother's age at birth	0.020 **	0.022	0.029 **	0.005	0.022 **	0.004	0.017 *	-0.001	0.038 **	0.016	0.027 **
	(0.041)	(0.106)	(0.022)	(0.566)	(0.049)	(0.723)	(0.053)	(0.920)	(0.020)	(0.147)	(0.011)
Mother's age at b., sq (/100)	-0.038 **	-0.041 *	-0.041 **	-0.013	-0.043 **	-0.005	-0.033 **	-0.006	-0.060 **	-0.025	-0.040 **
	(0.023)	(0.061)	(0.040)	(0.432)	(0.018)	(0.775)	(0.027)	(0.764)	(0.029)	(0.158)	(0.018)
Father's age at birth	0.007	0.021 *	0.020 **	0.001	0.014	0.004	0.004	0.022 ***	0.017	0.004	-0.007
	(0.352)	(0.054)	(0.049)	(0.876)	(0.101)	(0.612)	(0.590)	(0.010)	(0.176)	(0.660)	(0.399)
Father's age at b., sq (/100)	-0.003	-0.022	-0.028 *	0.005	-0.005	-0.005	-0.000	-0.022 *	-0.017	-0.000	0.014
	(0.775)	(0.172)	(0.051)	(0.665)	(0.712)	(0.684)	(0.991)	(0.086)	(0.362)	(0.978)	(0.234)
Age difference parents	-0.003	-0.010 **	0.000	-0.002	-0.009 ***	-0.004	-0.004	-0.006 *	-0.003	-0.006 **	-0.005
	(0.228)	(0.016)	(0.889)	(0.573)	(0.007)	(0.235)	(0.103)	(0.053)	(0.462)	(0.023)	(0.148)
Mother out of labor f. (d)	0.002	-0.040 **	0.015	-0.064 ***	-0.004	0.016	-0.008	0.006	0.019	0.028	-0.012
	(0.865)	(0.033)	(0.320)	(0.000)	(0.755)	(0.653)	(0.456)	(0.690)	(0.325)	(0.331)	(0.425)
Mother's education	0.089 ***	0.079 ***	0.066 ***	0.076 ***	0.066 ***	0.164 ***	0.067 ***	0.162 ***	0.123 ***	0.158 ***	0.017
	(0.000)	(0.010)	(0.000)	(0.000)	(0.000)	(0.000)	(0.004)	(0.000)	(0.000)	(0.000)	(0.438)
Father's education	0.054 *	0.076 **	0.037	0.074 ***	0.010	0.064 ***	0.046 *	0.148 ***	0.100 **	0.106 ***	-0.006
	(0.078)	(0.020)	(0.176)	(0.000)	(0.735)	(0.009)	(0.050)	(0.002)	(0.023)	(0.000)	(0.847)
Highest parental educ.	-0.541 ***	-0.349 ***	-0.617 ***	-0.523 ***	-0.579 ***	-0.609 ***	-0.255 ***	-0.497 ***	-0.564 ***	-0.482 ***	-0.254 ***
	(0.000)	(0.000)	(0.000)	(0.000)	(0.000)	(0.000)	(0.000)	(0.000)	(0.000)	(0.000)	(0.000)
# of children in hh	-0.007	-0.016 ***	-0.012 **	-0.032 ***	-0.011 *	-0.016 **	-0.019 ***	-0.020 ***	-0.007	-0.026 ***	-0.021 ***
	(0.111)	(0.005)	(0.042)	(0.000)	(0.053)	(0.012)	(0.000)	(0.001)	(0.324)	(0.000)	(0.000)
# of adults in hh	-0.011 *	-0.017 **	-0.029 ***	-0.020 ***	-0.009	-0.023 *	-0.025 ***	0.003	-0.019 **	-0.026 **	-0.049 ***
	(0.053)	(0.049)	(0.000)	(0.007)	(0.171)	(0.058)	(0.000)	(0.575)	(0.023)	(0.047)	(0.000)
Financial situation	0.034 *	0.047 **	-0.004	0.019	-0.002	0.028	0.038 ***	0.080 ***	0.022	0.059 **	0.047 **
	(0.063)	(0.050)	(0.915)	(0.219)	(0.963)	(0.469)	(0.008)	(0.000)	(0.423)	(0.028)	(0.019)
Finance * highest educ.	-0.006	-0.005	0.004	-0.006	0.004	0.001	-0.017	-0.041 ***	0.014	-0.028 *	-0.025 *
	(0.567)	(0.776)	(0.807)	(0.534)	(0.874)	(0.947)	(0.171)	(0.001)	(0.437)	(0.078)	(0.055)
N	3903	2911	3455	5415	5361	2378	6466	4024	2503	3059	3466

The table reports average marginal effects. The constant term is not shown. p-value based on equation (3) in parenthesis. ***, (**), [*] signify significance of the effects at the 1%, (5%), [10%] level, respectively, based on equation (4). (d) indicates that the variable is a dummy variable. For dummy variables the reported effect refers to a discrete change of the variable from 0 to 1.

Table A.5: Logit results for migrants' children (marginal effects)

	Austria	Belgium	Switzerland	Czech Rep.	Germany	Estonia	France	Croatia	Luxemb.	Latvia	UK
Birth year	-0.021 **	0.029 ***	-0.003	-0.016	0.006	0.017 **	-0.002	-0.007	0.002	-0.004	0.004
	(0.039)	(0.001)	(0.564)	(0.181)	(0.523)	(0.018)	(0.672)	(0.381)	(0.678)	(0.476)	(0.501)
Cohort 50s (d)	-0.275 *	0.300 ***	-0.039	-0.232	0.103	0.348 ***	-0.051	-0.136	-0.132	0.009	-0.013
	(0.089)	(0.000)	(0.739)	(0.220)	(0.600)	(0.006)	(0.684)	(0.449)	(0.369)	(0.941)	(0.924)
Cohort 60s (d)	0.015	0.214 **	-0.029	0.019	-0.123	0.269 ***	-0.027	0.055	0.007	0.032	-0.047
	(0.926)	(0.012)	(0.672)	(0.901)	(0.356)	(0.005)	(0.715)	(0.655)	(0.934)	(0.716)	(0.590)
Male (d)	0.130	-0.179 ***	0.124 ***	-0.033	0.011	-0.233 ***	0.040	0.031	0.092 **	-0.130 ***	-0.105 **
	(0.120)	(0.003)	(0.002)	(0.661)	(0.855)	(0.000)	(0.262)	(0.580)	(0.030)	(0.000)	(0.026)
Mother's age at birth	-0.067	0.066	-0.037	-0.018	0.105 *	0.040	0.007	-0.024	0.095 ***	0.009	0.076 **
	(0.307)	(0.111)	(0.337)	(0.815)	(0.057)	(0.401)	(0.809)	(0.683)	(0.008)	(0.745)	(0.027)
Mother's age at b., sq (/100)	0.060	-0.087	0.068	0.056	-0.135	-0.090	-0.027	0.035	-0.201 ***	-0.008	-0.118 **
	(0.562)	(0.178)	(0.300)	(0.688)	(0.101)	(0.269)	(0.542)	(0.729)	(0.001)	(0.845)	(0.017)
Father's age at birth	0.170 ***	-0.015	0.030	0.049	0.012	0.057	0.028	0.085	-0.035	-0.034	-0.034
	(0.003)	(0.636)	(0.338)	(0.237)	(0.810)	(0.112)	(0.243)	(0.111)	(0.223)	(0.193)	(0.335)
Father's age at b., sq (/100)	-0.171 **	0.001	-0.037	-0.078	-0.054	-0.082	-0.016	-0.102	0.096 **	0.054	0.035
	(0.038)	(0.980)	(0.442)	(0.197)	(0.432)	(0.154)	(0.611)	(0.184)	(0.037)	(0.155)	(0.406)
Age difference parents	-0.055 **	0.005	-0.013	0.009	0.001	-0.003	-0.012	-0.009	-0.010	-0.005	0.023 *
	(0.035)	(0.743)	(0.143)	(0.588)	(0.935)	(0.739)	(0.259)	(0.565)	(0.335)	(0.519)	(0.074)
Mother out of labor f. (d)	-0.070	-0.037	-0.028	-0.043	-0.079	-0.017	-0.096 ***	-0.045	0.025	0.035	-0.078
	(0.464)	(0.616)	(0.499)	(0.690)	(0.229)	(0.876)	(0.010)	(0.540)	(0.590)	(0.674)	(0.146)
Mother's education	-0.022	0.124	0.079 **	-0.205	0.000	-0.074	0.089 *	0.176 **	0.106	0.221 ***	-0.001
	(0.851)	(0.146)	(0.031)	(0.359)	(0.997)	(0.323)	(0.057)	(0.046)	(0.250)	(0.005)	(0.983)
Father's education	-0.150	0.236 **	-0.019	0.015	0.185	0.047	0.049	0.472 *	0.252 ***	0.066	0.100
	(0.319)	(0.042)	(0.773)	(0.955)	(0.226)	(0.563)	(0.472)	(0.082)	(0.010)	(0.280)	(0.403)
Highest parental educ.	-0.266	-1.436 ***	-0.624 ***	-0.120	-1.121 ***	0.118	-0.314 *	-0.713 **	-1.824 ***	-0.365 *	-0.331
	(0.406)	(0.001)	(0.001)	(0.802)	(0.007)	(0.700)	(0.053)	(0.023)	(0.000)	(0.080)	(0.103)
\# of children in hh	0.031	-0.062 ***	-0.001	-0.022	-0.014	-0.081 ***	0.011	-0.002	-0.037 **	-0.006	0.019
	(0.434)	(0.000)	(0.975)	(0.262)	(0.637)	(0.004)	(0.244)	(0.928)	(0.026)	(0.681)	(0.489)
\# of adults in hh	-0.060 *	-0.055 **	-0.087 ***	-0.082 **	-0.003	-0.035	-0.016	-0.043 **	0.001	-0.007	0.007
	(0.100)	(0.041)	(0.002)	(0.050)	(0.936)	(0.397)	(0.122)	(0.049)	(0.983)	(0.827)	(0.723)
Financial situation	0.027	-0.153	-0.033	0.020	-0.251	0.340 **	0.010	0.082	-0.302 ***	0.108	0.011
	(0.782)	(0.106)	(0.647)	(0.854)	(0.136)	(0.015)	(0.805)	(0.118)	(0.000)	(0.129)	(0.860)
Finance * highest educ.	-0.004	0.154 *	0.029	-0.046	0.092	-0.162 **	-0.001	-0.057	0.272 ***	-0.072	0.003
	(0.950)	(0.062)	(0.481)	(0.630)	(0.296)	(0.033)	(0.973)	(0.144)	(0.000)	(0.114)	(0.942)
N	87	140	281	103	153	239	420	219	332	409	116

The table reports average marginal effects. The constant term is not shown. p-value based on equation (3) in parenthesis. ***, (**), [*] signify significance of the effects at the 1%, (5%), [10%] level, respectively, based on equation (4). (d) indicates that the variable is a dummy variable. For dummy variables the reported effect refers to a discrete change of the variable from 0 to 1.

A.4 Gelman statistics

Table A.6: Gelman statistics

	Austria	Belgium	Switzerland	Czech Rep.	Germany	Estonia	France	Croatia	Luxemb.	Latvia	UK
	Natives' children										
Intercept	1.00	1.00	1.00	1.00	1.00	1.00	1.00	1.00	1.00	1.00	1.00
Birth year	1.00	1.00	1.00	1.00	1.00	1.00	1.00	1.00	1.00	1.00	1.00
Cohort 50s (d)	1.00	1.00	1.00	1.00	1.00	1.00	1.00	1.00	1.00	1.00	1.00
Cohort 60s (d)	1.00	1.00	1.00	1.00	1.00	1.00	1.00	1.00	1.00	1.00	1.00
Male (d)	1.00	1.00	1.00	1.00	1.00	1.00	1.00	1.00	1.00	1.00	1.00
Mother's age at birth	1.00	1.00	1.00	1.00	1.00	1.00	1.00	1.00	1.00	1.00	1.00
Mother's age at b., sq (/100)	1.00	1.00	1.00	1.00	1.00	1.00	1.00	1.00	1.00	1.00	1.00
Father's age at birth	1.00	1.00	1.00	1.00	1.00	1.00	1.00	1.00	1.00	1.00	1.00
Father's age at b., sq (/100)	1.00	1.00	1.00	1.00	1.00	1.00	1.00	1.00	1.00	1.00	1.00
Age difference parents	1.00	1.00	1.00	1.00	1.00	1.00	1.00	1.00	1.00	1.00	1.00
Mother out of labor f. (d)	1.00	1.00	1.00	1.00	1.00	1.00	1.00	1.00	1.00	1.00	1.00
Mother's education	1.00	1.00	1.00	1.00	1.00	1.00	1.00	1.00	1.00	1.00	1.00
Father's education	1.00	1.00	1.00	1.00	1.00	1.00	1.00	1.00	1.00	1.00	1.00
Highest parental educ.	1.00	1.00	1.00	1.00	1.00	1.00	1.00	1.00	1.00	1.00	1.00
# of children in hh	1.00	1.00	1.00	1.00	1.00	1.00	1.00	1.00	1.00	1.00	1.00
# of adults in hh	1.00	1.00	1.00	1.00	1.00	1.00	1.00	1.00	1.00	1.00	1.00
Financial situation	1.00	1.00	1.00	1.00	1.00	1.00	1.00	1.00	1.00	1.00	1.00
Finance * highest educ.	1.00	1.00	1.00	1.00	1.00	1.00	1.00	1.00	1.00	1.00	1.00
	Migrants' children										
Intercept	1.02	1.04	1.00	1.02	1.00	1.01	1.00	1.00	1.00	1.00	1.03
Birth year	1.02	1.04	1.00	1.02	1.00	1.01	1.00	1.00	1.00	1.00	1.03
Cohort 50s (d)	1.02	1.03	1.00	1.01	1.00	1.01	1.00	1.00	1.00	1.00	1.02
Cohort 60s (d)	1.01	1.02	1.00	1.01	1.00	1.01	1.00	1.00	1.00	1.00	1.01
Male (d)	1.00	1.01	1.00	1.00	1.00	1.00	1.00	1.00	1.00	1.00	1.01
Mother's age at birth	1.00	1.00	1.00	1.00	1.00	1.00	1.00	1.00	1.00	1.00	1.01
Mother's age at b., sq (/100)	1.00	1.00	1.00	1.00	1.00	1.00	1.00	1.00	1.00	1.00	1.01
Father's age at birth	1.00	1.00	1.00	1.00	1.00	1.00	1.00	1.00	1.00	1.00	1.00
Father's age at b., sq (/100)	1.00	1.00	1.00	1.00	1.00	1.00	1.00	1.00	1.00	1.00	1.00
Age difference parents	1.00	1.00	1.00	1.00	1.00	1.00	1.00	1.00	1.00	1.00	1.01
Mother out of labor f. (d)	1.00	1.00	1.00	1.00	1.00	1.00	1.00	1.00	1.00	1.00	1.00
Mother's education	1.00	1.00	1.00	1.00	1.00	1.00	1.00	1.00	1.00	1.00	1.00
Father's education	1.00	1.01	1.00	1.00	1.00	1.00	1.00	1.00	1.00	1.00	1.00
Highest parental educ.	1.00	1.01	1.00	1.00	1.00	1.00	1.00	1.00	1.00	1.00	1.00
# of children in hh	1.00	1.00	1.00	1.00	1.00	1.00	1.00	1.00	1.00	1.00	1.00
# of adults in hh	1.00	1.00	1.00	1.00	1.00	1.00	1.00	1.00	1.00	1.00	1.00
Financial situation	1.00	1.00	1.00	1.00	1.00	1.00	1.00	1.00	1.00	1.00	1.00
Finance * highest educ.	1.00	1.00	1.00	1.00	1.00	1.00	1.00	1.00	1.00	1.00	1.00

B Downward mobility

B.1 Descriptive statistics

Table B.1: Descriptive statistics of all variables for the sample used for downward mobility

	AT	CH	DE	EE	FR	HR	LU	LV
				Natives' children				
Birth year	1966	1966	1965	1968	1967	1966	1966	1968
Cohort 50s (d)	0.26	0.30	0.30	0.25	0.28	0.34	0.33	0.25
Cohort 60s (d)	0.39	0.37	0.38	0.33	0.31	0.31	0.32	0.31
Male (d)	0.48	0.46	0.47	0.49	0.47	0.50	0.49	0.45
Father's age at birth	30.32	31.58	29.63	30.12	29.60	29.51	31.10	29.67
Mother's age at birth	26.93	28.39	26.85	27.40	26.96	26.00	27.78	27.36
Age difference parents	4.22	4.05	3.56	4.09	3.50	4.22	4.12	3.82
Mother out of labor f. (d)	0.44	0.47	0.44	0.07	0.42	0.53	0.63	0.08
Mother's education	1.46	1.59	1.82	1.82	1.27	1.29	1.38	1.71
Father's education	1.77	1.93	2.20	1.73	1.32	1.51	1.65	1.62
Highest parental educ.	1.83	2.00	2.26	1.98	1.43	1.55	1.71	1.84
# of adults in hh	2.71	2.50	2.26	2.27	2.40	2.60	2.58	2.16
# of children in hh	2.51	2.51	2.19	2.28	1.57	2.19	2.34	2.30
Financial situation	3.72	4.36	4.13	4.01	3.97	3.68	4.24	4.11
				Migrants' children				
Birth year	1970	1969	1964	1967	1970	1968	1973	1966
Cohort 50s (d)	0.23	0.13	0.34	0.26	0.18	0.24	0.08	0.31
Cohort 60s (d)	0.18	0.38	0.38	0.35	0.31	0.32	0.27	0.31
Male (d)	0.43	0.47	0.47	0.49	0.50	0.49	0.53	0.52
Father's age at birth	31.75	31.09	31.28	29.08	32.20	30.44	30.91	29.88
Mother's age at birth	28.10	27.63	28.07	27.68	27.70	27.20	27.37	27.95
Age difference parents	4.48	4.32	3.96	3.18	4.99	4.06	4.44	3.28
Mother out of labor f. (d)	0.34	0.33	0.46	0.05	0.54	0.62	0.51	0.05
Mother's education	1.32	1.34	1.52	1.93	1.03	1.21	1.23	1.65
Father's education	1.63	1.69	1.91	1.90	1.14	1.46	1.33	1.69
Highest parental educ.	1.74	1.77	1.96	2.11	1.24	1.49	1.41	1.84
# of adults in hh	2.64	2.34	2.36	2.25	2.82	2.81	2.42	2.13
# of children in hh	2.28	1.97	2.11	1.89	2.05	2.45	2.47	2.22
Financial situation	3.91	4.29	3.95	4.04	3.80	3.55	4.15	4.12

Age difference refers to the absolute age difference between the parents. Mother out of labor force is an indicator whether the respondents' mother was in or out of the labor force when the respondent was 14. # of persons in the household refers to the number of adults and children, respectively, in the household in which the respondents lived when they were 14 years old. Financial situation refers to the financial situation of the household in which the respondents lived when they were 14 years old. A detailed definition of variables can be found in the variable table A.3.

B.2 Logit coefficients

Table B.2: Logit result for natives' children

	Austria	Switzerland	Germany	Estonia	France	Croatia	Luxemb.	Latvia
Intercept	-37.980	-0.145	-64.381 ***	-151.049 ***	-41.589	47.676	16.859	-60.535 **
	(0.224)	(0.997)	(0.002)	(0.000)	(0.206)	(0.235)	(0.640)	(0.026)
Birth year	0.016	-0.004	0.031 ***	0.075 ***	0.018	-0.027	-0.011	0.028 **
	(0.295)	(0.817)	(0.003)	(0.000)	(0.291)	(0.190)	(0.544)	(0.041)
Cohort 50s (d)	0.628 *	0.663	0.527 **	0.198	0.940 **	-0.535	0.638	-0.360
	(0.094)	(0.128)	(0.032)	(0.601)	(0.023)	(0.278)	(0.137)	(0.299)
Cohort 60s (d)	0.069	0.237	0.142	-0.029	0.371	-0.318	0.178	-0.558 **
	(0.769)	(0.390)	(0.336)	(0.893)	(0.153)	(0.306)	(0.514)	(0.012)
Male (d)	-0.293 **	-0.978 ***	-0.360 ***	0.823 ***	0.411 ***	0.111	-0.457 ***	1.170 ***
	(0.011)	(0.000)	(0.000)	(0.000)	(0.001)	(0.470)	(0.000)	(0.000)
Mother's age at birth	0.032	-0.077	-0.080	-0.052	-0.128	-0.326 **	-0.180	-0.049
	(0.696)	(0.480)	(0.192)	(0.523)	(0.267)	(0.015)	(0.111)	(0.533)
Mother's age at b., sq (/100)	-0.027	0.142	0.186 *	0.106	0.181	0.605 **	0.377 **	0.078
	(0.840)	(0.412)	(0.077)	(0.422)	(0.360)	(0.010)	(0.047)	(0.535)
Father's age at birth	-0.132 *	0.049	-0.064	-0.208 ***	-0.035	-0.032	-0.162 *	0.043
	(0.050)	(0.599)	(0.176)	(0.001)	(0.722)	(0.755)	(0.067)	(0.531)
Father's age at b., sq (/100)	0.157	-0.108	0.026	0.281 ***	0.060	0.001	0.157	-0.088
	(0.120)	(0.432)	(0.709)	(0.003)	(0.684)	(0.993)	(0.229)	(0.390)
Age difference parents	0.046 *	0.056 *	0.033 *	-0.000	-0.017	-0.041	0.043	0.022
	(0.086)	(0.080)	(0.064)	(0.988)	(0.541)	(0.301)	(0.164)	(0.292)
Mother out of labor f. (d)	-0.037	-0.154	-0.020	0.256	-0.126	0.123	0.014	-0.151
	(0.767)	(0.274)	(0.802)	(0.358)	(0.419)	(0.526)	(0.916)	(0.548)
Mother's education	-0.859 ***	-0.291 **	-0.561 ***	-1.120 ***	-0.658 ***	-0.891 ***	-0.660 ***	-0.926 ***
	(0.000)	(0.020)	(0.000)	(0.000)	(0.000)	(0.000)	(0.000)	(0.000)
Father's education	-0.989 ***	-0.387 *	-0.747 ***	-0.904 ***	-0.657 ***	-1.029 ***	-1.002 ***	-0.680 ***
	(0.000)	(0.066)	(0.000)	(0.000)	(0.000)	(0.000)	(0.000)	(0.000)
Highest parental educ.	3.890 ***	3.587 ***	2.886 ***	3.901 ***	3.840 ***	5.105 ***	5.818 ***	2.759 ***
	(0.000)	(0.000)	(0.000)	(0.000)	(0.000)	(0.000)	(0.000)	(0.000)
# of children in hh	0.063	0.097 *	0.004	0.066	-0.008	0.302 ***	0.053	0.220 ***
	(0.153)	(0.068)	(0.897)	(0.239)	(0.897)	(0.000)	(0.317)	(0.000)
# of adults in hh	0.186 ***	0.060	0.022	-0.120	0.026	0.109	0.103	0.014
	(0.000)	(0.413)	(0.612)	(0.164)	(0.746)	(0.202)	(0.147)	(0.903)
Financial situation	-0.598 ***	-0.283	-0.686 ***	-0.115	0.233	-0.025	0.945 ***	-0.296
	(0.008)	(0.390)	(0.001)	(0.742)	(0.473)	(0.923)	(0.001)	(0.219)
Finance * highest educ.	0.166 *	0.045	0.207 ***	-0.002	-0.096	-0.071	-0.558 ***	0.142
	(0.051)	(0.729)	(0.004)	(0.990)	(0.398)	(0.517)	(0.000)	(0.148)
N	4639	4078	7955	3148	7605	4374	2784	3751

The table reports logit coefficients. p-value based on equation (3) in parenthesis. ***, (**), [*] signify significance of the effects at the 1%, (5%), [10%] level, respectively, based on equation (4). (d) indicates that the variable is a dummy variable. For dummy variables the reported effect refers to a discrete change of the variable from 0 to 1.

Table B.3: Logit result for migrants' children

	Austria	Switzerland	Germany	Estonia	France	Croatia	Luxemb.	Latvia
Intercept	-186.105	-325.271 *	-193.005	5.058	157.463	685.791 **	145.636	-96.115
	(0.532)	(0.077)	(0.533)	(0.959)	(0.679)	(0.026)	(0.338)	(0.263)
Birth year	0.086	0.147	0.106	-0.007	-0.092	-0.368 **	-0.075	0.047
	(0.569)	(0.111)	(0.507)	(0.886)	(0.634)	(0.020)	(0.325)	(0.276)
Cohort 50s (d)	1.220	1.053	-0.045	-1.824	6.985	-9.428 **	-1.510	-0.331
	(0.735)	(0.629)	(0.988)	(0.148)	(0.149)	(0.012)	(0.538)	(0.749)
Cohort 60s (d)	2.560	0.136	-0.325	-1.228	1.866	-9.645 ***	-0.617	-0.619
	(0.297)	(0.913)	(0.865)	(0.103)	(0.453)	(0.002)	(0.615)	(0.377)
Male (d)	-0.513	-0.707	-0.709	1.510 ***	-0.528	1.175	-0.490	1.025 ***
	(0.631)	(0.351)	(0.527)	(0.000)	(0.757)	(0.254)	(0.443)	(0.002)
Mother's age at birth	-0.051	-1.171	-2.371	0.160	-1.622	-0.225	-2.494 ***	-0.138
	(0.958)	(0.183)	(0.228)	(0.647)	(0.225)	(0.881)	(0.001)	(0.593)
Mother's age at b., sq (/100)	0.043	1.560	2.517	-0.433	4.004	-0.824	3.508 ***	0.074
	(0.974)	(0.321)	(0.391)	(0.479)	(0.111)	(0.735)	(0.002)	(0.855)
Father's age at birth	-0.061	2.723 **	0.250	-0.311	0.839	0.757	2.058 ***	0.014
	(0.944)	(0.029)	(0.810)	(0.260)	(0.670)	(0.605)	(0.004)	(0.951)
Father's age at b., sq (/100)	0.372	-4.382 **	0.404	0.531	-2.602	-0.210	-2.711 ***	0.087
	(0.724)	(0.042)	(0.676)	(0.226)	(0.406)	(0.923)	(0.004)	(0.796)
Age difference parents	-0.259	-0.421 *	-0.426	0.030	0.230	-1.039	-0.303	-0.101
	(0.435)	(0.086)	(0.583)	(0.711)	(0.508)	(0.136)	(0.289)	(0.186)
Mother out of labor f. (d)	-1.863	2.765 ***	-2.396	-0.351	-3.922 *	1.699	0.490	0.390
	(0.201)	(0.000)	(0.100)	(0.704)	(0.074)	(0.165)	(0.490)	(0.632)
Mother's education	-2.227 *	-1.452 ***	-0.286	-0.676	-2.133 *	-3.326 ***	-1.214 *	-1.147 ***
	(0.063)	(0.004)	(0.725)	(0.103)	(0.093)	(0.003)	(0.063)	(0.002)
Father's education	-4.255 **	526.049	-0.016	0.455	-2.881 **	4.685	-2.367 ***	-1.029 **
	(0.012)	(0.402)	(0.994)	(0.268)	(0.029)	(0.256)	(0.008)	(0.012)
Highest parental educ.	11.962 ***	-520.290	8.007	4.214 *	13.802 **	11.953 **	8.238 ***	4.502 ***
	(0.004)	(0.407)	(0.121)	(0.060)	(0.015)	(0.011)	(0.002)	(0.001)
# of children in hh	-0.001	-0.252	0.149	0.518 **	1.044 **	0.375	-0.322	0.103
	(0.997)	(0.611)	(0.815)	(0.031)	(0.013)	(0.460)	(0.306)	(0.546)
# of adults in hh	-0.241	0.457	-0.371	-0.355	-0.251	0.199	0.794 *	-0.531
	(0.557)	(0.400)	(0.607)	(0.245)	(0.736)	(0.732)	(0.056)	(0.234)
Financial situation	1.451	0.897	-2.234	0.538	1.586	4.416 *	-1.529	-0.283
	(0.442)	(0.467)	(0.551)	(0.678)	(0.608)	(0.063)	(0.239)	(0.692)
Finance * highest educ.	-0.559	-0.374	0.428	-0.165	-0.254	-2.023 *	-0.103	-0.053
	(0.469)	(0.433)	(0.744)	(0.744)	(0.832)	(0.065)	(0.853)	(0.854)
N	106	317	199	357	408	235	358	500

The table reports logit coefficients. p-value based on equation (3) in parenthesis. ***, (**), [*] signify significance of the effects at the 1%, (5%), [10%] level, respectively, based on equation (4). (d) indicates that the variable is a dummy variable. For dummy variables the reported effect refers to a discrete change of the variable from 0 to 1.

B.3 Logit results - marginal effects

Table B.4: Logit results for natives' children (marginal effects)

	Austria	Switzerland	Germany	Estonia	France	Croatia	Luxemb.	Latvia
Birth year	0.001	-0.000	0.003 ***	0.007 ***	0.001	-0.001	-0.001	0.002 **
	(0.294)	(0.817)	(0.003)	(0.000)	(0.291)	(0.190)	(0.548)	(0.040)
Cohort 50s (d)	0.044	0.041	0.053 **	0.020	0.037 **	-0.020	0.064	-0.027
	(0.107)	(0.147)	(0.037)	(0.579)	(0.040)	(0.284)	(0.155)	(0.301)
Cohort 60s (d)	0.005	0.014	0.014	-0.003	0.013	-0.012	0.017	-0.044 ***
	(0.760)	(0.390)	(0.335)	(0.898)	(0.166)	(0.311)	(0.506)	(0.009)
Male (d)	-0.019 **	-0.053 ***	-0.034 ***	0.075 ***	0.014 ***	0.004	-0.042 ***	0.100 ***
	(0.010)	(0.000)	(0.000)	(0.000)	(0.001)	(0.471)	(0.000)	(0.000)
Mother's age at birth	0.002	-0.004	-0.008	-0.005	-0.004	-0.013 **	-0.016	-0.004
	(0.695)	(0.480)	(0.191)	(0.523)	(0.267)	(0.015)	(0.111)	(0.532)
Mother's age at b., sq (/100)	-0.002	0.008	0.018 *	0.010	0.006	0.025 **	0.035 **	0.007
	(0.838)	(0.412)	(0.076)	(0.423)	(0.359)	(0.010)	(0.047)	(0.534)
Father's age at birth	-0.008 *	0.003	-0.006	-0.019 ***	-0.001	-0.001	-0.015 *	0.004
	(0.051)	(0.600)	(0.176)	(0.001)	(0.723)	(0.756)	(0.067)	(0.532)
Father's age at b., sq (/100)	0.010	-0.006	0.003	0.026 ***	0.002	0.000	0.014	-0.007
	(0.121)	(0.431)	(0.709)	(0.003)	(0.685)	(0.993)	(0.229)	(0.391)
Age difference parents	0.003 *	0.003 *	0.003 *	-0.000	-0.001	-0.002	0.004	0.002
	(0.087)	(0.080)	(0.063)	(0.989)	(0.542)	(0.301)	(0.162)	(0.293)
Mother out of labor f. (d)	-0.002	-0.009	-0.002	0.025	-0.004	0.005	0.001	-0.011
	(0.770)	(0.274)	(0.804)	(0.351)	(0.421)	(0.525)	(0.927)	(0.569)
Mother's education	-0.055 ***	-0.016 **	-0.054 ***	-0.102 ***	-0.022 ***	-0.036 ***	-0.061 ***	-0.077 ***
	(0.000)	(0.021)	(0.000)	(0.000)	(0.000)	(0.000)	(0.000)	(0.000)
Father's education	-0.063 ***	-0.022 *	-0.071 ***	-0.082 ***	-0.022 ***	-0.042 ***	-0.092 ***	-0.057 ***
	(0.000)	(0.066)	(0.000)	(0.000)	(0.000)	(0.000)	(0.000)	(0.000)
Highest parental educ.	0.249 ***	0.199 ***	0.276 ***	0.356 ***	0.126 ***	0.208 ***	0.534 ***	0.230 ***
	(0.000)	(0.000)	(0.000)	(0.000)	(0.000)	(0.000)	(0.000)	(0.000)
# of children in hh	0.004	0.005 *	0.000	0.006	-0.000	0.012 ***	0.005	0.018 ***
	(0.152)	(0.067)	(0.897)	(0.238)	(0.898)	(0.000)	(0.316)	(0.000)
# of adults in hh	0.012 ***	0.003	0.002	-0.011	0.001	0.004	0.009	0.001
	(0.001)	(0.414)	(0.613)	(0.164)	(0.746)	(0.199)	(0.146)	(0.903)
Financial situation	-0.038 ***	-0.016	-0.066 ***	-0.011	0.008	-0.001	0.087 ***	-0.025
	(0.007)	(0.391)	(0.001)	(0.741)	(0.473)	(0.924)	(0.001)	(0.218)
Finance * highest educ.	0.011 *	0.003	0.020 ***	-0.000	-0.003	-0.003	-0.051 ***	0.012
	(0.050)	(0.728)	(0.004)	(0.991)	(0.398)	(0.514)	(0.000)	(0.147)
N	4639	4078	7955	3148	7605	4374	2784	3751

The table reports average marginal effects. The constant term is not shown. p-value based on equation (3) in parenthesis. ***, (**), [*] signify significance of the effects at the 1%, (5%), [10%] level, respectively, based on equation (4). (d) indicates that the variable is a dummy variable. For dummy variables the reported effect refers to a discrete change of the variable from 0 to 1.

Table B.5: Logit results for migrants' children (marginal effects)

	Austria	Switzerland	Germany	Estonia	France	Croatia	Luxemb.	Latvia
Intercept	-10.085	-10.748 *	-5.108	0.412	1.409	16.828 **	4.703	-7.747
	(0.539)	(0.079)	(0.535)	(0.962)	(0.677)	(0.019)	(0.339)	(0.262)
Birth year	0.005	0.005	0.003	-0.001	-0.001	-0.009 **	-0.002	0.004
	(0.577)	(0.114)	(0.509)	(0.890)	(0.631)	(0.013)	(0.327)	(0.275)
Cohort 50s (d)	0.088	0.064	-0.000	-0.129	0.067	-0.140 ***	-0.021	-0.020
	(0.638)	(0.513)	(0.999)	(0.122)	(0.232)	(0.002)	(0.712)	(0.805)
Cohort 60s (d)	0.152	0.006	-0.004	-0.104 *	0.017	-0.155 ***	-0.017	-0.044
	(0.283)	(0.878)	(0.939)	(0.093)	(0.433)	(0.000)	(0.652)	(0.388)
Male (d)	-0.026	-0.022	-0.018	0.128 ***	-0.004	0.027	-0.016	0.080 ***
	(0.640)	(0.345)	(0.536)	(0.000)	(0.751)	(0.235)	(0.441)	(0.001)
Mother's age at birth	-0.003	-0.038	-0.062	0.014	-0.014	-0.006	-0.081 ***	-0.011
	(0.958)	(0.188)	(0.217)	(0.646)	(0.215)	(0.875)	(0.000)	(0.591)
Mother's age at b., sq (/100)	0.002	0.051	0.065	-0.037	0.035	-0.020	0.113 ***	0.006
	(0.977)	(0.328)	(0.387)	(0.477)	(0.101)	(0.742)	(0.001)	(0.853)
Father's age at birth	-0.003	0.090 **	0.007	-0.027	0.008	0.019	0.067 ***	0.001
	(0.950)	(0.030)	(0.806)	(0.258)	(0.661)	(0.609)	(0.002)	(0.947)
Father's age at b., sq (/100)	0.020	-0.144 **	0.011	0.046	-0.023	-0.006	-0.088 ***	0.007
	(0.730)	(0.043)	(0.683)	(0.224)	(0.404)	(0.918)	(0.002)	(0.802)
Age difference parents	-0.014	-0.014 *	-0.011	0.003	0.002	-0.026	-0.010	-0.008
	(0.425)	(0.089)	(0.582)	(0.712)	(0.511)	(0.161)	(0.291)	(0.182)
Mother out of labor f. (d)	-0.088	0.107 ***	-0.052 *	-0.022	-0.027 **	0.042	0.016	0.042
	(0.165)	(0.000)	(0.065)	(0.764)	(0.029)	(0.162)	(0.492)	(0.554)
Mother's education	-0.121 **	-0.048 ***	-0.007	-0.059	-0.018 *	-0.082 ***	-0.039 *	-0.093 ***
	(0.048)	(0.003)	(0.732)	(0.101)	(0.069)	(0.002)	(0.059)	(0.001)
Father's education	-0.232 ***	17.657	-0.000	0.039	-0.025 **	0.114	-0.076 ***	-0.083 **
	(0.004)	(0.424)	(0.998)	(0.269)	(0.017)	(0.251)	(0.004)	(0.010)
Highest parental educ.	0.653 ***	-17.467	0.209	0.365 *	0.120 **	0.295 ***	0.266 ***	0.363 ***
	(0.001)	(0.428)	(0.112)	(0.057)	(0.011)	(0.008)	(0.001)	(0.000)
# of children in hh	-0.000	-0.008	0.004	0.045 **	0.009 ***	0.009	-0.010	0.008
	(0.991)	(0.616)	(0.807)	(0.028)	(0.004)	(0.459)	(0.307)	(0.546)
# of adults in hh	-0.013	0.015	-0.010	-0.031	-0.002	0.005	0.026 *	-0.043
	(0.556)	(0.407)	(0.610)	(0.242)	(0.746)	(0.742)	(0.054)	(0.231)
Financial situation	0.080	0.030	-0.059	0.046	0.014	0.108 *	-0.049	-0.023
	(0.444)	(0.465)	(0.557)	(0.681)	(0.609)	(0.055)	(0.236)	(0.691)
Finance * highest educ.	-0.031	-0.012	0.012	-0.014	-0.002	-0.050 *	-0.003	-0.004
	(0.472)	(0.430)	(0.745)	(0.745)	(0.823)	(0.058)	(0.851)	(0.858)
N	106	317	199	357	408	235	358	500

The table reports average marginal effects. The constant term is not shown. p-value based on equation (3) in parenthesis. ***, (**), [*] signify significance of the effects at the 1%, (5%), [10%] level, respectively, based on equation (4). (d) indicates that the variable is a dummy variable. For dummy variables the reported effect refers to a discrete change of the variable from 0 to 1.

B.4 Gelman statistics

Table B.6: Gelman statistics

	Austria	Switzerland	Germany	Estonia	France	Croatia	Luxemb.	Latvia
	Natives' children							
Intercept	1.00	1.00	1.00	1.00	1.00	1.00	1.00	1.00
Birth year	1.00	1.00	1.00	1.00	1.00	1.00	1.00	1.00
Cohort 50s (d)	1.00	1.00	1.00	1.00	1.00	1.00	1.00	1.00
Cohort 60s (d)	1.00	1.00	1.00	1.00	1.00	1.00	1.00	1.00
Male (d)	1.00	1.00	1.00	1.00	1.00	1.00	1.00	1.00
Mother's age at birth	1.00	1.00	1.00	1.00	1.00	1.00	1.00	1.00
Mother's age at b., sq (/100)	1.00	1.00	1.00	1.00	1.00	1.00	1.00	1.00
Father's age at birth	1.00	1.00	1.00	1.00	1.00	1.00	1.00	1.00
Father's age at b., sq (/100)	1.00	1.00	1.00	1.00	1.00	1.00	1.00	1.00
Age difference parents	1.00	1.00	1.00	1.00	1.00	1.00	1.00	1.00
Mother out of labor f. (d)	1.00	1.00	1.00	1.00	1.00	1.00	1.00	1.00
Mother's education	1.00	1.00	1.00	1.00	1.00	1.00	1.00	1.00
Father's education	1.00	1.00	1.00	1.00	1.00	1.00	1.00	1.00
Highest parental educ.	1.00	1.00	1.00	1.00	1.00	1.00	1.00	1.00
# of children in hh	1.00	1.00	1.00	1.00	1.00	1.00	1.00	1.00
# of adults in hh	1.00	1.00	1.00	1.00	1.00	1.00	1.00	1.00
Financial situation	1.00	1.00	1.00	1.00	1.00	1.00	1.00	1.00
Finance * highest educ.	1.00	1.00	1.00	1.00	1.00	1.00	1.00	1.00
	Migrants' children							
Intercept	1.01	1.01	1.02	1.00	1.01	1.06	1.00	1.00
Birth year	1.01	1.01	1.02	1.00	1.01	1.06	1.00	1.00
Cohort 50s (d)	1.00	1.00	1.02	1.00	1.02	1.05	1.00	1.00
Cohort 60s (d)	1.00	1.00	1.01	1.00	1.01	1.05	1.00	1.00
Male (d)	1.00	1.00	1.00	1.00	1.01	1.00	1.00	1.00
Mother's age at birth	1.00	1.00	1.01	1.00	1.01	1.01	1.00	1.00
Mother's age at b., sq (/100)	1.00	1.00	1.01	1.00	1.02	1.01	1.00	1.00
Father's age at birth	1.00	1.01	1.00	1.00	1.03	1.00	1.00	1.00
Father's age at b., sq (/100)	1.00	1.01	1.00	1.00	1.03	1.00	1.00	1.00
Age difference parents	1.00	1.00	1.01	1.00	1.01	1.00	1.00	1.00
Mother out of labor f. (d)	1.00	1.00	1.01	1.00	1.01	1.00	1.00	1.00
Mother's education	1.00	1.00	1.00	1.00	1.00	1.01	1.00	1.00
Father's education	1.00	2.78	1.00	1.00	1.01	1.00	1.00	1.00
Highest parental educ.	1.00	2.78	1.01	1.00	1.07	1.02	1.00	1.00
# of children in hh	1.00	1.00	1.00	1.00	1.02	1.00	1.00	1.00
# of adults in hh	1.00	1.00	1.00	1.00	1.01	1.00	1.00	1.00
Financial situation	1.00	1.00	1.00	1.00	1.03	1.02	1.01	1.00
Finance * highest educ.	1.00	1.00	1.00	1.00	1.02	1.02	1.00	1.00

C Upward mobility conditional on parental education

C.1 Descriptive statistics

Table C.1: Descriptive statistics of all variables for the sample used for upward mobility

	BE	CH	CZ	EE	FR	HR	LU	LV
	Natives' children							
Birth year	1964	1961	1965	1961	1965	1963	1963	1962
Cohort 50s (d)	0.38	0.48	0.38	0.50	0.33	0.44	0.43	0.45
Cohort 60s (d)	0.35	0.36	0.27	0.36	0.34	0.36	0.35	0.35
Male (d)	0.49	0.43	0.43	0.49	0.45	0.49	0.48	0.46
Father's age at birth	30.76	34.14	28.31	32.86	29.81	30.27	31.63	31.58
Mother's age at birth	28.16	29.77	25.08	29.35	26.97	26.86	27.94	28.94
Age difference parents	3.32	5.07	3.69	5.20	3.67	4.24	4.50	4.33
Mother out of labor f. (d)	0.60	0.51	0.10	0.15	0.46	0.69	0.62	0.11
# of adults in hh	2.49	2.94	2.21	2.37	2.48	2.75	2.73	2.23
# of children in hh	2.67	2.91	2.33	2.50	1.67	2.36	2.57	2.61
Financial situation	4.16	3.81	3.87	3.78	3.80	3.25	3.91	3.87
	Migrants' children							
Birth year	1969	1971	1961	1961	1969	1967	1972	1961
Cohort 50s (d)	0.28	0.13	0.57	0.46	0.20	0.25	0.08	0.45
Cohort 60s (d)	0.24	0.27	0.31	0.38	0.30	0.37	0.28	0.42
Male (d)	0.51	0.46	0.36	0.49	0.52	0.48	0.55	0.51
Father's age at birth	32.45	31.41	28.90	31.11	32.49	31.15	30.49	31.24
Mother's age at birth	27.65	27.60	26.27	29.32	27.73	28.20	27.17	29.87
Age difference parents	5.31	4.61	3.81	3.95	5.18	3.95	4.31	3.19
Mother out of labor f. (d)	0.72	0.32	0.15	0.08	0.57	0.82	0.49	0.06
# of adults in hh	2.58	2.54	2.31	2.43	2.92	3.10	2.43	2.19
# of children in hh	3.19	1.93	3.14	2.05	2.11	2.63	2.53	2.71
Financial situation	3.69	4.15	3.72	3.96	3.72	3.07	4.08	3.73

Age difference refers to the absolute age difference between the parents. Mother out of labor force is an indicator whether the respondents' mother was in or out of the labor force when the respondent was 14. # of persons in the household refers to the number of adults and children, respectively, in the household in which the respondents lived when they were 14 years old. Financial situation refers to the financial situation of the household in which the respondents lived when they were 14 years old. A detailed definition of variables can be found in the variable table A.3.

C.2 Logit coefficients

Table C.2: Logit result for natives' children

	Belgium	Switzerland	Czech Rep.	Estonia	France	Croatia	Luxemb.	Latvia
Intercept	-27.328	13.563	-56.200	31.165	-112.603 ***	1.308	-69.261 *	113.857 **
	(0.444)	(0.859)	(0.154)	(0.610)	(0.000)	(0.964)	(0.084)	(0.016)
Birth year	0.013	-0.006	0.031	-0.015	0.057 ***	-0.002	0.033 *	-0.059 **
	(0.469)	(0.885)	(0.125)	(0.633)	(0.000)	(0.901)	(0.099)	(0.014)
Cohort 50s (d)	-0.816 **	-0.711	-0.240	0.650	0.140	-0.383	0.149	0.263
	(0.039)	(0.408)	(0.595)	(0.349)	(0.585)	(0.251)	(0.743)	(0.608)
Cohort 60s (d)	-0.241	-0.148	0.231	1.429 ***	0.219	0.063	-0.056	1.016 ***
	(0.346)	(0.804)	(0.432)	(0.002)	(0.189)	(0.770)	(0.847)	(0.002)
Gender (d)	-0.056	1.031 ***	0.831 ***	-0.782 ***	0.288 ***	0.777 ***	0.594 ***	-0.846 ***
	(0.608)	(0.000)	(0.000)	(0.000)	(0.000)	(0.000)	(0.000)	(0.000)
Mother's age at birth	0.016	-0.235	-0.025	-0.013	0.092	-0.008	0.110	0.149
	(0.855)	(0.220)	(0.813)	(0.925)	(0.109)	(0.911)	(0.294)	(0.137)
Mother's age at b., sq (/100)	-0.027	0.385	-0.021	-0.034	-0.192 **	-0.037	-0.113	-0.177
	(0.838)	(0.196)	(0.911)	(0.880)	(0.049)	(0.754)	(0.519)	(0.264)
Father's age at birth	0.145 *	0.202	-0.040	-0.020	0.014	0.140 **	0.053	0.040
	(0.054)	(0.140)	(0.646)	(0.842)	(0.761)	(0.012)	(0.500)	(0.597)
Father's age at b., sq (/100)	-0.193 *	-0.268	0.138	0.083	0.016	-0.143 *	-0.081	-0.100
	(0.079)	(0.150)	(0.312)	(0.558)	(0.816)	(0.075)	(0.479)	(0.350)
Age difference parents	-0.032	-0.009	-0.031	-0.053 *	-0.029 *	-0.051 **	-0.010	0.019
	(0.167)	(0.867)	(0.309)	(0.092)	(0.054)	(0.017)	(0.767)	(0.476)
Mother out of labor f. (d)	-0.345 ***	-0.106	-0.582 ***	0.456	-0.076	0.222 **	0.059	0.130
	(0.005)	(0.637)	(0.000)	(0.156)	(0.291)	(0.024)	(0.663)	(0.596)
# of children in hh	-0.063 **	-0.151 **	-0.316 ***	-0.060	-0.127 ***	-0.087 **	-0.010	-0.152 ***
	(0.045)	(0.028)	(0.000)	(0.335)	(0.000)	(0.016)	(0.801)	(0.002)
# of adults in hh	-0.044	-0.131 *	-0.148 *	-0.368 ***	-0.146 ***	0.020	-0.032	-0.161
	(0.363)	(0.068)	(0.074)	(0.001)	(0.000)	(0.608)	(0.549)	(0.125)
Financial situation	0.215 ***	-0.013	0.073	0.210 *	0.140 ***	0.219 ***	0.191 ***	0.135 *
	(0.000)	(0.894)	(0.190)	(0.050)	(0.000)	(0.000)	(0.002)	(0.078)
N	1781	630	3428	836	5493	2309	1088	1316

The table reports logit coefficients. p-value based on equation (3) in parenthesis. ***, (**), [*] signify significance of the effects at the 1%, (5%), [10%] level, respectively, based on equation (4). (d) indicates that the variable is a dummy variable. For dummy variables the reported effect refers to a discrete change of the variable from 0 to 1.

Table C.3: Logit result for migrants' children

	Belgium	Switzerland	Czech Rep.	Estonia	France	Croatia	Luxemb.	Latvia
Intercept	-286.379	-364.882	516.266 **	-66.702	48.013	-45.892	14.502	365.495 **
	(0.130)	(0.182)	(0.029)	(0.876)	(0.574)	(0.711)	(0.867)	(0.031)
Birth year	0.148	0.195	-0.263 **	0.031	-0.025	0.020	-0.007	-0.183 **
	(0.122)	(0.160)	(0.027)	(0.888)	(0.565)	(0.750)	(0.866)	(0.033)
Cohort 50s (d)	2.370	2.938	-5.116 *	-0.546	-0.251	0.396	-1.144	-2.652
	(0.280)	(0.342)	(0.057)	(0.911)	(0.806)	(0.790)	(0.294)	(0.178)
Cohort 60s (d)	1.444	2.180	-1.820	-1.062	-0.411	0.757	-0.049	-0.770
	(0.308)	(0.255)	(0.318)	(0.745)	(0.505)	(0.417)	(0.938)	(0.564)
Gender (d)	-1.188 *	0.948	-0.398	-4.808 ***	0.411	0.522	0.662 **	-0.746
	(0.056)	(0.348)	(0.588)	(0.005)	(0.178)	(0.279)	(0.038)	(0.136)
Mother's age at birth	0.731	-2.178	0.294	0.475	-0.047	-0.215	0.460 *	0.652
	(0.162)	(0.205)	(0.668)	(0.667)	(0.851)	(0.640)	(0.092)	(0.215)
Mother's age at b., sq (/100)	-0.601	5.117	-0.106	-0.343	-0.175	0.233	-1.016 **	-1.089
	(0.369)	(0.136)	(0.927)	(0.851)	(0.643)	(0.758)	(0.029)	(0.210)
Father's age at birth	-0.708	0.477	0.202	0.421	0.190	0.546	-0.286	-1.056 *
	(0.170)	(0.597)	(0.632)	(0.551)	(0.421)	(0.273)	(0.189)	(0.099)
Father's age at b., sq (/100)	0.428	-1.158	-0.607	-1.082	0.009	-0.528	0.728 **	1.840 *
	(0.413)	(0.465)	(0.284)	(0.325)	(0.976)	(0.467)	(0.043)	(0.075)
Age difference parents	0.306	0.220	0.286	0.465	-0.178 *	0.034	-0.092	0.240 *
	(0.335)	(0.527)	(0.223)	(0.105)	(0.090)	(0.799)	(0.205)	(0.057)
Mother out of labor f. (d)	-0.209	-1.279	-0.536	-3.068	-0.709 **	-0.185	0.100	-0.490
	(0.801)	(0.198)	(0.575)	(0.164)	(0.044)	(0.799)	(0.774)	(0.676)
# of children in hh	-0.501 ***	1.070 *	-0.134	-1.888 ***	0.047	-0.024	-0.317 **	0.003
	(0.004)	(0.093)	(0.423)	(0.005)	(0.574)	(0.875)	(0.016)	(0.988)
# of adults in hh	-0.305	-1.081 **	-0.991 **	-1.418	-0.157 *	-0.247	-0.044	-0.285
	(0.260)	(0.044)	(0.023)	(0.112)	(0.065)	(0.121)	(0.832)	(0.535)
Financial situation	0.181	0.159	-0.192	1.689 **	0.176	0.286 *	-0.209	0.448
	(0.435)	(0.655)	(0.511)	(0.046)	(0.245)	(0.078)	(0.288)	(0.109)
N	101	127	81	79	340	137	240	170

The table reports logit coefficients. p-value based on equation (3) in parenthesis. ***, (**), [*] signify significance of the effects at the 1%, (5%), [10%] level, respectively, based on equation (4). (d) indicates that the variable is a dummy variable. For dummy variables the reported effect refers to a discrete change of the variable from 0 to 1.

C.3 Logit results - marginal effects

Table C.4: Logit result for natives' children (marginal effects)

	Belgium	Switzerland	Czech Rep.	Estonia	France	Croatia	Luxemb.	Latvia
Intercept	-5.149	1.687	-4.715	3.802	-16.562 ***	0.260	-15.671 *	15.384 **
	(0.444)	(0.859)	(0.156)	(0.612)	(0.000)	(0.964)	(0.083)	(0.015)
Birth year	0.002	-0.001	0.003	-0.002	0.008 ***	-0.000	0.008 *	-0.008 **
	(0.469)	(0.885)	(0.127)	(0.635)	(0.000)	(0.902)	(0.098)	(0.013)
Cohort 50s (d)	-0.163 **	-0.089	-0.021	0.084	0.019	-0.078	0.031	0.037
	(0.040)	(0.409)	(0.594)	(0.346)	(0.597)	(0.248)	(0.752)	(0.602)
Cohort 60s (d)	-0.045	-0.022	0.017	0.156 ***	0.031	0.012	-0.013	0.130 ***
	(0.344)	(0.773)	(0.452)	(0.001)	(0.186)	(0.773)	(0.841)	(0.001)
Gender (d)	-0.011	0.119 ***	0.065 ***	-0.095 ***	0.042 ***	0.157 ***	0.136 ***	-0.117 ***
	(0.609)	(0.000)	(0.000)	(0.000)	(0.000)	(0.000)	(0.000)	(0.000)
Mother's age at birth	0.003	-0.029	-0.002	-0.002	0.013	-0.002	0.025	0.020
	(0.854)	(0.218)	(0.812)	(0.926)	(0.109)	(0.911)	(0.292)	(0.136)
Mother's age at b., sq (/100)	-0.005	0.047	-0.002	-0.004	-0.028 **	-0.007	-0.026	-0.024
	(0.838)	(0.193)	(0.912)	(0.879)	(0.048)	(0.753)	(0.519)	(0.264)
Father's age at birth	0.027 *	0.025	-0.003	-0.002	0.002	0.028 **	0.012	0.005
	(0.054)	(0.141)	(0.647)	(0.842)	(0.761)	(0.011)	(0.500)	(0.597)
Father's age at b., sq (/100)	-0.036 *	-0.033	0.012	0.010	0.002	-0.029 *	-0.018	-0.014
	(0.079)	(0.151)	(0.313)	(0.557)	(0.816)	(0.073)	(0.479)	(0.352)
Age difference parents	-0.006	-0.001	-0.003	-0.006 *	-0.004 *	-0.010 **	-0.002	0.003
	(0.166)	(0.866)	(0.310)	(0.092)	(0.054)	(0.017)	(0.766)	(0.474)
Mother out of labor f. (d)	-0.064 ***	-0.013	-0.058 ***	0.049	-0.011	0.045 **	0.013	0.016
	(0.004)	(0.638)	(0.002)	(0.134)	(0.291)	(0.024)	(0.662)	(0.620)
# of children in hh	-0.012 **	-0.019 **	-0.026 ***	-0.007	-0.019 ***	-0.017 **	-0.002	-0.021 ***
	(0.044)	(0.028)	(0.000)	(0.334)	(0.000)	(0.016)	(0.801)	(0.002)
# of adults in hh	-0.008	-0.016 *	-0.012 *	-0.045 ***	-0.022 ***	0.004	-0.007	-0.022
	(0.363)	(0.065)	(0.075)	(0.001)	(0.000)	(0.609)	(0.549)	(0.124)
Financial situation	0.040 ***	-0.002	0.006	0.026 *	0.021 ***	0.044 ***	0.043 ***	0.018 *
	(0.000)	(0.896)	(0.193)	(0.051)	(0.000)	(0.000)	(0.002)	(0.078)
N	1781	630	3428	836	5493	2309	1088	1316

The table reports average marginal effects. The constant term is not shown. p-value based on equation (3) in parenthesis. ***, (**), [*] signify significance of the effects at the 1%, (5%), [10%] level, respectively, based on equation (4). (d) indicates that the variable is a dummy variable. For dummy variables the reported effect refers to a discrete change of the variable from 0 to 1.

Table C.5: Logit results for migrants' children (marginal effects)

	Belgium	Switzerland	Czech Rep.	Estonia	France	Croatia	Luxemb.	Latvia
Birth year	0.018	0.008	-0.031 **	0.002	-0.003	0.003	-0.001	-0.020 **
	(0.114)	(0.166)	(0.018)	(0.889)	(0.569)	(0.751)	(0.863)	(0.034)
Cohort 50s (d)	0.185	0.055	-0.386 ***	-0.046	-0.052	0.029	-0.221	-0.263
	(0.275)	(0.573)	(0.004)	(0.847)	(0.721)	(0.881)	(0.259)	(0.143)
Cohort 60s (d)	0.136	0.066	-0.211	-0.060	-0.060	0.112	-0.012	-0.083
	(0.309)	(0.286)	(0.265)	(0.731)	(0.490)	(0.410)	(0.907)	(0.556)
Gender (d)	-0.145 **	0.038	-0.050	-0.233 ***	0.053	0.081	0.114 **	-0.079
	(0.045)	(0.352)	(0.578)	(0.000)	(0.179)	(0.270)	(0.038)	(0.134)
Mother's age at birth	0.090	-0.092	0.034	0.028	-0.006	-0.034	0.077 *	0.070
	(0.163)	(0.215)	(0.669)	(0.674)	(0.847)	(0.638)	(0.087)	(0.223)
Mother's age at b., sq (/100)	-0.073	0.218	-0.012	-0.020	-0.022	0.037	-0.170 **	-0.116
	(0.374)	(0.143)	(0.931)	(0.855)	(0.649)	(0.755)	(0.024)	(0.218)
Father's age at birth	-0.087	0.019	0.023	0.026	0.024	0.085	-0.048	-0.113
	(0.171)	(0.625)	(0.636)	(0.549)	(0.419)	(0.266)	(0.183)	(0.104)
Father's age at b., sq (/100)	0.053	-0.048	-0.070	-0.065	0.001	-0.082	0.122 **	0.197 *
	(0.416)	(0.488)	(0.269)	(0.324)	(0.979)	(0.467)	(0.037)	(0.079)
Age difference parents	0.038	0.010	0.034	0.028 *	-0.023 *	0.005	-0.015	0.026 *
	(0.339)	(0.538)	(0.230)	(0.094)	(0.091)	(0.794)	(0.202)	(0.057)
Mother out of labor f. (d)	-0.020	-0.062	-0.078	-0.244	-0.088 **	-0.022	0.016	-0.082
	(0.836)	(0.199)	(0.530)	(0.161)	(0.037)	(0.836)	(0.780)	(0.553)
# of children in hh	-0.061 ***	0.046 *	-0.015	-0.112 ***	0.006	-0.004	-0.053 **	0.000
	(0.001)	(0.098)	(0.424)	(0.001)	(0.576)	(0.872)	(0.013)	(0.981)
# of adults in hh	-0.037	-0.046 *	-0.114 **	-0.085 *	-0.020 *	-0.038	-0.007	-0.030
	(0.258)	(0.054)	(0.011)	(0.098)	(0.061)	(0.111)	(0.832)	(0.544)
Financial situation	0.022	0.007	-0.022	0.100 **	0.023	0.045 *	-0.035	0.048
	(0.436)	(0.665)	(0.510)	(0.030)	(0.244)	(0.068)	(0.289)	(0.109)
N	101	127	81	79	340	137	240	170

The table reports average marginal effects. The constant term is not shown. p-value based on equation (3) in parenthesis. ***, (**), [*] signify significance of the effects at the 1%, (5%), [10%] level, respectively, based on equation (4). (d) indicates that the variable is a dummy variable. For dummy variables the reported effect refers to a discrete change of the variable from 0 to 1.

C.4 Gelman statistics

Table C.6: Gelman statistics

	Belgium	Switzerland	Czech Rep.	Estonia	France	Croatia	Luxemb.	Latvia
	Natives' children							
Intercept	1.00	1.00	1.00	1.00	1.00	1.00	1.00	1.00
Birth year	1.00	1.00	1.00	1.00	1.00	1.00	1.00	1.00
Cohort 50s (d)	1.00	1.00	1.00	1.00	1.00	1.00	1.00	1.00
Cohort 60s (d)	1.00	1.00	1.00	1.00	1.00	1.00	1.00	1.00
Male (d)	1.00	1.00	1.00	1.00	1.00	1.00	1.00	1.00
Mother's age at birth	1.00	1.00	1.00	1.00	1.00	1.00	1.00	1.00
Mother's age at b., sq (/100)	1.00	1.00	1.00	1.00	1.00	1.00	1.00	1.00
Father's age at birth	1.00	1.00	1.00	1.00	1.00	1.00	1.00	1.00
Father's age at b., sq (/100)	1.00	1.00	1.00	1.00	1.00	1.00	1.00	1.00
Age difference parents	1.00	1.00	1.00	1.00	1.00	1.00	1.00	1.00
Mother out of labor f. (d)	1.00	1.00	1.00	1.00	1.00	1.00	1.00	1.00
# of children in hh	1.00	1.00	1.00	1.00	1.00	1.00	1.00	1.00
# of adults in hh	1.00	1.00	1.00	1.00	1.00	1.00	1.00	1.00
Financial situation	1.00	1.00	1.00	1.00	1.00	1.00	1.00	1.00
	Migrants' children							
Intercept	1.00	1.01	1.03	1.01	1.00	1.00	1.00	1.00
Birth year	1.00	1.01	1.03	1.01	1.00	1.00	1.00	1.00
Cohort 50s (d)	1.00	1.01	1.03	1.00	1.00	1.00	1.00	1.00
Cohort 60s (d)	1.00	1.00	1.02	1.00	1.00	1.00	1.00	1.00
Male (d)	1.00	1.00	1.00	1.00	1.00	1.00	1.00	1.00
Mother's age at birth	1.00	1.00	1.00	1.00	1.00	1.00	1.00	1.00
Mother's age at b., sq (/100)	1.00	1.01	1.00	1.00	1.00	1.00	1.00	1.00
Father's age at birth	1.00	1.00	1.00	1.00	1.00	1.00	1.00	1.00
Father's age at b., sq (/100)	1.00	1.00	1.00	1.00	1.00	1.00	1.00	1.00
Age difference parents	1.00	1.00	1.00	1.00	1.00	1.00	1.00	1.00
Mother out of labor f. (d)	1.00	1.00	1.00	1.00	1.00	1.00	1.00	1.00
# of children in hh	1.00	1.00	1.00	1.00	1.00	1.00	1.00	1.00
# of adults in hh	1.00	1.00	1.00	1.00	1.00	1.00	1.00	1.00
Financial situation	1.00	1.00	1.00	1.00	1.00	1.00	1.00	1.00

D Two classes upward mobility

D.1 Descriptive statistics

Table D.1: Descriptive statistics of all variables for the sample used for upward mobility

	BE	CH	DE	EE	FR	LU	LV	UK
			Natives' children					
Birth year	1964	1961	1960	1961	1965	1963	1962	1965
Cohort 50s (d)	0.38	0.48	0.55	0.50	0.33	0.43	0.45	0.32
Cohort 60s (d)	0.35	0.36	0.30	0.36	0.34	0.35	0.35	0.34
Male (d)	0.49	0.43	0.45	0.49	0.45	0.48	0.46	0.45
Father's age at birth	30.76	34.14	31.44	32.86	29.81	31.63	31.58	30.11
Mother's age at birth	28.16	29.77	28.69	29.35	26.97	27.94	28.94	27.12
Age difference parents	3.32	5.07	3.81	5.20	3.67	4.50	4.33	3.78
Mother out of labor f. (d)	0.60	0.51	0.57	0.15	0.46	0.62	0.11	0.26
# of adults in hh	2.49	2.94	2.47	2.37	2.48	2.73	2.23	2.33
# of children in hh	2.67	2.91	2.58	2.50	1.67	2.57	2.61	2.33
Financial situation	4.16	3.81	3.57	3.78	3.80	3.91	3.87	3.82
			Migrants' children					
Birth year	1969	1971	1966	1961	1969	1972	1961	1968
Cohort 50s (d)	0.28	0.13	0.37	0.46	0.20	0.08	0.45	0.20
Cohort 60s (d)	0.24	0.27	0.28	0.38	0.30	0.28	0.42	0.37
Male (d)	0.51	0.46	0.48	0.49	0.52	0.55	0.51	0.41
Father's age at birth	32.45	31.41	32.06	31.11	32.49	30.49	31.24	32.09
Mother's age at birth	27.65	27.60	27.67	29.32	27.73	27.17	29.87	28.60
Age difference parents	5.31	4.61	5.09	3.95	5.18	4.31	3.19	4.65
Mother out of labor f. (d)	0.72	0.32	0.44	0.08	0.57	0.49	0.06	0.36
# of adults in hh	2.58	2.54	2.69	2.43	2.92	2.43	2.19	2.65
# of children in hh	3.19	1.93	2.43	2.05	2.11	2.53	2.71	2.91
Financial situation	3.69	4.15	3.78	3.96	3.72	4.08	3.73	3.85

Age difference refers to the absolute age difference between the parents. Mother out of labor force is an indicator whether the respondents' mother was in or out of the labor force when the respondent was 14. # of persons in the household refers to the number of adults and children, respectively, in the household in which the respondents lived when they were 14 years old. Financial situation refers to the financial situation of the household in which the respondents lived when they were 14 years old. A detailed definition of variables can be found in the variable table A.3.

D.2 Logit coefficients

Table D.2: Logit result for natives' children

	Belgium	Switzerland	Germany	Estonia	France	Luxembourg	Latvia	UK
Intercept	43.680	-71.437	39.964	107.728 *	-25.706	-62.772	-15.094	-27.884
	(0.184)	(0.395)	(0.579)	(0.077)	(0.136)	(0.261)	(0.776)	(0.314)
Birth year	-0.024	0.033	-0.022	-0.055 *	0.012	0.027	0.004	0.012
	(0.158)	(0.437)	(0.539)	(0.072)	(0.169)	(0.338)	(0.868)	(0.375)
Cohort 50s (d)	-0.855 **	0.699	-1.050	-0.668	-0.432 **	-0.610	0.107	-0.044
	(0.022)	(0.445)	(0.188)	(0.323)	(0.030)	(0.359)	(0.850)	(0.890)
Cohort 60s (d)	-0.317	0.267	-0.456	-0.253	-0.328 **	-0.584	0.128	0.007
	(0.184)	(0.651)	(0.365)	(0.565)	(0.011)	(0.183)	(0.728)	(0.975)
Gender (d)	-0.182	0.890 ***	1.056 ***	-1.015 ***	-0.141 **	0.319	-0.991 ***	0.140
	(0.106)	(0.001)	(0.000)	(0.000)	(0.023)	(0.100)	(0.000)	(0.124)
Mother's age at birth	0.052	-0.343 *	-0.165	-0.046	0.136 **	0.395 *	0.122	0.112 *
	(0.578)	(0.093)	(0.285)	(0.730)	(0.013)	(0.054)	(0.292)	(0.099)
Mother's age at b., sq (/100)	-0.054	0.500 *	0.251	0.112	-0.201 **	-0.629 *	-0.164	-0.182 *
	(0.707)	(0.089)	(0.321)	(0.595)	(0.032)	(0.056)	(0.372)	(0.099)
Father's age at birth	0.090	0.514 ***	0.339 **	0.101	-0.028	0.109	0.184 **	0.040
	(0.295)	(0.005)	(0.013)	(0.304)	(0.527)	(0.429)	(0.028)	(0.449)
Father's age at b., sq (/100)	-0.137	-0.705 ***	-0.453 **	-0.154	0.043	-0.104	-0.254 **	-0.015
	(0.281)	(0.003)	(0.022)	(0.260)	(0.514)	(0.592)	(0.039)	(0.839)
Age difference parents	-0.015	0.081	0.047	-0.004	-0.016	-0.057	0.013	-0.023
	(0.553)	(0.219)	(0.308)	(0.882)	(0.311)	(0.308)	(0.616)	(0.286)
Mother out of labor f. (d)	-0.072	0.028	-0.100	-0.105	0.013	-0.047	0.147	0.040
	(0.516)	(0.917)	(0.639)	(0.697)	(0.836)	(0.820)	(0.552)	(0.715)
# of children in hh	-0.080 **	-0.244 ***	-0.066	-0.270 ***	-0.210 ***	-0.111	-0.196 ***	-0.059
	(0.034)	(0.010)	(0.343)	(0.001)	(0.000)	(0.134)	(0.004)	(0.140)
# of adults in hh	-0.158 ***	-0.017	0.071	-0.264 **	-0.223 ***	-0.200 **	-0.247 *	-0.177 ***
	(0.008)	(0.851)	(0.376)	(0.026)	(0.000)	(0.040)	(0.060)	(0.002)
Financial situation	0.045	0.177	-0.152 *	0.124	0.128 ***	0.125	0.158 *	0.120 ***
	(0.421)	(0.106)	(0.100)	(0.243)	(0.000)	(0.177)	(0.084)	(0.010)
N	1781	630	545	836	5493	1088	1316	2289

The table reports logit coefficients. p-value based on equation (3) in parenthesis. ***, (**), [*] signify significance of the effects at the 1%, (5%), [10%] level, respectively, based on equation (4). (d) indicates that the variable is a dummy variable. For dummy variables the reported effect refers to a discrete change of the variable from 0 to 1.

Table D.3: Logit result for migrants' children

	Belgium	Switzerland	Germany	Estonia	France	Luxembourg	Latvia	UK
Intercept	53.419	-159.820	-601.341 *	697.057 **	50.333	69.114	73.280	-84.799
	(0.786)	(0.305)	(0.051)	(0.032)	(0.471)	(0.481)	(0.706)	(0.581)
Birth year	-0.029	0.073	0.302 *	-0.360 **	-0.027	-0.042	-0.049	0.048
	(0.772)	(0.353)	(0.052)	(0.030)	(0.449)	(0.394)	(0.622)	(0.536)
Cohort 50s (d)	-1.118	1.486	6.902 *	-7.020 **	-0.622	-2.221	0.266	-0.329
	(0.638)	(0.399)	(0.068)	(0.026)	(0.451)	(0.124)	(0.905)	(0.853)
Cohort 60s (d)	-1.412	0.128	4.601	-2.874	-0.443	-1.965 **	-0.029	0.243
	(0.377)	(0.907)	(0.107)	(0.151)	(0.396)	(0.021)	(0.985)	(0.821)
Gender (d)	-0.131	1.442 **	0.324	-2.181 **	-0.357	0.979 **	-1.710 ***	-0.454
	(0.841)	(0.018)	(0.763)	(0.017)	(0.172)	(0.030)	(0.006)	(0.404)
Mother's age at birth	-0.212	0.681	-0.575	-0.768	-0.139	1.049 **	0.944	-0.740
	(0.652)	(0.398)	(0.613)	(0.331)	(0.513)	(0.015)	(0.134)	(0.105)
Mother's age at b., sq (/100)	0.405	-1.970	0.477	0.980	0.070	-1.823 ***	-1.507	0.921
	(0.544)	(0.131)	(0.815)	(0.456)	(0.819)	(0.010)	(0.145)	(0.217)
Father's age at birth	0.362	0.418	1.118	1.558 *	0.299	-0.146	0.318	0.111
	(0.447)	(0.538)	(0.213)	(0.079)	(0.148)	(0.585)	(0.496)	(0.761)
Father's age at b., sq (/100)	-0.406	0.192	-1.289	-2.185	-0.243	0.415	-0.479	0.045
	(0.466)	(0.846)	(0.346)	(0.116)	(0.324)	(0.305)	(0.498)	(0.933)
Age difference parents	0.021	-0.511	-0.565	-0.062	-0.102	-0.167	0.040	-0.117
	(0.911)	(0.212)	(0.125)	(0.699)	(0.362)	(0.173)	(0.703)	(0.367)
Mother out of labor f. (d)	0.996	0.086	-0.051	-913.875	-0.565 **	0.464	-0.006	-0.183
	(0.270)	(0.899)	(0.962)	(0.449)	(0.048)	(0.298)	(0.995)	(0.748)
# of children in hh	-0.644 **	-0.249	-0.030	-0.518	0.027	-0.352 *	-0.233	-0.015
	(0.011)	(0.456)	(0.933)	(0.295)	(0.721)	(0.062)	(0.334)	(0.938)
# of adults in hh	-1.109 **	-0.754 *	-1.331 **	-0.320	-0.172 *	-0.291	0.189	-0.213
	(0.012)	(0.069)	(0.028)	(0.585)	(0.053)	(0.288)	(0.667)	(0.383)
Financial situation	0.288	-0.244	-0.337	0.536	-0.198	-0.046	0.203	0.087
	(0.309)	(0.394)	(0.511)	(0.365)	(0.102)	(0.855)	(0.497)	(0.739)
N	101	127	54	79	340	240	170	86

The table reports logit coefficients. p-value based on equation (3) in parenthesis. ***, (**), [*] signify significance of the effects at the 1%, (5%), [10%] level, respectively, based on equation (4). (d) indicates that the variable is a dummy variable. For dummy variables the reported effect refers to a discrete change of the variable from 0 to 1.

D.3 Logit results - marginal effects

Table D.4: Logit result for natives' children (marginal effects)

	Belgium	Switzerland	Germany	Estonia	France	Luxembourg	Latvia	UK
Intercept	8.095	-7.385	6.965	15.594 *	-4.572	-5.955	-1.855	-5.666
	(0.183)	(0.393)	(0.579)	(0.075)	(0.136)	(0.262)	(0.776)	(0.314)
Birth year	-0.004	0.003	-0.004	-0.008 *	0.002	0.003	0.001	0.003
	(0.157)	(0.435)	(0.540)	(0.070)	(0.169)	(0.339)	(0.869)	(0.375)
Cohort 50s (d)	-0.148 **	0.076	-0.180	-0.096	-0.074 **	-0.056	0.014	-0.008
	(0.015)	(0.443)	(0.170)	(0.316)	(0.025)	(0.377)	(0.842)	(0.904)
Cohort 60s (d)	-0.056	0.032	-0.072	-0.034	-0.057 ***	-0.052	0.017	0.002
	(0.178)	(0.630)	(0.371)	(0.577)	(0.009)	(0.184)	(0.710)	(0.966)
Gender (d)	-0.034	0.095 ***	0.192 ***	-0.146 ***	-0.025 **	0.030	-0.118 ***	0.029
	(0.105)	(0.001)	(0.000)	(0.000)	(0.022)	(0.100)	(0.000)	(0.124)
Mother's age at birth	0.010	-0.035 *	-0.029	-0.007	0.024 **	0.038 *	0.015	0.023 *
	(0.579)	(0.093)	(0.282)	(0.732)	(0.013)	(0.056)	(0.292)	(0.099)
Mother's age at b., sq (/100)	-0.010	0.052 *	0.044	0.016	-0.036 **	-0.060 *	-0.020	-0.037 *
	(0.708)	(0.089)	(0.318)	(0.598)	(0.032)	(0.058)	(0.371)	(0.098)
Father's age at birth	0.017	0.053 ***	0.059 **	0.015	-0.005	0.010	0.023 **	0.008
	(0.294)	(0.005)	(0.012)	(0.304)	(0.528)	(0.431)	(0.027)	(0.449)
Father's age at b., sq (/100)	-0.025	-0.073 ***	-0.079 **	-0.022	0.008	-0.010	-0.031 **	-0.003
	(0.280)	(0.003)	(0.020)	(0.261)	(0.515)	(0.593)	(0.037)	(0.838)
Age difference parents	-0.003	0.008	0.008	-0.001	-0.003	-0.005	0.002	-0.005
	(0.554)	(0.221)	(0.307)	(0.880)	(0.311)	(0.307)	(0.618)	(0.287)
Mother out of labor f. (d)	-0.013	0.003	-0.018	-0.013	0.002	-0.005	0.021	0.008
	(0.514)	(0.916)	(0.636)	(0.731)	(0.836)	(0.811)	(0.524)	(0.709)
# of children in hh	-0.015 **	-0.025 ***	-0.011	-0.039 ***	-0.037 ***	-0.010	-0.024 ***	-0.012
	(0.033)	(0.009)	(0.344)	(0.001)	(0.000)	(0.136)	(0.004)	(0.139)
# of adults in hh	-0.029 ***	-0.002	0.012	-0.038 **	-0.040 ***	-0.019 **	-0.031 *	-0.036 ***
	(0.007)	(0.853)	(0.375)	(0.025)	(0.000)	(0.041)	(0.059)	(0.002)
Financial situation	0.008	0.018	-0.027 *	0.018	0.023 ***	0.012	0.020 *	0.024 ***
	(0.420)	(0.105)	(0.100)	(0.242)	(0.000)	(0.178)	(0.083)	(0.009)
N	1781	630	545	836	5493	1088	1316	2289

The table reports average marginal effects. The constant term is not shown. p-value based on equation (3) in parenthesis. ***, (**), [*] signify significance of the effects at the 1%, (5%), [10%] level, respectively, based on equation (4). (d) indicates that the variable is a dummy variable. For dummy variables the reported effect refers to a discrete change of the variable from 0 to 1.

Table D.5: Logit results for migrants' children (marginal effects)

	Belgium	Switzerland	Germany	Estonia	France	Luxembourg	Latvia	UK
Intercept	6.033	-17.267	-68.726 **	67.912 **	9.408	7.322	7.026	-15.553
	(0.786)	(0.308)	(0.027)	(0.021)	(0.471)	(0.484)	(0.705)	(0.579)
Birth year	-0.003	0.008	0.034 **	-0.035 **	-0.005	-0.004	-0.005	0.009
	(0.773)	(0.356)	(0.027)	(0.020)	(0.450)	(0.396)	(0.621)	(0.534)
Cohort 50s (d)	-0.071	0.210	0.469 ***	-0.430 ***	-0.090	-0.125 *	0.032	-0.058
	(0.738)	(0.338)	(0.003)	(0.000)	(0.482)	(0.064)	(0.875)	(0.824)
Cohort 60s (d)	-0.116	0.025	0.395 **	-0.203 *	-0.076	-0.163 ***	0.000	0.040
	(0.408)	(0.834)	(0.030)	(0.093)	(0.398)	(0.006)	(1.000)	(0.826)
Gender (d)	-0.014	0.160 **	0.041	-0.211 ***	-0.068	0.101 **	-0.152 ***	-0.086
	(0.850)	(0.013)	(0.740)	(0.008)	(0.172)	(0.025)	(0.002)	(0.400)
Mother's age at birth	-0.024	0.074	-0.067	-0.075	-0.026	0.111 **	0.090	-0.136 *
	(0.657)	(0.403)	(0.610)	(0.331)	(0.511)	(0.013)	(0.136)	(0.094)
Mother's age at b., sq (/100)	0.046	-0.214	0.055	0.096	0.013	-0.193 ***	-0.143	0.168
	(0.552)	(0.130)	(0.815)	(0.458)	(0.821)	(0.009)	(0.149)	(0.210)
Father's age at birth	0.040	0.046	0.131	0.152 *	0.056	-0.015	0.030	0.021
	(0.447)	(0.539)	(0.208)	(0.072)	(0.145)	(0.593)	(0.505)	(0.761)
Father's age at b., sq (/100)	-0.045	0.021	-0.150	-0.213	-0.046	0.044	-0.046	0.008
	(0.466)	(0.846)	(0.345)	(0.110)	(0.324)	(0.307)	(0.508)	(0.934)
Age difference parents	0.002	-0.056	-0.066	-0.006	-0.019	-0.018	0.004	-0.022
	(0.911)	(0.216)	(0.118)	(0.699)	(0.360)	(0.173)	(0.703)	(0.361)
Mother out of labor f. (d)	0.095	0.013	-0.003	-0.217 ***	-0.108 **	0.049	0.022	-0.034
	(0.267)	(0.863)	(0.979)	(0.000)	(0.047)	(0.297)	(0.829)	(0.740)
# of children in hh	-0.072 ***	-0.027	-0.004	-0.050	0.005	-0.037 *	-0.022	-0.003
	(0.005)	(0.458)	(0.932)	(0.295)	(0.721)	(0.058)	(0.337)	(0.938)
# of adults in hh	-0.124 ***	-0.082 *	-0.153 **	-0.031	-0.032 *	-0.031	0.018	-0.039
	(0.008)	(0.063)	(0.011)	(0.590)	(0.050)	(0.288)	(0.663)	(0.376)
Financial situation	0.032	-0.026	-0.039	0.052	-0.037 *	-0.005	0.019	0.016
	(0.308)	(0.387)	(0.507)	(0.372)	(0.100)	(0.854)	(0.493)	(0.738)
N	101	127	54	79	340	240	170	86

The table reports average marginal effects. The constant term is not shown. p-value based on equation (3) in parenthesis. ***, (**), [*] signify significance of the effects at the 1%, (5%), [10%] level, respectively, based on equation (4). (d) indicates that the variable is a dummy variable. For dummy variables the reported effect refers to a discrete change of the variable from 0 to 1.

D.4 Gelman statistics

Table D.6: Gelman statistics

	Belgium	Switzerland	Germany	Estonia	France	Luxembourg	Latvia	UK
	Natives' children							
Intercept	1.000	1.000	1.000	1.000	1.000	1.000	1.000	1.000
Birth year	1.000	1.000	1.000	1.000	1.000	1.000	1.000	1.000
Cohort 50s (d)	1.000	1.000	1.000	1.000	1.000	1.000	1.000	1.000
Cohort 60s (d)	1.000	1.000	1.000	1.000	1.000	1.000	1.000	1.000
Male (d)	1.000	1.000	1.000	1.000	1.000	1.000	1.000	1.000
Mother's age at birth	1.000	1.000	1.000	1.000	1.000	1.000	1.000	1.000
Mother's age at b., sq (/100)	1.000	1.000	1.000	1.000	1.000	1.000	1.000	1.000
Father's age at birth	1.000	1.001	1.000	1.000	1.000	1.000	1.000	1.000
Father's age at b., sq (/100)	1.000	1.001	1.000	1.000	1.000	1.001	1.000	1.000
Age difference parents	1.001	1.000	1.000	1.000	1.000	1.000	1.000	1.000
Mother out of labor f. (d)	1.000	1.000	1.000	1.000	1.000	1.000	1.000	1.000
# of children in hh	1.000	1.000	1.000	1.000	1.000	1.001	1.000	1.000
# of adults in hh	1.000	1.000	1.000	1.000	1.000	1.000	1.000	1.000
Financial situation	1.000	1.000	1.000	1.000	1.000	1.000	1.000	1.000
	Migrants' children							
Intercept	1.001	1.000	1.016	1.031	1.000	1.000	1.001	1.000
Birth year	1.001	1.000	1.016	1.031	1.000	1.000	1.001	1.000
Cohort 50s (d)	1.001	1.000	1.014	1.026	1.001	1.001	1.001	1.000
Cohort 60s (d)	1.000	1.001	1.013	1.020	1.000	1.000	1.000	1.000
Male (d)	1.000	1.000	1.000	1.002	1.000	1.000	1.000	1.000
Mother's age at birth	1.000	1.001	1.000	1.000	1.000	1.001	1.000	1.000
Mother's age at b., sq (/100)	1.000	1.000	1.000	1.000	1.000	1.001	1.001	1.000
Father's age at birth	1.000	1.001	1.001	1.001	1.001	1.000	1.000	1.000
Father's age at b., sq (/100)	1.000	1.000	1.000	1.001	1.000	1.000	1.000	1.000
Age difference parents	1.000	1.003	1.001	1.000	1.000	1.000	1.000	1.000
Mother out of labor f. (d)	1.000	1.000	1.001	2.044	1.000	1.000	1.000	1.000
# of children in hh	1.000	1.000	1.000	1.001	1.000	1.000	1.000	1.000
# of adults in hh	1.001	1.000	1.000	1.001	1.000	1.000	1.000	1.000
Financial situation	1.000	1.000	1.000	1.001	1.001	1.000	1.000	1.000

E Selection

E.1 Dropped observations

Table E.1: Sample sizes

		**Full sample in EU-SILC**	born in country of residence	finished education	parental information available	**resulting sample**	nonmissing observations	**upward mobility**	without ISCED 5-6 parental educ	**downward mobility**	without illiterate parents	**conditional upward mobility**	max. ISCED 0-2 parental educ
		N	percentage dropped (%)			N	(%)	(%)	N	(%)	N	(%)	N
AT	total	13933	-29%	-10%	-44%	5019	-5%	-16%	3990	0%	4745		
	nat					4905	-5%	-16%	3903	0%	4639		
	mig					114	-7%	-18%	87	0%	106		
BE	total	14300	-33%	-12%	-47%	4458	-8%	-26%	3051			-54%	1882
	nat					4300	-8%	-27%	2911			-55%	1781
	mig					158	-7%	-5%	140			-31%	101
CH	total	17602	-39%	-12%	-47%	4982	-11%	-15%	3736	0%	4395	-83%	757
	nat					4604	-11%	-15%	3455	0%	4078	-85%	630
	mig					378	-11%	-16%	281	-5%	317	-62%	127
CZ	total	20629	-18%	-10%	-59%	6247	-1%	-11%	5518			-43%	3509
	nat					6142	-1%	-11%	5415			-44%	3428
	mig					105	0%	-2%	103			-23%	81
DE	total	28644	-23%	-9%	-47%	10568	-23%	-32%	5514	0%	8154		
	nat					10304	-23%	-33%	5361	0%	7955		
	mig					264	-25%	-23%	153	0%	199		
EE	total	13426	-28%	-16%	-50%	4071	-14%	-25%	2617	0%	3505	-74%	915
	nat					3653	-14%	-24%	2378	0%	3148	-73%	836
	mig					418	-15%	-33%	239	0%	357	-78%	79
FR	total	27071	-28%	-9%	-46%	9520	-15%	-15%	6886	-1%	8013	-28%	5833
	nat					8980	-15%	-15%	6466	0%	7605	-28%	5493
	mig					540	-17%	-7%	420	-9%	408	-24%	340
HR	total	16948	-24%	-9%	-53%	5507	-16%	-8%	4243	0%	4609	-47%	2446
	nat					5225	-16%	-8%	4024	0%	4374	-47%	2309
	mig					282	-16%	-7%	219	0%	235	-42%	137
LU	total	14891	-57%	-14%	-40%	3317	-5%	-10%	2835	0%	3142	-58%	1328
	nat					2938	-5%	-10%	2503	0%	2784	-61%	1088
	mig					379	-5%	-8%	332	-1%	358	-33%	240
LV	total	15891	-28%	-11%	-55%	4618	-8%	-19%	3468	0%	4251	-65%	1486
	nat					4079	-8%	-19%	3059	0%	3751	-65%	1316
	mig					539	-7%	-18%	409	0%	500	-66%	170
UK	total	18670	-29%	-9%	-56%	5315	-14%	-22%	3582				
	nat					5145	-14%	-22%	3466				
	mig					170	-13%	-22%	116				

The table shows the shares of observations dropped to obtain the final samples. We restrict the sample to persons born in their country of residence, with finished education. We have to drop observations with missing information on the respondents' parents and household characteristics at the age of 14 (i.e. if information on parental country of birth or education is missing, if parents are coded as dead or unknown in EU-SILC or the respondent was living in a collective household or institution). From the resulting sample we have to drop all individuals with missing information on the dependent or independent variables used in the empirical analysis. For the analysis of upward mobility we exclude respondents whose more highly educated parent already reached the highest education level (ISCED 5-6). For the analysis of downward mobility we exclude respondents whose parents are illiterate. For the analysis of upward mobility conditional on parental education we focus on respondents whose more highly educated parent has an education level corresponding to ISCED 0-2. Country-codes: AT=Austria, BE=Belgium, CH=Switzerland, CZ=Czech Republic, DE=Germany, EE=Estonia, FR=France, HR=Croatia, LU=Luxemburg, LV=Latvia, UK=United Kingdom.

E.2 Mobility patterns of dropped observations

Table E.2: Mobility of dropped individuals due to missing values for explanatory variables

	upward			downward			upward cond			upward 2 classes		
	migrants	natives	mobility gap	migrants	natives	mobility gap	migrants	natives	mobility gap	migrants	natives	mobility gap
Sample without dropping observations												
AT	51%	44%	6%	14%	11%	3%						
BE	72%	61%	11%				76%	70%	6%	21%	25%	-3%
CH	61%	38%	23%	8%	8%	-1%	90%	84%	7%	16%	14%	3%
CZ	69%	66%	3%				77%	90%	-13%			
DE	50%	39%	11%	10%	14%	-4%				28%	24%	4%
EE	46%	48%	-2%	21%	16%	5%	81%	81%	0%	17%	19%	-3%
FR	81%	77%	5%	3%	5%	-2%	81%	80%	1%	26%	24%	2%
HR	54%	49%	5%	6%	6%	0%	67%	68%	0%			
LU	59%	40%	19%	7%	13%	-7%	72%	57%	14%	15%	11%	4%
LV	47%	52%	-5%	14%	13%	1%	84%	79%	4%	13%	15%	-2%
UK	87%	72%	16%							44%	28%	16%
Dropped observations												
AT	88%	39%	49%	0%	12%	-12%						
BE	78%	49%	29%				83%	55%	29%	0%	14%	-14%
CH	54%	39%	15%	17%	9%	8%	75%	83%	-8%	10%	15%	-5%
CZ		78%	-78%					93%	-93%			
DE	39%	36%	3%	20%	13%	7%				20%	20%	0%
EE	50%	43%	7%	20%	15%	4%	78%	70%	8%	4%	17%	-12%
FR	77%	74%	3%	4%	4%	0%	72%	76%	-4%	14%	17%	-3%
HR	50%	52%	-2%	5%	5%	-1%	56%	65%	-9%			
LU	56%	32%	24%	6%	19%	-14%	83%	48%	35%	8%	4%	4%
LV	48%	49%	-1%	10%	13%	-3%	83%	73%	10%	0%	5%	-5%
UK	68%	70%	-2%							31%	18%	12%
Final sample												
AT	47%	44%	3%	15%	11%	4%						
BE	71%	62%	9%				75%	71%	4%	23%	26%	-3%
CH	62%	38%	24%	6%	8%	-2%	93%	84%	9%	17%	13%	4%
CZ	69%	66%	3%				77%	90%	-13%			
DE	54%	40%	14%	7%	15%	-7%				31%	27%	5%
EE	45%	48%	-4%	21%	16%	5%	82%	84%	-1%	20%	20%	0%
FR	82%	77%	5%	3%	5%	-2%	83%	80%	2%	29%	25%	4%
HR	55%	48%	6%	6%	6%	0%	70%	68%	2%			
LU	59%	40%	18%	7%	13%	-6%	71%	58%	13%	15%	12%	4%
LV	47%	52%	-6%	15%	13%	1%	84%	80%	4%	14%	16%	-2%
UK	91%	72%	19%							47%	30%	17%

The table shows mobility patterns of natives and second generation migrants. The *sample without dropping observations* refers to mobility patterns present in the sample before dropping individuals with missing information on any explanatory variable used in the multivariate analysis of the paper. The panel labeled *dropped observations* summarizes the mobility patterns of natives and second generation migrants of individuals that are dropped due to missing information on any explanatory variable used. The panel labeled *final sample* summarizes the mobility patterns of the sample after dropping individuals with missing values on explanatory variables, which is used in the multivariate analysis in the paper.

Table E.3: Summary of mobility of dropped individuals

	upward			downward			upward cond			upward 2 classes		
	migrants	natives	bias in mobility gap	migrants	natives	bias in mobility gap	migrants	natives	bias in mobility gap	migrants	natives	bias in mobility gap
	Dropped observations and potential bias in mobility gaps											
AT	88%	39%	-4%	0%	12%	1%						
BE	78%	49%	-1%				83%	55%	-2%	0%	14%	0%
CH	54%	39%	1%	17%	9%	-1%	75%	83%	2%	10%	15%	1%
CZ		78%	0%					93%	0%			
DE	39%	36%	3%	20%	13%	-3%				20%	20%	0%
EE	50%	43%	-2%	20%	15%	0%	78%	70%	-2%	4%	17%	3%
FR	77%	74%	0%	4%	4%	0%	72%	76%	1%	14%	17%	1%
HR	50%	52%	1%	5%	5%	0%	56%	65%	2%			
LU	56%	32%	0%	6%	19%	0%	83%	48%	-1%	8%	4%	0%
LV	48%	49%	0%	10%	13%	0%	83%	73%	-1%	0%	5%	0%
UK	68%	70%	3%							31%	18%	0%

The table shows mobility patterns of natives and second generation migrants that are dropped from the sample due to missing information on any of the explanatory variables used in the multivariate analysis in the paper. *Migrants* refers to the mobility of second generation migrants, *natives* refers to the mobility of natives. The *bias in mobility gap* is calculated as the difference between mobility gaps for the sample before dropping individuals with missing information on explanatory variables and mobility gaps in the final sample after dropping those individuals, which is used in the analyses in the paper. The bias in mobility gap indicates by how much larger or smaller the mobility gap detected in the data would be if those excluded observations were included in the multivariate analysis. Negative values for the bias in mobility gap indicate a downward bias, i.e. the identified mobility gaps would be larger (or less negative) than the ones reported in the multivariate analysis (migrants children would be relatively more mobile than reported), positive values indicate an upward bias, i.e. mobility gaps would be smaller (or more negative) than reported (i.e. migrants' children would be relatively less mobile than reported). Differences to table E.2 are due to rounding.
